# Evolutionary dynamics of plant cell wall-degrading enzymes reflects feeding ecology in weevils

**DOI:** 10.1038/s42003-026-10927-0

**Published:** 2026-09-23

**Authors:** Na Ra Shin, Roy Kirsch, Heiko Vogel, Alexander Riedel, Martin Kaltenpoth, Yannick Pauchet

**Affiliations:** 1 Department of Entomology, Max Planck Institute for Chemical Ecology, Jena, Germany; 2 Department of Insect Symbiosis, Max Planck Institute for Chemical Ecology, Jena, Germany; 3 Department of Biological Sciences and Center for Biodiversity Research, University of Memphis, Memphis, TN USA; 4 Museum of Natural History Karlsruhe, Karlsruhe, Germany; 5 Present Address: Korea Institute of Science and Technology, Seoul, South Korea

**Keywords:** Molecular ecology, Evolutionary ecology

## Abstract

Many herbivorous beetles depend on plant cell wall-degrading enzymes (PCWDEs) to access the nutritious plant cell content. However, the evolution of PCWDEs in weevils, the most diverse lineage of herbivores, remains poorly understood. Using transcriptomic and genomic analyses of 45 weevil species spanning the majority of existing subfamilies, we identified a total of 13 different PCWDE families, revealing high variability between species. Interestingly, the PCWDE repertoires tracked dietary specialization, with convergent reductions in fungivorous taxa. Furthermore, PCWDE gene family dynamics were driven by both larval and adult specialized herbivory on different plant organs. Despite this complex evolutionary history, phylogenetic analyses place core PCWDE functionalities at the base of the Phytophaga (weevils, leaf beetles, and longhorn beetles) and reveal subsequent horizontal gene transfer (HGT) events from various donors during early Phytophaga diversification. These findings demonstrate that HGTs, gene duplications, and losses shaped PCWDE diversity and facilitated the ecological success of weevils.

## Abstract (web summary)

Plant cell wall degrading enzymes (PCWDEs) track feeding ecology across weevils, revealing how horizontal gene transfer and gene family turnover contribute to dietary specialization.

## Introduction

One key to the evolutionary success of many insect lineages is their ability to feed on plants. However, herbivory entails various challenges due to repellent or toxic plant secondary metabolites^1,2^, an often-imbalanced nutrient composition, and the recalcitrant nature of the plant cell wall^3,4^. Concordantly, herbivorous insects have evolved a plethora of specialized adaptations to cope with the toxic defenses and overcome the nutritional limitations and digestive challenges associated with a purely plant-based diet^5,6^. The high selective pressures exerted by herbivores in turn favored the diversification of plant nutritional composition and defensive chemistry. Consequently, the ensuing co-evolutionary arms race between plant defenses and herbivore counter-adaptations is thought to be the driving factor behind the spectacular adaptive radiation of plants and herbivorous insects^7,8^.

To break open the recalcitrant plant cell wall and gain access to the nutritious cell content, many herbivorous insects rely on plant cell wall-degrading enzymes (PCWDEs) that target cellulose, hemicelluloses, and pectins as the main building blocks of plant cell walls^9^. PCWDEs can be assigned to families of glycoside hydrolases (GH), polysaccharide lyases (PL) and carbohydrate esterases (CE), collectively representing a substantial fraction of catabolic Carbohydrate-Active enZymes (CAZymes)^10,11^. In some insect taxa, e.g., in termites, cockroaches and certain beetles, PCWDEs are secreted by symbionts localized in the gut or in specialized gut-associated organs^12–16^. Additionally, PCWDEs were also found to be encoded in the insects’ own genomes^11^. Interestingly, many of these insect-encoded PCWDEs were acquired by horizontal gene transfer (HGT) events that occurred multiple times independently in herbivorous insect clades like stick insects, bugs, wasps and beetles^17,18^.

In beetles, herbivory evolved at least seven times independently^19^, and PCWDEs were reported as key innovations for survival, dietary specialization and evolutionary success of herbivorous beetle lineages ^20,21^. In particular, the Phytophaga, the largest clade of herbivorous beetles comprising weevils, longhorned beetles, and leaf beetles appears to “have evolved an inordinate fondness for PCWDEs”^22^. Within this clade, leaf beetles and longhorned beetles (superfamily Chrysomeloidea) were studied comprehensively regarding the sampling of representative taxa (covering 18 out of 20 subfamilies) and functional characterization of PCWDEs. These studies revealed several widespread PCWDEs (cellulases of the families GH45 and GH48) that were most likely present in the common ancestor of the Phytophaga including weevils ^20,23^, but also gene families that show a patchy distribution across Phytophaga (e.g., pectolytic PL4, hemicellulolytic GH5 and GH10) or experienced a dynamic evolutionary history characterized by multiple HGT events and symbiont gains and losses (pectolytic GH28)^24^.

By contrast, our understanding of the distribution and function of PCWDEs in weevils remains fragmentary, primarily focusing on species within the family Curculionidae, and particularly the subfamilies Dryophtorinae and Scolytinae^25–27^. While McKenna et al.^20^ provided additional insights, significant gaps remain, especially concerning PCWDEs in the “primitive weevil families” outside of Curculionidae, as only 14 weevil subfamilies (which is about half of the weevil subfamilies, depending on the classification) have been investigated for their PCWDEs so far. Filling these gaps is especially important in light of the large diversity of dietary specializations found in weevils. Whereas the vast majority of leaf and longhorned beetles feed on photosynthetic and vascular tissue and show conservatism regarding plant tissue^28^, weevils are known to exploit a diverse array of diets including leaves, wood, plant sap, starchy seeds and tubers, mixtures of wood and fungi, or exclusively fungal mycelia^29^. Moreover, repeated shifts between diets have been reported^30^. Thus, a comprehensive characterization of PCWDEs across weevils can be expected to yield valuable insights into the evolutionary dynamics of these important digestive enzymes and their relevance for dietary shifts and the ecological diversification of the most diverse insect superfamily on the planet.

In order to elucidate the distribution of PCWDEs in weevils and unravel their evolutionary history in the Phytophaga, we used genomic and transcriptomic data to analyze PCWDE-encoding genes across 45 weevil species, covering 24 out of 26-28 subfamilies of Curculionoidea. Our findings unveiled the presence of 13 families of PCWDEs, comprising cellulolytic, hemicellulolytic, and pectolytic enzymes, including the first description of putative endo-α-1,5-L-arabinanases (GH43_6) in animals. We illustrate how HGTs, gene duplications, and massive gene losses have shaped the evolution of PCWDEs in weevils. In particular, multiple independent dietary shifts from herbivory to fungivory in weevils coincided with massive PCWDE gene losses. Furthermore, larval but also adult diet is a predictor of the PCWDE repertoire, indicating that the nutritional ecology of both stages drives the evolution of plant cell wall degrading capabilities in weevils. Through a comprehensive phylogenetic investigation, our study significantly advances the current understanding of PCWDE dynamics in beetles and its implications for the evolutionary success of the megadiverse Phytophaga clade.

## Results

### Species phylogeny

We sequenced the transcriptomes of 21 weevil species and retrieved Phytophaga transcriptome and genome sequence data for an additional 24 species from public databases (Supplementary Table S1). Our dataset therefore encompasses 45 species including representatives from 24 subfamilies (see methods section for details), covering all extant families within Curculionoidea, except for Caridae and Nemonychidae (following Shin et al.^31^ in assigning *Cimberis*, formerly in Nemonychidae, to the distinct family Cimberididae). Species identifications were validated by extracting cytochrome oxidase I (COI) barcodes from the transcriptomes, whenever possible (Supplementary Table S2), and cross-referencing them with public databases using BLAST. No discrepancies were found between identifications based on COI barcodes and morphological characters for the species collected by us and those retrieved from public databases, except for a transcriptome dataset obtained from NCBI for *Curculio bimaculatus* (SRA accession: SRR11781473). BLAST searches against both NCBI_nr and the Barcode of Life Data System (BOLD; https://v4.boldsystems.org/) revealed that the extracted barcode best matched species of the subfamily Entiminae. Thus, we treated it as “Gen. sp. Entiminae” in our analyses. The transcriptomes and genomes varied in completeness, as assessed using BUSCO with the insect_odb10 database. BUSCO completeness ranged from 49.6% in the transcriptome of *Agnesiotis pilosula* to 99.5% in the genome of *Rhynchophorus ferrugineus* (Supplementary Table S3).

**Fig. 1:**
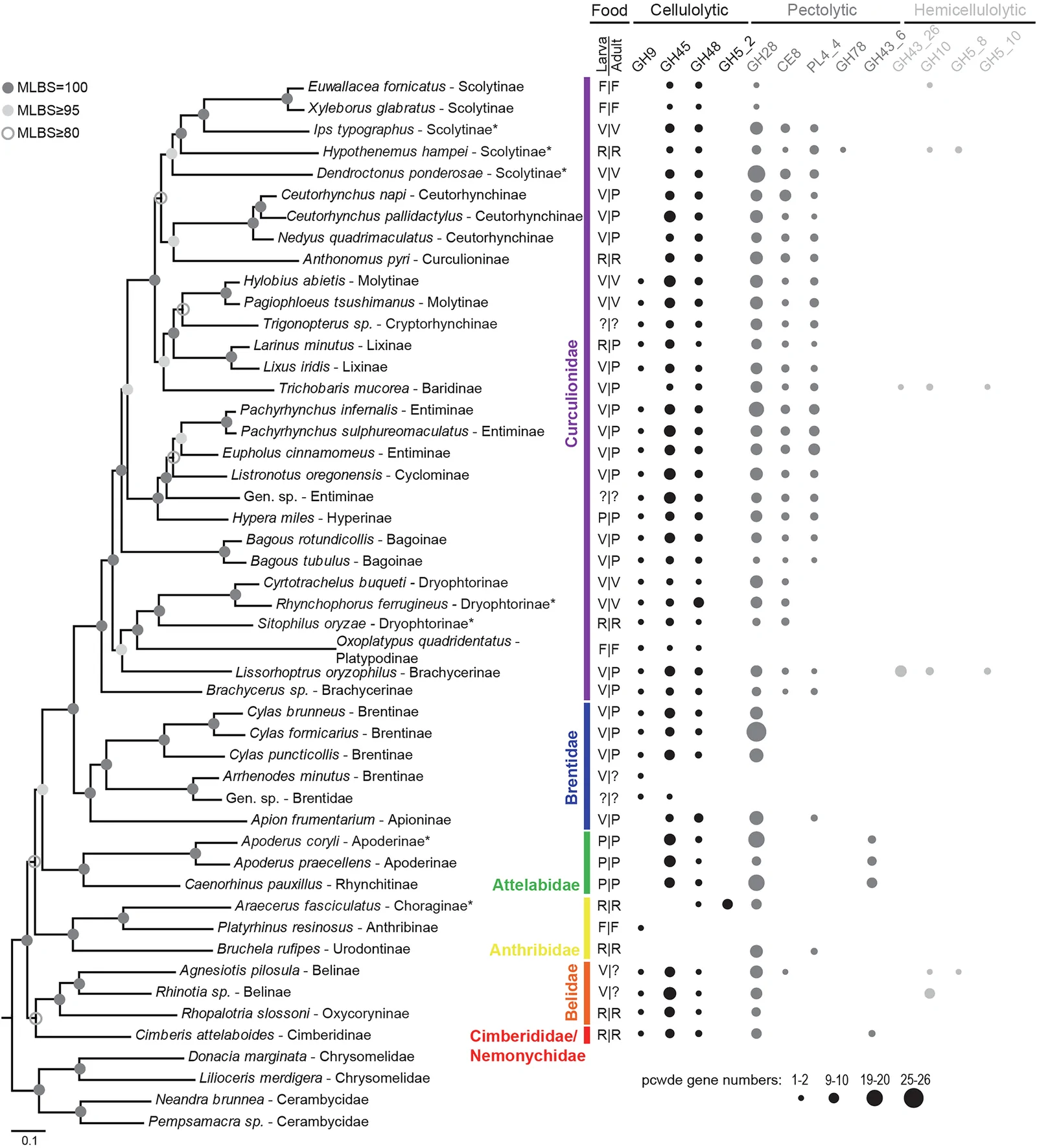
Distribution of plant cell wall-degrading enzyme (PCWDE) families in weevils. A maximum likelihood analysis, based on a codon-based nucleotide sequence alignment of 107 BUSCO single copy orthologous genes (containing 78,321 sites) common to all genome and transcriptome datasets was conducted using the program IQ-TREE with 1000 ultrafast-bootstrap replicates. The optimal nucleotide evolution model, determined by IQ-TREE, was the generalized time reversible (GTR) model, incorporating empirical base frequencies count (+F) and FreeRate model (+R8). Support values (MLBS: Maximum Likelihood Bootstrap Support) are denoted by dark gray (MLBS = 100%), light gray (MLBS ≥ 95%), or open circles (MLBS ≥ 80%). The dataset comprises 45 weevil species as the ingroup and four Chrysomeloidea species as the outgroup. Species for which draft genomes were available are highlighted with asterisks. Larval and adult food sources are indicated next to each species by defining four feeding categories: F, fungi; P leaves; V, vascular tissue; R, reproductive organs. PCWDE-encoding genes were manually curated, classified as glycoside hydrolases (GH), carbohydrate esterases (CE), and polysaccharide lyases (PL), and further categorized based on their putative substrate. Their presence and gene numbers are indicated by circles below each PCWDE family next to the species tree.

We inferred a species phylogeny using orthologous genes from 49 transcriptomes and genomes, including 45 Curculionoidea as well as four Chrysomeloidea as outgroups. Using the insect_odb10, we retrieved 107 orthologous genes across these 49 species, generating a codon-based super-alignment of 78,321 sites for maximum likelihood (ML) phylogenetic inference (Fig. 1). All families of weevils were recovered as monophyletic. Within the true weevils (Curculionidae), the relationships of early diverging clades aligned with prior molecular studies^31–33^. Our findings confirm the paraphyly of Brachycerinae: while *Brachycerus* is sister to the remaining higher weevils, *Lissorhoptrus oryzophilus* (currently classified as Brachycerinae) is more closely related to the subfamilies Dryophthorinae and Platypodinae (*Oxoplatypus*). Species of the subfamily Bagoinae (*Bagous tubulus* and *B. rotundicollis*) were recovered with maximum bootstrap support as sister to a clade comprising most higher Curculionidae subfamilies, consistent with previous studies^31–33^. Within the higher Curculionidae, species formed four major groups: (i) *Hypera miles* (Hyperinae) clustered with species of Entiminae and *Listronotus* (Cyclominae); (ii) *Trichobaris mucorea* (Baridinae) was recovered with species of Lixinae and Molytinae, alongside *Trigonopterus*, the sole representative of the subfamily Cryptorhynchinae in our analyses; (iii) *Anthonomus pyri* (Curculioninae) appeared as the sister taxon to three species of Ceutorhynchinae; (iv) and five species of bark beetles (Scolytinae) were recovered as monophyletic (Fig. 1). The first group corresponds to the so called CEGH-clade, while the latter three groups represent the so called CCCMS-clade ^31^, both maximally supported in our analysis. As in previous phylogenies, these clades include tribes from subfamilies Cyclominae, Entiminae, Gonipterini, Hyperinae (CEGH clade) and Curculioninae, Baridinae, Ceutorhynchinae, Cossoninae, Molytinae, Scolytinae (CCCMS clade)^34^.

### Distribution of families of PCWDEs across weevils: the core set

Screening 45 weevil transcriptomes and genomes revealed 13 PCWDE families including genes predicted to encode cellulolytic, pectolytic, and hemicellulolytic enzymes. Notably, cellulolytic GH9, GH45 and GH48, along with the pectolytic family GH28 (also called polygalacturonases or PGs), were present in nearly all species analyzed (Fig. 1; Supplementary Table S1), forming the core set of PCWDEs in weevils.

**Fig. 2:**
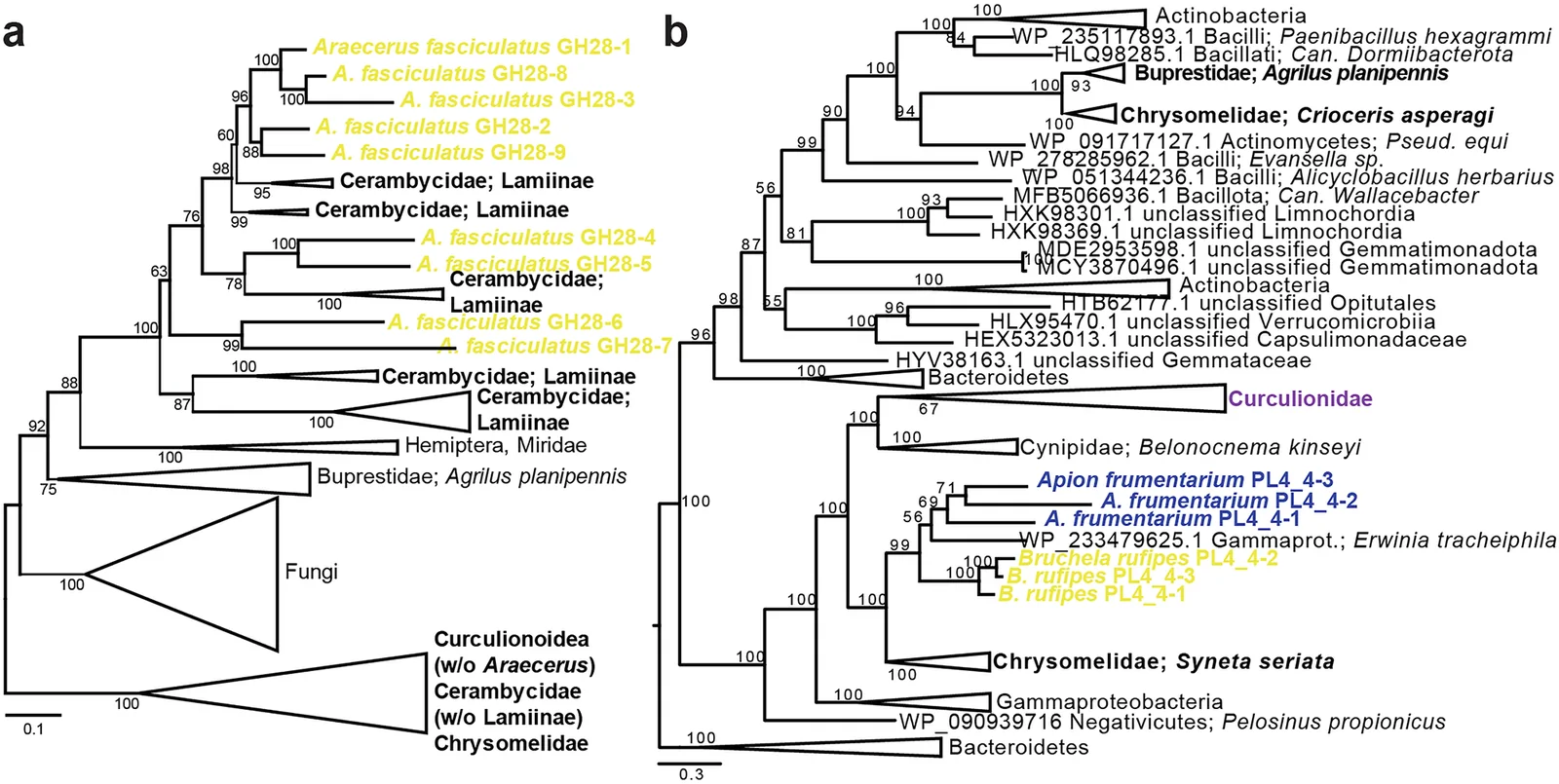
Phylogenetic relationships of weevil pectolytic GH28 and PL4 enzymes. **a** GH28 sequences, obtained from *A. fasciculatus* (depicted in yellow), as well as other weevils, leaf beetles (Chrysomelidae), longhorned beetles (Cerambycidae: Lamiinae), the jewel beetle *A. planipennis* (Buprestidae), mirid bugs (Heteroptera, Miridae), and fungi, were subjected to a maximum likelihood (ML) phylogenetic analysis. The WAG model, incorporating a FreeRate model with six categories (+R6), was identified as the most suitable model for protein evolution. UltraFast Bootstrap values are provided next to branches, and the tree is rooted at midpoint. Note that the *Araecerus* GH28 sequences do not form a monophyletic cluster but instead segregate into three distinct groups within a larger clade of sequences from longhorned beetles of the subfamily Lamiinae. **b** Phylogenetic relationships of weevil-derived PL4 rhamnogalacturonate lyases, along with those of the gall wasp *B. kinseyi* and bacteria, were analyzed using maximum likelihood (ML). The “Le and Gascuel” (LG) model, incorporating a FreeRate model with six categories (+R6), was determined to be the most suitable model for protein evolution. UltraFast Bootstrap values are provided next to the branches, and the tree is rooted at midpoint. Interestingly, the PL4s derived from species of Curculionidae (purple) form a distinct cluster, separated from those of *A. frumentarium* (Brentidae, blue) and *Bruchela* (Anthribidae, yellow). These findings indicate the occurrence of at least two, and most likely three, independent origins of PL4 enzymes during the evolutionary history of weevils.

GH9, GH45 and/or GH48 (predicted cellulases) were found across all Curculionoidea with the exception of *Bruchela rufipes* (Anthribidae, Urodontinae), supporting the early origin of these genes and their acquisition via individual HGT events. GH28 (predicted pectinases) showed a more dynamic evolutionary history, with three independent losses of pectolytic enzymes, two of which occurred in taxa with fungivorous larvae and adults. A phylogenetic analysis of weevil GH28 sequences revealed that most of them cluster with other Phytophaga GH28s, suggesting a common evolutionary origin^35^. However, the expanded set of GH28 sequences from *Araecerus fasciculatus* (Anthribidae) clustered with Lamiinae (Cerambycidae) rather than with other Curculionoidea (Fig. 2a), suggesting different origins of *Araecerus* GH28s. Genome sequencing revealed flanking regions showing conserved insect-specific synteny, confirming insect-derived origin of *Araecerus* GH28s (Supplementary Fig. S1). Furthermore, GH28 sequences from Lamiinae and *A. fasciculatus* were closely related to those of mirid bugs (Hemiptera; Miridae) and the emerald ash borer, *Agrilus planipennis* (Coleoptera; Buprestidae), all of which formed a sister clade to fungal homologs from the Ascomycota clade (Fig. 2a). This pattern suggests that Ascomycota-like GH28s were either acquired multiple times independently via HGT from fungi in different insect lineages, or horizontally transferred between insects. By comparing GH28 gene structures among *A. planipennis*, mirid bugs (*Adelphocoris suturalis*), *Araecerus*, and the Lamiinae species *Anoplophora glabripennis*, we identified a highly conserved intron in the 5’-UTR regions (Supplementary Fig. S2), suggesting a shared regulatory mechanism and supporting a common evolutionary origin.

### PL4 and CE8: characteristic of the family Curculionidae

In addition to GH9, GH45, GH48, and GH28, pectin methylesterases from the carbohydrate esterase family 8 (CE8) and rhamnogalacturonan lyases belonging to the polysaccharide lyase family 4 subfamily 4 (PL4_4) were among the most widely distributed PCWDEs in weevils (Fig. 1; Supplementary Table S1). These enzyme families appear to be restricted to the Curculionidae, with only a few exceptions: CE8 sequences were found in a single species outside Curculionidae (*Agnesiotis pilosula*; Belidae), while multiple paralogs of PL4_4 were detected in *Apion frumentarium* (Brentidae) and *Bruchela* (Anthribidae), respectively. Phylogenetic analyses were used to determine whether the distribution of these gene families resulted from gene losses following a common acquisition or from multiple independent HGT events. Curculionidae CE8s likely originated from a single HGT event from a bacterial donor, as all sequences grouped together (Supplementary Fig. S3), confirming previous analyses^36^. Although genome data from *A. pilosula* are missing thus preventing us from comparative CE8 gene structure analysis, our phylogenetic analysis is indicative of shared ancestry in the entire Curculionoidea.

Weevil PL4 sequences showed high similarity to bacterial homologs, and phylogenetic analysis confirmed their close relationship (Fig. 2b). However, weevil PL4s did not form a monophyletic group; instead, sequences clustered into three distinct clades: (1) Curculionidae-derived sequences closely related to PL4 in a gall wasp, (2) sequences from *Apion* (Brentidae), and (3) sequences from *Bruchela* (Anthribidae) (Fig. 2b). This pattern suggests that PL4 genes were acquired independently from different Gammaproteobacterial donors through at least three separate HGT events in weevils. The phylogenetic placement of other Coleoptera PL4 genes revealed a similar pattern: PL4 genes in leaf beetles originated from multiple HGT events, with Synetinae PL4 genes clustering within a bacterial clade alongside weevil PL4 from Brentidae and Anthribidae, while Criocerinae PL4 genes grouped with Buprestidae PL4 in a separate bacterial clade (Fig. 2b). Notably, PL4 sequences generally clustered within family or subfamily groups, with no evidence of shared orthologs. This grouping is also reflected by family/subfamily-specific intron positions (Supplementary Fig. S4). Collectively, these findings indicate that Phytophaga PL4 genes originated from multiple independent HGT events from bacterial donors.

### Evolutionary history of patchily distributed PCWDE families

Some PCWDE families exhibited a patchier distribution across weevil species (Fig. 1; Supplementary Table S1). These included GH78 putative rhamnosidases, GH43_6 arabinases, GH10 xylanases, GH43_26 arabinofuranosidases, GH5_2 cellulases, and GH5_8 and GH5_10 mannanases. Notably, GH78 and GH5_2 were exclusively found in the genomes of the coffee berry borer (*Hypothenemus hampei*) and the coffee bean weevil (*A. fasciculatus*), respectively. After finishing our data analysis, we also found a GH78 in the recently released draft genome of *Euwallacea similis* (that is not included in our species phylogeny), but not in the genome or transcriptome of *E. fornicatus*. GH10 xylanases were detected in two Belidae species and five Curculionidae species. GH43_26 arabinofuranosidases and GH5_10 mannanases were restricted to two Curculionidae species, *Lissorhoptrus oryzophilus* and *Trichobaris mucorea*. Lastly, GH43_6 arabinases were found only in *Cimberis attelaboides* and three Attelabidae species (Fig. 1; Supplementary Table S1). To investigate the evolutionary histories of these rarely encountered PCWDEs as well as to reconstruct the repertoire of PCWDEs in the Phytophaga ancestor, we analyzed GH10, GH5_10 and GH43_26 hemicellulases as well as pectolytic GH78 across Phytophaga, reconstructing their phylogeny and assessing their gene structure (Supplementary Fig. S5).

GH10 genes displayed a sporadic distribution not only in weevils but also in leaf beetles (Criocerinae and Cassidinae) and longhorned beetles (Cerambycinae and Lamiinae). In contrast, GH5_10 genes were absent from longhorned beetles and, aside from the two cases in weevils, were only found in leaf beetles (Bruchinae)^24^. Despite their scattered presence, both gene families exhibited a high degree of conservation, as reflected by phylogenetic clustering and identical intron positions (Supplementary Fig. S5a, b). Two intron positions were conserved across all beetle GH10 genes, supporting a common ancestry for Phytophaga GH10, likely originating from Actinomycetia (Supplementary Fig. S6a). Similarly, a conserved intron position was also identified in GH5_10 genes of leaf beetles^37^. The gene structure of weevil GH5_10 genes remain unknown due to a lack of available genome data. BLAST searches revealed that GH5_10 is also present in arthropods outside Coleoptera, including Crustacea and Chelicerata, as well as in several other insect lineages such as Collembola and Ephemeroptera. Phylogenetic analyses showed that Coleoptera-derived GH5_10 genes cluster with those from oribatid mites (Chelicerata), while other animal-derived GH5_10 proteins form separate clades (Supplementary Fig. S5b). This pattern suggests that GH5_10 genes have multiple independent evolutionary origins in animals, further supporting previous findings in Phytophaga beetles^37^.

Like GH10, GH43_26 is patchily present in every major Phytophaga lineage including weevils, longhorned and leaf beetles^24,38^, as well as in jewel beetles (Buprestidae) ^20^. However, in contrast to the aforementioned GH10 gene family, GH43_26 beetle sequences cluster in two well separated clades: (1) leaf beetle sequences of the subfamily Criocerinae are closely related to Bacteroidota counterparts and (2) all the other coleopteran sequences form a sister clade to spider mite sequences, together nesting in a bacterial clade of Actinomycetia (Supplementary Fig. S5c) indicating independent acquisitions within beetles. Similarly, independent acquisitions within Coleoptera likely explain the patchy distribution of GH78, strongly indicated by a weevil- and a leaf beetle-specific clade, nesting within counterparts that belong to different bacterial classes, respectively (Supplementary Fig. S5d).

### GH43_6: a newly identified enzyme family in animals

**Fig. 3:**
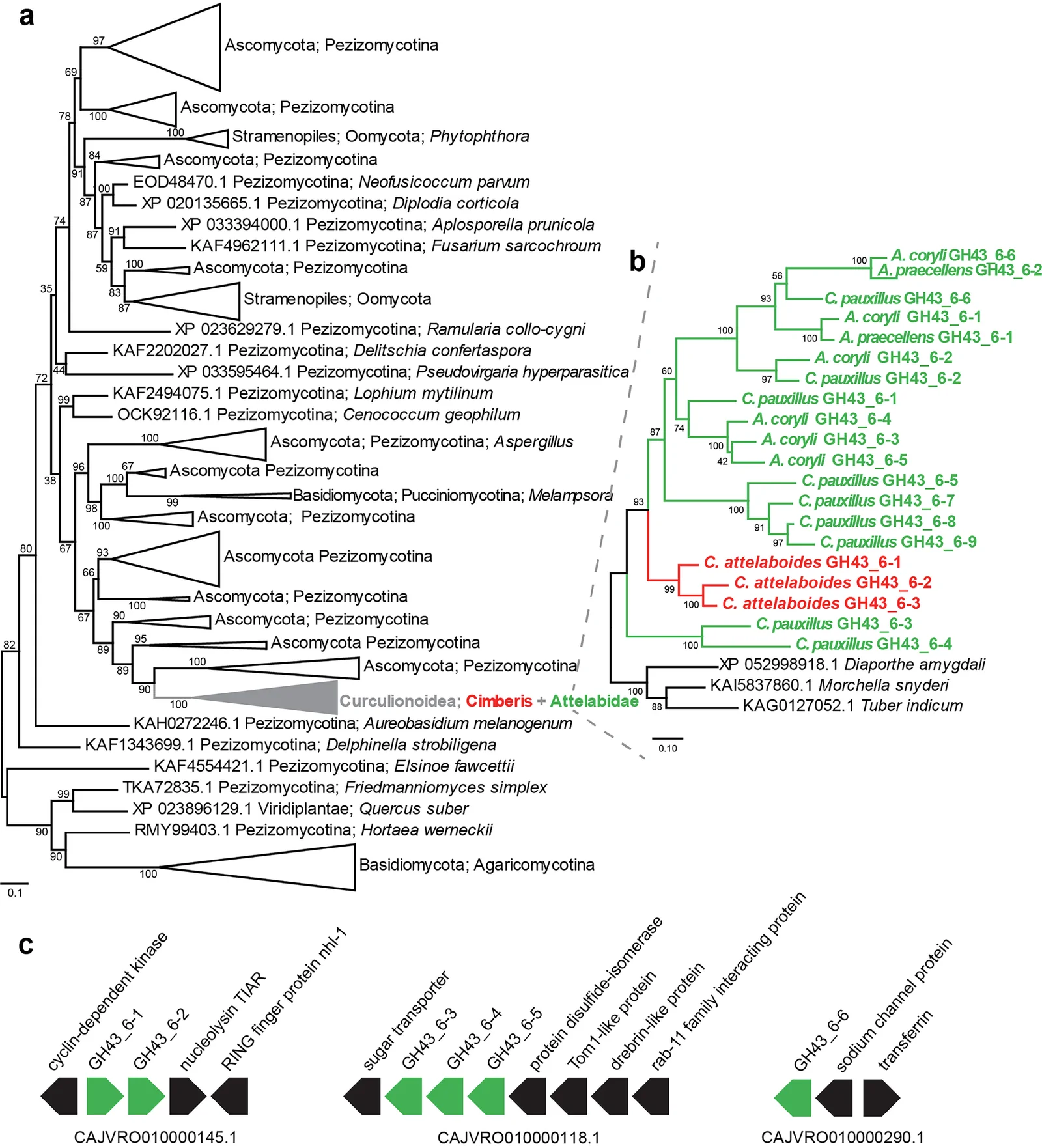
Single acquisition of GH43_6-encoding genes by HGT in two weevil families, followed by gene duplications. **a** The phylogenetic relationships of weevil-derived GH43_6 sequences (gray clade, collapsed) with their fungal and Oomycete counterparts were analyzed using maximum likelihood with 1000 UltraFast bootstrap replicates in IQ-TREE. The ‘Whelan and Goldman’ (WAG) model, incorporating a FreeRate model (+R; number of categories = 4), was determined as the best model of protein evolution. UltraFast Bootstrap values are provided next to the branches. The tree presented is rooted at the midpoint. **b** A separate phylogenetic analysis was conducted to determine the relationships among weevil-derived GH43_6 sequences alone. Maximum likelihood analysis with 1,000 UltraFast bootstrap replicates was performed using IQ-TREE. The WAG model was selected as the best model of protein evolution. This model incorporated a discrete gamma distribution (shape parameter = 4) to account for evolutionary rate differences among sites (+G) and included a proportion of invariable sites (+I). UltraFast Bootstrap values are indicated next to the branches. The tree was rooted using three fungal-derived sequences of the Pezizomycotina (indicated in black). *Cimberis*-derived sequences are highlighted in red, while Attelabidae-derived sequences are shown in green. **c** The arrangement of GH43_6 genes of *Apoderus coryli* (green) on the publicly available draft genome contigs is illustrated, including the identified flanking genes. The representation is not drawn to scale.

Transcriptome and genomic analysis using dbCAN identified a GH43_6 gene in *C. attelaboides* and three Attelabidae species (*Caenorhinus pauxillus*, *Apoderus praecellens*, and *Apoderus coryli*), marking the first discovery of this enzyme family in animals. According to the CAZY database (http://www.cazy.org/GH43_6.html^10^;), the only characterized function to date of GH43_6 in several fungal species is an endo-α-1,5-L-arabinanase activity (EC 3.2.1.99). This enzyme targets arabinan side chains of rhamnogalacturonan I (RGI), classifying it as a pectolytic enzyme^39^. Maximum Likelihood analysis revealed that weevil GH43_6 sequences formed a monophyletic group, closely related to counterparts expressed by Pezizomycotina fungi. This suggests an HGT event from a Pezizomycotina fungal donor to the most recent common ancestor of *Cimberis* and Attelabidae (Fig. 3a, b). We investigated the publicly available genome of *A. coryli* to examine the organization and structure of genes encoding GH43_6 proteins. The six GH43_6 genes encoded by the *A. coryli* genome were distributed across three scaffolds (Fig. 3c). GH43_6 genes exhibited a conserved structure, containing three to four introns with introns 1, 2, and 4 conserved across all six genes, while intron 3 was restricted to GH43_6-1, -2, and -6 (Supplementary Fig. S6b, c). Notably, some of these intron positions are also conserved in the closely related fungal homologs, further supporting a fungus-to-weevil HGT event followed by partial retention of the donor’s gene structure (Supplementary Fig. S6b).

### GH5_2: highly expanded within a single species of weevils

GH5_2 enzymes were identified in the transcriptome and genome of *A. fasciculatus*, with flanking regions showing conserved insect-specific synteny, confirming their insect origin (Supplementary Fig. S1). Phylogenetic analysis revealed that *A. fasciculatus* GH5_2 sequences cluster within a clade of Cerambycidae counterparts, which have previously been shown to form sub-clades based on specific enzymatic activities (Supplementary Fig. S7) ^40^. Notably, GH5_2-1 of *A. fasciculatus* grouped within a sub-clade containing sequences characterized as xylanases^40–42^, while the other nine GH5_2 sequences formed a clade that is related to Cerambycidae xyloglucanases, cellulases, and mannanases. Structural analysis revealed variations in GH5_2 genes: five lacked introns, resembling Cerambycidae genes^41,43^, while the other five contained a single intron in a conserved position, dividing the ORF into two exons (Supplementary Fig. S1). Their localization in two tandemly arrayed gene clusters suggests recent gene duplication events. The absence of GH5_2 genes in other weevil species implies an *A. fasciculatus*-specific acquisition (Fig. 1; Supplementary Table S1). The high sequence similarity to Cerambycidae GH5_2 (e.g., 82% identity with the GH5_2 gene derived from *Acanthocinus aedilis* (Cerambycidae; Lamiinae)), together with evidence of tandem gene duplication and distinct phylogenetic patterns, suggests a non-microbial origin. These findings support the hypothesis that *A. fasciculatus* acquired GH5_2 through a recent HGT event from an insect donor, likely a cerambycid beetle, rather than from a microbial source.

### Dietary specialization explains PCWDE repertoire across weevils

Our analyses revealed both conserved and lineage-specific patterns in PCWDEs evolution. Weevil species broadly varied in the number of encoded PCWDE families, from one in *Platyrhinus* and *Arrhenodes* to nine in *Lissorhoptrus*, as well as in the number of genes per family. Interestingly, a convergent reduction of PCWDE repertoires was observed in weevils with specialized feeding ecologies: Fungivorous (*Platyrhinus resinosus*, *Euparius marmoreus*) and sap-feeding species (*Arrhenodes minutus*) exhibited highly reduced PCWDE repertoires. Similarly, ambrosia beetles (*Oxoplatypus quadridentatus*, *E. fornicatus*, *Xyleborus glabratus*), which live in a nutritional symbiosis with fungi, showed highly reduced PCWDEs compared to their plant-feeding relatives (Fig. 1; Supplementary Table S1). To test for the impact of dietary preference on the PCWDE repertoire across weevils, we conducted principal component analyses (PCA) for both larval and adult feeding habits in 48 weevil species (Supplementary Fig. S8; Supplementary Table S4). We first compared group clustering based on primary food sources, categorized as plant-based and non-plant (fungal) for both adult and larval stages. Five species feed on fungi (*Euparius* was not included in the phylogeny, due to low BUSCO completeness) and 43 on plants during both life stages (Fig. 1; Supplementary Table S4). The first two principal components explain 42.4% of the total variance (PC1 = 23.8%, PC2 = 18.6%) (Supplementary Fig. S8). A permutational MANOVA confirmed that PCWDE gene family size patterns differed significantly between the plant- and non-plant-feeding groups (R² = 0.227, *p* = 0.001).

**Fig. 4:**
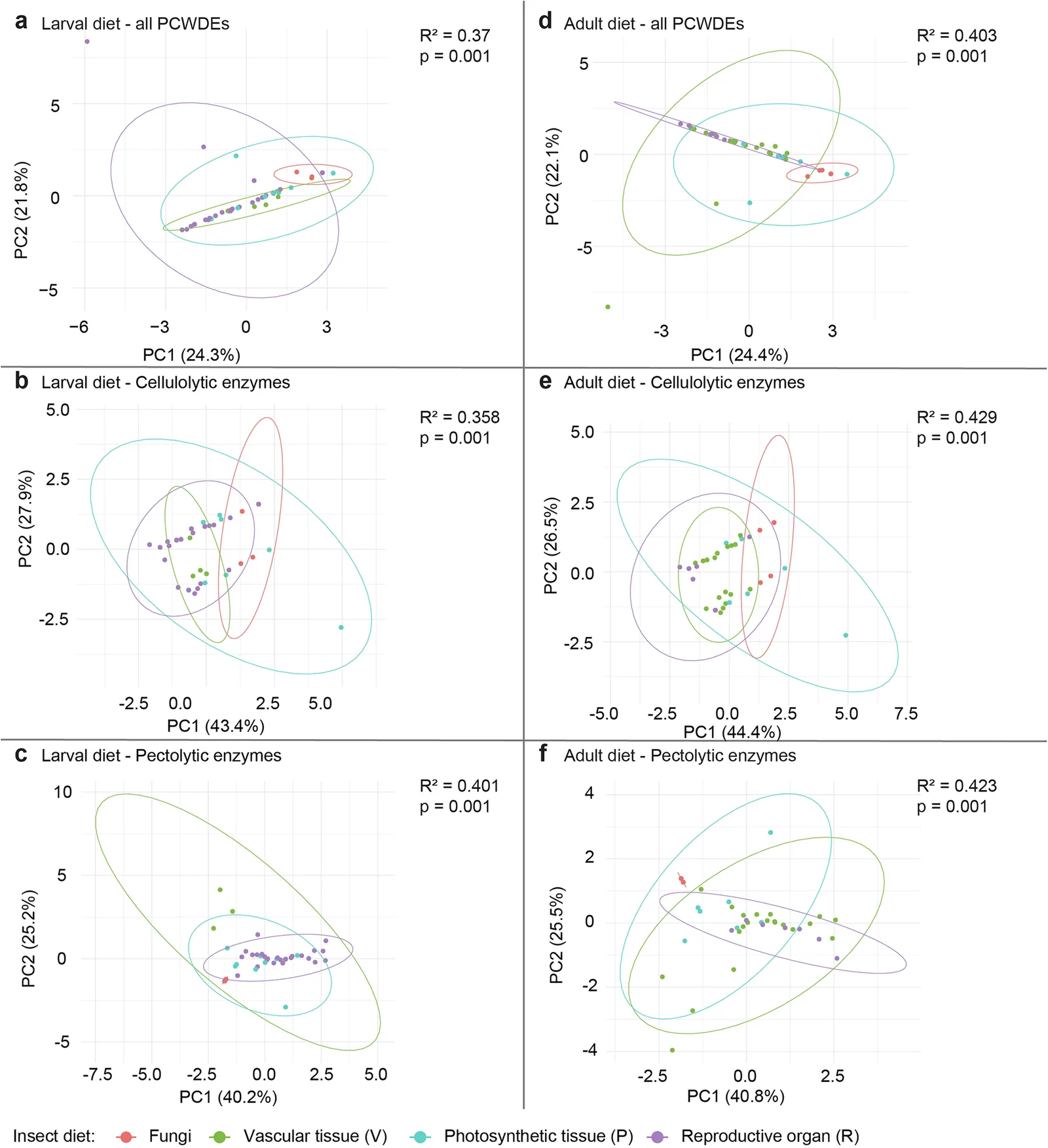
Separation of larval and adult feeding niches based on PCWDE gene family sizes. PCAs were performed with the numbers of PCWDE genes per family as displayed in Fig. 1, and species are colored by feeding niche. A total of 43 plant-feeding species and 5 fungal-feeding species were included in the PCA. For more detailed PCA analyses, species were grouped by dietary preferences: reproductive tissue feeders (strobili, pollen, flowers or seeds: R); photosynthetic tissue feeder (leaves: P); or vascular tissue feeders (stem, twig, trunk, or roots: V). Because some species shift dietary preferences between life stages, the number of species in each group differs between larvae and adults (larvae: *P* = 4, R = 9, V = 26; adults: *P* = 21, R = 7, V = 7; see Fig. 1 and Supplementary Table S4 for details). To account for these differences, clustering analyses based on this higher resolution dietary classification were conducted separately for larval (**a**–**c**) and adult stages (**d**–**f**). All analyses were validated by permutational MANOVA (two-sided test), confirming significant separation among the dietary groups.

To examine diet and gene number correlations more closely, we categorized plant feeders into three groups: Photosynthetic tissue (P group), reproductive organ (R group), and vascular tissue feeder (V group) (Fig. 1; Supplementary Table S4). Because several species shift their dietary preference between life stages, the number of species in each group differs between larvae and adults (larvae: P group = 4, R group = 9, V group = 26; adult: P group = 21, R group = 7, V group =7; see Fig. 1 and Supplementary Table S4 for details). To account for these differences, clustering analyses were conducted separately for larval and adult stages (Fig. 4; Supplementary Fig. S9). The first two principal components explained 46.1% (PC1 = 24.3%, PC2 = 21.8%) and 46.5% (PC1 = 24.4%, PC2 = 22.1%) of the variance in larvae and adults respectively (Figs. 4a, d). Permutational MANOVA confirmed significant differences in gene profiles between dietary groups in both life stages (*p* = 0.001). We further categorized gene sets into three functional groups: cellulolytic, hemicellulolytic, and pectolytic enzymes (Fig. 1), and we compared group clustering across our four dietary categories. For both life stages, the first two principal components explained between 65.4% and 71.3% of the total variance with cellulolytic (Fig. 4b, e) and pectolytic enzyme groups (Fig. 4c, f). Permutational MANOVA also confirmed significant group separation in these enzyme categories for both larvae and adults (*p* = 0.001). Thus, significant gene family size variation is also observed within plant feeders, with the specific plant source/tissue being reflected in the PCWDE repertoire. However, the hemicellulolytic enzyme group did not show clear group separation in either life stage, which was likely due to the small sample size, because only few of the weevil taxa encoded genes for hemicellulolytic enzymes (Supplementary Fig. S9).

Next, by incorporating phylogenetic relationships (Fig. 1), we evaluated whether PCWDE encoding gene numbers were attributable to shared ancestry or reflected selection pressures associated with dietary specialization. Using a phylogenetically informed MANOVA, we compared the association between dietary categories defined previously and gene numbers of PCWDEs. Interestingly, we detected a significant association between dietary differentiation and gene distribution patterns in both larvae and adults across all PCWDEs (*p* < 0.05; Table 1). When analyzing PCWDE categories separately, both larvae and adults exhibited strong associations between dietary specialization and the number of pectolytic enzyme genes (GH28, CE8, PL4, GH78, GH43_6; *p* < 0.05). In contrast, no significant association was found for cellulolytic enzymes (GH9, GH45, GH48, and GH5_2) or hemicellulolytic enzymes (GH43_26, GH10, GH5_8, GH5_10). These results suggest that the diversity of PCWDEs – particularly pectolytic genes – is closely linked to dietary specializations in weevils, and weevil species sharing the same diet converged on similar PCWDE repertoires.

**Table 1:** *P*-values from phylogenetic MANOVA tests (two-sided) assessing the association between dietary differences and gene number variation in adult and larval weevils, respectively

	All PCWDEs	Cellulolytic	Hemicellulolytic	Pectolytic
Adult	0.004*	0.116	0.529	0.05*
Larvae	0.034*	0.157	0.794	0.036*

Significant associations (*p* < 0.05) are marked with *p > 0.05 indicates no significant association.

## Discussion

Our comprehensive analysis of transcriptomes and genomes across representatives of 24 weevil subfamilies, coupled with phylogenetic reconstructions, provides interesting insights into the evolutionary relationships and the distribution of PCWDEs in Curculionoidea and Phytophaga beetles in general, thus complementing earlier studies^20,24,38,44^. Moreover, by leveraging a broader dataset and phylogenetic approaches, our findings reveal lineage-specific adaptations that underscore the role of PCWDEs in dietary specialization and ecological diversification, with adult and larval nutritional ecology being reflected in the repertoire of PCWDE-encoding genes.

### The general pattern of PCWDE distribution: setting the core into context

The distribution of PCWDE families across weevils highlights both commonalities and exceptions in enzyme repertoires. Notably, core PCWDE families, including cellulolytic GH9, GH45 and GH48, as well as pectolytic GH28, are widely present across almost all species analyzed as well as in Phytophaga in general, highlighting their important roles in plant cell wall degradation. Their essential role is further supported by silencing or knockout experiments in leaf beetles, as the substantial reduction of either cellulase (GH45, GH48) or pectinase (GH28) activity resulted in slower larval growth, longer developmental time and elevated mortality^21,23,45,46^. In contrast, hemicellulolytic enzymes exhibit a patchier distribution in weevils (this study) and leaf/longhorned beetles^24^, which may correlate with the adaptation to specific ecological niches (see next paragraph for details). Whether this pattern of widespread cellulases and pectinases but patchy hemicellulases also applies to the Buprestidae, the second largest clade of herbivorous beetles encoding an expansive set of PCWDEs, remains an open question due to very limited taxon sampling in previous studies ^20^. However, in other herbivorous insect clades cellulases and pectinases are also dominating over hemicellulases in Phasmatodea^47^ and in the gall-forming Hymenopteran family Cynipidae^17^. It seems plausible that similar food sources and feeding modes, i.e., chewing on growing plant tissues, has favored the convergent evolution of similar enzymatic machineries, dominated by cellulolytic and pectolytic enzymes to break open plant cells.

### Pectin degradation in weevils: the most comprehensive among Phytophaga

Pectin is a complex carbohydrate mostly present in primary cell walls and middle lamellae of plants^48^ and serves as the initial line of defense against microbial invaders^49^. Concordantly, pathogens deploy a variety of PCWDEs, including PGs of family GH28, CE8 pectin methylesterases, and pectin/rhamnogalacturonan lyases of family PL4, to break down pectin polysaccharides. Our analyses reveal several of these enzymes (GH28, PL4 and CE8) to be present in weevils as well, where they can occur individually or together, collectively forming the most diverse set of pectolytic enzyme families in the Phytophaga, and across insects in general.

Like in other Phytophaga clades, GH28 PGs are the most frequent pectinases found in weevils. However, their evolutionary history is much less dynamic compared with what we previously found in leaf beetles, but highly similar to the patterns observed in longhorned beetles^24,50^. Our phylogenetic analyses indicate two different fungal origins of weevil GH28s. The GH28 PGs in *A. fasciculatus* show a closer relationship to PGs derived from Saccharomycotina, while GH28 PGs found in all other weevils are more closely related to sequences from Pezizomycotina, as observed in many leaf beetles (Chrysomelidae) and most longhorned beetles (Cerambycidae)^24,35,38^. The only other beetle lineage within the Phytophaga clade with similar GH28 PGs to those in *Araecerus* is the longhorned beetle subfamily Lamiinae^35,38^. The most parsimonious explanation for this patchy distribution of Saccharomycotina-like GH28 PGs in Phytophaga is beetle-to-beetle HGT. Such intra-phylum HGTs could have been facilitated by insect viruses, serving as vehicles of genetic material in the transfer process^51^. Recent phylogenetic analysis of Anthribidae suggests *Araecerus* shifted to plant-feeding secondarily as it evolved from fungivorous ancestors, which on their part originated from an herbivorous ancestor^30^. This sequence of dietary shifts (herbivory to fungivory and back to herbivory) may have caused massive gene losses (like we see in other fungivorous weevils) followed by new HGTs, as we see in *Araecerus* GH28 but also GH5_2. The alternative scenario of an ancestral GH28 acquisition appears less likely for two reasons: First, the early acquisition of both types of fungal GH28s would have led to their coexistence for a long time in certain Phytophaga lineages to explain the pattern we observe. However, not a single beetle with coexisting GH28s of different origins has been found so far, indicating that redundancy is purged quickly^24^. Secondly, the ancestral acquisition-scenario of a Saccharomycotina GH28 implies massive numbers of losses and is thus less parsimonious. Moreover, the number of losses would be innumerable when postulating an even earlier common HGT event in insects due to the conserved intron in the Saccharomycotina-like GH28 we found in Phytophaga (*Araecerus*, Lamiinae), Buprestidae and Miridae (Supplementary Fig. S2). In contrast, beetle-to-beetle, or mite-to-beetle HGTs, like we inferred in other PCWDE families (GH5_10, GH43_26, GH78), are not only more parsimonious but also explain the conserved intron position equally well. Alternatively, multiple independent acquisitions from closely related microbial donors in mites and beetles are possible, but seem less likely, given the lack of closely related microbial sequences in the databases.

While many weevils encode PL4 and CE8 enzymes in their genomes, such enzymes are absent from longhorned beetles^38^, and rare in leaf beetles, where they are mainly provided by microbial symbionts^24^. Furthermore, both gene families are also absent from other herbivorous insect clades that solely rely on pectolytic GH28s like Miridae and Phasmatodea^52^. As the pectin in primary cell walls but not in the middle lamella is methylated^53^, the presence of CE8 likely allows for a more efficient cell wall pectin digestion in weevils compared to most other Phytophaga. In the rice weevil *Sitophilus oryzae* (Dryophtorinae), the presence of CE8s boosts the efficacy of GH28 PGs by demethylating galacturonic acid residues within homogalacturonan^36^. Our investigation suggests that these CE8s and their contribution to homogalacturonan digestion are prevalent among species within the Curculionidae family. Similar to CE8, PL4-encoding genes are prevalent in Curculionidae species, except for Dryophtorinae, and we also detected their presence in *Apion* (Brentidae) and *Bruchela* (Anthribidae). Unlike CE8, PL4_4 has a more complex evolutionary history, likely shaped by multiple HGT events followed by independent losses across various insect clades. Unfortunately, no animal-encoded PL4 has been functionally characterized so far. Thus, it remains an open question if the efficacy of pectin digestion in weevils is further enhanced by a PL4-mediated rhamnogalacturonan I breakdown as it has been shown in fungi^54^.

### The hemicellulases perspective: functional implications

The patchy distribution of hemicellulolytic enzymes we found across weevils and Phytophaga in general is astonishing, as hemicelluloses are abundant in most living plant tissues´ primary (xyloglucan) and secondary (xylan and/or galactomannan) cell walls^55^. Thus, it would seem advantageous for the majority of Phytophaga beetles to carry a set of enzymes targeting the diversity of hemicelluloses present in their diet. Four plausible scenarios may explain the patchy distribution of hemicellulases in Phytophaga.

First, these enzymes may only be adaptive occasionally when consuming diets exceptionally rich in hemicellulose. The presence of GH5_8 mannanases in *H. hampei* has been hypothesized to be an adaptation to feed on coffee berries, which are rich in galactomannan^56,57^. This hypothesis is supported by our observation of GH5_8 mannanases in *A. pilosula*, which is the only species in our dataset feeding on galactomannan-rich gymnosperm softwood^58^, and beside *H. hampei* the only known weevil encoding GH5_8 mannanases. Similarly, leaf beetles of the subfamily Bruchinae feed on galactomannan-rich legume seeds^59^ and they are the only leaf beetles encoding their own mannanases (GH5_10)^24^.

Secondly, the absence of hemicellulose backbone-cleaving enzymes might be compensated for by the acquisition of enzymes targeting hemicellulose sidechains to loosen plant cell walls. A set of debranching enzymes is known from phytopathogenic microbes able to remove hemicellulose sidechains^60^. However, only a single family, GH43_26, has been previously identified in beetles, including the jewel beetle *A. planipennis*, two leaf beetle species and several longhorned beetles^20,24,38^. Based on the enzymatic functions of characterized microbial enzymes within this subfamily, GH43_26 enzymes likely function as α-L-arabinofuranosidases (EC 3.2.1.55), which are involved in the removal of L-arabinofuranose side chains linked either through α-1,2- or α-1,3-glycosidic bonds to xylose residues in arabinoxylan backbones^61^. All but one species of the longhorned, leaf and jewel beetles possessing GH43_26 enzymes lack backbone cleaving xylanases, indicating the former debranching xylanases compensating for the absence of the latter. Here, we have identified GH43_26 genes for the first time in weevils, specifically in the transcriptomes of two Curculionidae species, *T. mucorea* (Baridinae) and *L. oryzophilus* (Brachycerinae). However, in both of these species, GH43_26 enzymes are found to co-occur with GH10 xylanases, which are responsible for breaking down the xylan backbone. This co-occurrence suggests a highly effective, synergistic xylan digestion process by these two species. Thus, depending on the beetle clade, debranching enzymes may add on the activity or compensate for the lack of backbone-cleaving hemicellulases.

Third, hemicellulases can be encoded by nutritional symbionts thus contributing to cell wall degradation. Microbial symbionts with putative hemicellulolytic capabilities were recently found in leaf beetles of the subfamilies Cassidinae, Eumolpinae and Spilopyrinae^24,62,63^. Such PCWDE-secreting bacterial symbionts are so far unknown from other Phytophaga beetles like longhorned beetles and weevils, although associations with microbes are widespread in these two lineages^64^.

And fourth, hemicellulase activity could be taken over by members of an originally non-hemicellulolytic PCWDE family. The digestive capabilities of PCWDEs in an insect´s gut cannot be accurately predicted solely based on functional annotations by assignment to CAZy families. This is of particular importance as HGT events of PCWDEs are often followed by extensive gene duplications allowing for subsequent sub- and neofunctionalizations^65^. Specifically, individual members of otherwise cellulolytic families were shown to possess hemicellulolytic activities like xyloglucan and mannan hydrolyzing GH45s in leaf beetles and in the weevil *S. oryzae* ^23^. Similarly, ancestral state reconstruction coupled with functional characterization revealed that members of GH5_2 evolved various hemicellulolytic activities in longhorned beetles ^40^. A similar process of sub-functionalization may have occurred in *Araecerus* GH5_2 enzymes, indicated by the presence of ten gene copies clustering with the functionally diversified longhorned beetle counterparts. Interestingly, GH5_2 gene family expansion correlates with a reduced set of other cellulolytic or hemicellulolytic gene families in both longhorned beetles and *Araecerus*, further indicating a possible example of functional convergence.

### The evolutionary history of PCWDEs: a complex dynamic of HGT events

**Fig. 5:**
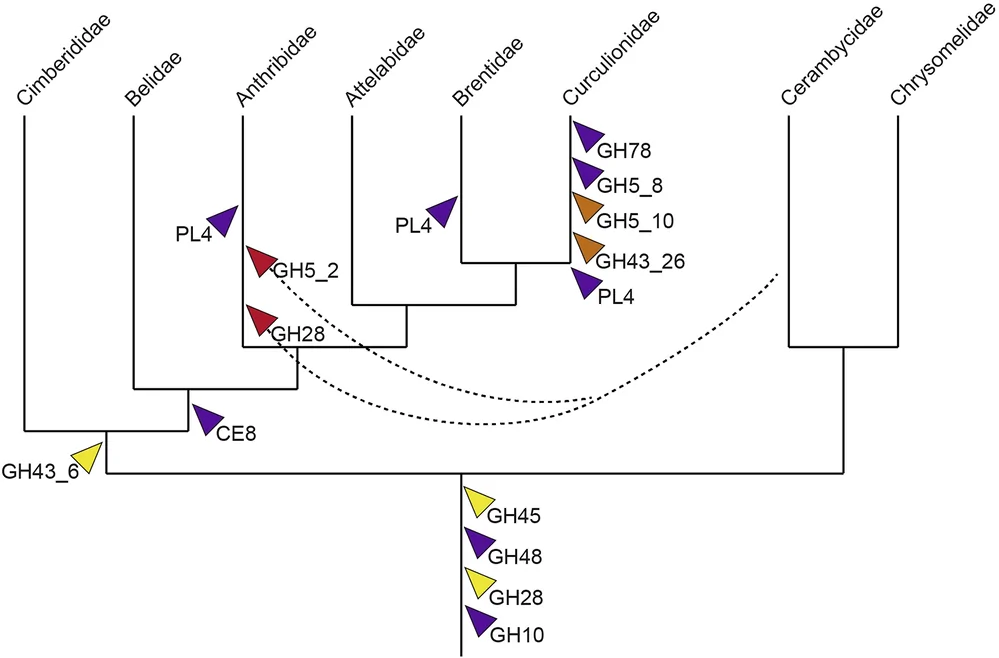
Proposed scenario of HGT events in weevils. Species phylogeny of Fig. 1 is schematically shown. Acquisition events are indicated with arrow heads. Colors indicate likely HGT origins with yellow, blue, brown and red representing fungal, bacterial, mite and beetle donors respectively (see Supplementary Table S6 for details). For simplicity, proposed gains but not losses are depicted.

The vast majority of PCWDE families in Phytophaga beetles is reported to originate from HGT events from different microbial donors (mostly bacterial). However, their specific evolutionary histories are complex and diverse, including individual or multiple independent acquisitions via HGT followed by gene duplications, sub- and neofunctionalizations, persistence, replacement or loss. Here, we found widely distributed families like pectolytic PL4 and GH28, as discussed above, that show a dynamic evolutionary history characterized by multiple independent acquisitions, replacements and losses. In contrast, cellulolytic core families were most likely acquired once in beetles (GH45, GH48) or are even of ancestral metazoan origin (GH9)^20,23,66^; (Fig. 5). At the same time, the patchily distributed Phytophaga GH10 was likely frequently lost in several Phytophaga lineages after a single acquisition event indicated by clustering in the gene tree and conserved gene structure. Such complicated evolutionary history involving frequent losses in several taxa after common acquisition was also found for animal alpha-amylases GH13_1^67^. Furthermore, we inferred from our analyses that the most parsimonious explanation for the close proximity of some beetle PCWDEs in the gene trees is intra-phylum HGT. This may not only include beetle-to-beetle HGT like in GH5_2 and Saccharomycotina-like GH28 (both between *Araecerus* and Lamiinae) but also transfer events between mites and beetles like in GH5_10 and GH43_26. However, we cannot exclude the possibility of independent acquisitions from closely related donors explaining the clustering of mite and beetle sequences. In contrast, frequent independent acquisitions, like previously postulated for GH5_8 in Phytophaga but also the aforementioned GH28s, PL4 as well as the GH5_10 on a larger scale of Arthropoda, were described for animal GH32 fructanases, indicated by similarities to different bacterial orders as potential donors^68^.

### Dietary shifts and specialized herbivory explain dynamic PCWDE evolution in weevils

Gene family expansions can provide the basis for sub- and neofunctionalization and thereby enable evolutionary adaptations, as postulated for genes involved in detoxification of plant secondary metabolites in herbivorous insects^69^. Previous characterizations of PCWDEs’ activities in Phytophaga indeed revealed functional diversification ^23,35,40^, but the connection to the beetles’ nutritional ecology remained elusive. Our phylogenetically informed analyses of gene number variation provide the first statistical support for the adaptive expansion or contraction of PCWDE gene families across species. We verify that gene family sizes differ between fungus and plant feeders, with different fungivorous lineages independently converging on a reduced set of PCWDE families and genes per family. However, this finding should be interpreted with caution due to the small sample size of fungivorous species in our analysis and therefore requires validation using a larger dataset. In addition, we detected significant differences across herbivorous weevils by comparing species feeding on vascular, photosynthetic or reproductive tissues. Thus, our findings not only reveal that the acquisition of PCWDEs by HGTs repeatedly promoted the evolution of specialized herbivory in weevils, but also show that lineage-specific gene family expansions and contractions reflect the dynamic and adaptive evolution of PCWDE repertoires in relation to dietary shifts.

### Limitation of the study

A limitation of this study is that it relies predominantly on transcriptome data rather than complete genome assemblies. Consequently, estimates of gene copy number may not fully capture the entire genomic repertoire, as both underestimation (owing to genes not expressed under the sampled conditions) and overestimation (resulting from assembly artifacts or transcript redundancy) remain possible. To minimize such biases, all datasets were processed using an identical assembly and annotation pipeline, and each PCWDE candidate was subjected to manual curation, thereby reducing methodological inconsistencies among species. We therefore expect any residual false-positive or false-negative rates to be broadly comparable across assemblies. Furthermore, PCWDEs are typically expressed at moderate to high levels, which likely reduces the risk of their underrepresentation in transcriptome-based datasets. Nevertheless, the availability of additional high-quality, chromosome-level Phytophaga genomes will enable further refinement and validation of the gene repertoires reported here.

## Conclusion

Based on our findings, we conclude that weevils collectively possess at least 13 CAZyme families/subfamilies involved in plant cell wall degradation. Our study highlights the diverse and adaptable repertoire of PCWDEs in herbivorous Curculionoidea, facilitating the breakdown of plant cell wall polysaccharides. Conversely, weevils that have transitioned to feeding on fungi or plant sap have either lost or significantly reduced their complement of these enzymes. Interestingly, the set of PCWDEs reflect larval as well as adult diet when correcting for phylogeny, revealing the importance of PCWDEs for nutrition in both life stages. Our study provides novel insights into the evolutionary dynamics of PCWDEs in Phytophaga, highlighting the roles of HGT events and gene duplications in shaping enzyme repertoires ultimately allowing for dietary specializations and adaptive radiations. Additionally, our work provides a scenario of HGT events leading to the repertoire of PCWDEs in the Phytophagan ancestor (Fig. 5), which likely contributed to the enormous evolutionary success of this megadiverse group of insects and laid the foundation for one of the most successful adaptive radiations on earth.

## Methods

### Insect collection

Beetle specimens were collected from 2010 to 2022. The majority of specimens were sent to the Max Planck Institute for Chemical Ecology (Jena, Germany). To ensure RNA preservation, specimens were submersed and stored in RNAlater solution (Thermo Fisher Scientific, Darmstadt, Germany). Upon receipt, each beetle specimen was extracted from the RNAlater solution, transferred to a new microcentrifuge tube, and immediately frozen at −80 °C until further analysis. For specimens collected in the vicinity of Jena, beetles were captured alive and transported to the laboratory where they were shock frozen at −80 °C.

Except for two weevil families (Caridae, Nemonychidae) and 4 well established subfamilies (Rhinorhynchinae, Nemonycinae, Conoderinae *sensu strictu* and Mesoptiliinae) all major clades were covered by our sampling. The four smaller groups with unresolved phylogenetic position and relationships in Brentidae, the Eurhynchinae, Ithycerinae, Microcerinae and Nanophyinae were not taken into account for subfamily analysis.

### RNA extraction and sequencing

Whole beetle specimens were utilized for total RNA extraction employing Trizol reagent (Thermo Fisher Scientific) following the manufacturer’s guidelines. To eliminate potential genomic DNA contamination, samples underwent DNase treatment (TURBO DNase, Thermo Fisher Scientific) at 37 °C for 30 min. Subsequently, RNA samples were subjected to purification using the RNeasy MinElute Clean up Kit (Qiagen, Hilden, Germany) according to the manufacturer’s instructions. The purified RNA was eluted in 20 μl of RNA storage solution (Thermo Fisher Scientific, MA, USA). The integrity and quality of RNA samples were evaluated using the RNA 6000 Nano LabChip kit on an Agilent 2100 Bioanalyzer (both from Agilent Technologies, Santa Clara, CA, USA). RNA sequencing was performed by the Max-Planck-Genome Center (Cologne, Germany). Prior to library preparation, Poly(A) + -RNA was enriched, followed by fragmentation to an average size of 300–350 nucleotides. Paired-end sequencing libraries (2×150 bp) were constructed using the TruSeq Stranded mRNA Library Prep kit (Illumina, San Diego, CA, USA) incorporating double-indexed adapter tags. Sequencing was carried out on an Illumina HiSeq 3000 platform with a target of 60 million paired-end reads per library.

### RNA-Seq data assembly

Sequence assembly of the paired-end reads was conducted using CLC Genomics Workbench v21.0 (Qiagen). First, sequences underwent trimming for length and quality employing standard settings. Then, assembly was performed using the following CLC parameters: nucleotide mismatch cost = 2; insertion = deletion costs = 2; length fraction = 0.3; similarity = 0.9. In cases of conflicts among individual bases, resolution was achieved by voting for the base with the highest frequency. Contigs shorter than 200 bp were excluded from the final analysis. It is known that Illumina read coverage directly shapes transcriptome assembly. While low coverage leads to fragmented, incomplete transcripts, excessive coverage creates its own issues, increasing false-positive splice variants and artifactual chimeric fusion transcripts. As genes are differentially expressed, one specific read proportion used for de-novo transcriptome assembly might be optimal for many but not all genes. To assess the impact the number of reads can have on assembly quality, multiple assemblies were generated using subsets of the total reads randomly selected. Specifically, the first assembly incorporated 6 million reads, followed by subsequent assemblies incorporating 25%, 50%, 75%, and finally all reads. To evaluate transcriptome completeness, the Benchmarking Universal Single-Copy Orthologs (BUSCO) analysis^70^ was performed using the BUSCO v5.2.1 pipeline and compared against the insect_odb10 database, which contains 1658 single-copy orthologous genes specific to insects.

### Recovery and analysis of publicly available RNA-Seq and genome

In addition to our generated transcriptomes, we increased their number by retrieving RNA-Seq datasets sourced from the Short Read Archive (SRA) or the transcriptome shotgun assembly (TSA) databases at NCBI for species within the Curculionoidea superfamily. Selection criteria were based on information extracted from the Bioproject records accompanying each dataset. Specifically, only datasets derived from RNA isolated from entire beetles or larvae, as well as from dissected midguts, were included. Conversely, datasets utilizing RNA from tissues other than the midgut (such as antennae or legs) were excluded from our analysis. For datasets retrieved from SRA, we performed transcriptome assembly using the same procedure described above. This was not necessary for the datasets obtained from TSA as they were already assembled transcriptomes. Similar to our internally generated datasets, we evaluated the completeness of these additional transcriptomes through BUSCO analysis. Comprehensive details of all transcriptomes and genomes included into our analysis are summarized in Supplementary Tables S1, S3.

### Sequencing and assembly of the *Araecerus fasciculatus* draft genome

Genomic DNA (gDNA) extraction was performed on six beetles sourced from a laboratory culture maintained at the Julius Kühn Institute for Ecological Chemistry, Plant Analysis, and Stored Product Protection (Berlin, Germany). Insect tissue was homogenized in 500 µl of buffer CT (Circulomics, Baltimore, MD, USA) in a 2-ml microcentrifuge tube by subjecting it to mechanical disruption using a pestle. Subsequently, homogenization was achieved using a Sunlab Rotator “Digi Mixer” (NeoLab, Heidelberg, Germany). The isolation of gDNA was carried out with the Nanobind Big DNA kit (Circulomics), followed by the isolation of high molecular weight (HMW) DNA fragments using the Short Read Eliminator kit XS (Circulomics), following the manufacturer’s protocols. HMW DNA underwent further purification using AMPure XP beads (Beckman Coulter, Krefeld, Germany) as per the manufacturer’s instructions. The purity and concentration of the final DNA samples were determined using Nanodrop (Thermo Fisher, Waltham, MA, USA) and Qubit (Thermo Fisher) instruments.

Prior to library preparation, end-DNA repair was conducted using the NEBNext Ultra II DNA Library Prep Kit (New England Biolabs, Ipswich, MA, USA) following the manufacturer’s instructions. Sequencing adapters were then ligated using the Ligation Sequencing Kit (Oxford Nanopore Technologies, Oxford, UK), with ligation reactions incubated at room temperature for 10 min, followed by a cleanup step utilizing AMPure XP beads (Beckman Coulter). Sequencing was carried out on a MinION platform (Oxford Nanopore Technologies). Priming of the MinION flow cell for sequencing was performed using the Flow Cell Priming Kit (Oxford Nanopore Technologies). During a total run time of 72 h, flow cells were loaded with 100–200 ng of the libraries three times, with intermittent washing steps between each loading cycle facilitated by the Flow Cell Wash Kit EXP-WSH003 (Oxford Nanopore Technologies).

Following sequencing, raw reads underwent high-accuracy base calling using an optimized version of Guppy v6.0.1 (Oxford Nanopore Technologies) employing the dna_r9.4.1_450bps_hac.cfg model. The resulting reads were subjected to de novo assembly utilizing Flye v2.7.1^71^, with a minimum overlap set to 10 kb and utilizing the “--meta” option. Subsequently, the Flye assembly underwent four rounds of polishing using Racon v1.3.3^72^ with the following parameters: -m 8 -x -6 -g -8 -w 500. After each polishing iteration, reads were realigned to the resulting assembly using minimap2 v2.17^73^. A final polishing step was conducted using Medaka v1.2.0 (https://github.com/nanoporetech/medaka) employing the r941_min_high_g344 model and utilizing MinION raw reads. For final error correction, ntEdit was used with ~25 Gb paired-end (2 × 150-bp) Illumina data generated from *A. fasciculatus* HMW DNA. Following polishing and error correction, haplotype redundancies were merged utilizing Purge haplotigs v1.0.4^74^, and duplicated haplotigs were collapsed using Haplomerger2 v2.01^75^. The relative contig coverage, GC content and contig taxonomic classification were scanned after genome assembly using BlobTools and Taxon Analysis^76^ to enable the identification and removal of potential microbial symbionts or contaminants. Estimation of genome size was performed using backmap.pl v0.5^77^ based on coverage information generated from minimap2, employing default settings. GC content analysis was conducted using the seqkit tool^78^ with the fx2tab option. Lastly, the completeness of each genome was assessed using BUSCO v5.2.1, utilizing the insect_odb10 database (summary of assembly statistics: Supplementary Table S5). The resulting draft genome assembly can be found as Supplementary data 1.

### Species phylogeny

To construct a phylogenetic tree encompassing all weevil datasets compiled, we employed publicly available transcriptome datasets of Chrysomeloidea species as an outgroup. A custom pipeline was developed to infer a phylogenetic tree, utilizing a nucleotide (codon-based) alignment of orthologous genes^79^ from both transcriptome and genome data. Initially, the longest open reading frame for each transcript was predicted using transDecoder (https://github.com/TransDecoder/TransDecoder/wiki). Subsequently, a BUSCO analysis was conducted for each dataset utilizing the insecta_odb10 database, identifying orthologous genes shared across all taxa. The corresponding amino acid (AA) and nucleotide (NT) sequences of these genes were extracted. The amino acid sequences were aligned using MAFFT v7.402^80^ with automatic options in a Linux environment. The resulting alignment was back-translated into the corresponding nucleotide alignment using pal2nal.pl^81^, with gap trimming performed using trimAl 1.2rev59, accepting 5% gaps for each position^82^. Additionally, CIAlign was utilized to remove overly diverged gene sequences potentially caused by misannotation or incorrect prediction of open reading frames^83^. Following concatenation of aligned nucleotide sequences for each taxon, a maximum likelihood (ML) phylogenetic analysis was conducted using IQ-TREE 2.0.3^84^. The best-fit substitution model was determined within IQ-TREE using ModelFinder^85^. Nodal support statistics were estimated using ultrafast bootstrap analysis^86^ implemented in the IQ-TREE software with 1000 replicates (-bb 1000), SH-aLRT branch test support (-alrt 100), and branch length optimization using the BNNI algorithm (-bnni) to improve tree topology accuracy. Additional information can be found in Supplementary Data 2.

### CAZyme identification and curation of PCWDE-encoding genes

The identification of carbohydrate-active enzymes (CAZymes) was conducted using dbCAN v2.0.11^87^ via a webserver (https://bcb.unl.edu/dbCAN2/index.php). Putative genes encoding PCWDEs were identified by conducting tBLASTn searches of the transcriptomes against amino acid sequences of PCWDEs previously documented in Phytophaga species, with an E-value cutoff of 10-5^20,38,44^. Additionally, CAZyme families were screened based on their predicted function to assess the presence of novel PCWDE families not previously reported in insects. More precisely, CAZymes were classified into cellulases, pectinases and hemicellulases according to the functional data linked with each gene family on the CAZY webpage (https://www.cazy.org/). Corresponding contigs were retrieved and manually curated. For species for which draft genomes were available, genes encoding putative PCWDEs were manually inspected. The intron-exon structure of these genes was determined by comparing the cDNA sequence (when available: *Araecerus fasciculatus* and *Apoderus coryli*; in the latter case cDNA sequences from *Apoderus praecellens* were used) to the corresponding gene sequence in the genome assembly using splign^88^. This analysis aimed at identifying additional genes within each gene family that were potentially not present in the transcriptomes or missing parts of the coding sequence. For these newly discovered/completed genes, their intron-exon structure was determined using GeneWise^89^ followed by splign, relying on the most similar cDNA sequence.

To identify potential contaminants i.e. *PCWDE* sequences found in the transcriptomes but originating from insect-associated bacteria, fungi or protists, we conducted taxonomic classification of our transcriptome assemblies by kraken2 (version 2.1.3) using exact k-mer matches. Kraken2 implemented in Omicsbox (version 3.5.3) was used with confidence filter set 0.4, minimum hit groups set 2 and the reference database RefSeq 2024-11 WGS to detect “outgroup contamination”. Contigs classified as either bacteria or eukaryotes were extracted and saved as a database to run tBLASTn and BLASTn using weevil PCWDEs as queries. No hits indicating contamination were found in any of the assembled transcriptomes.

### Amino acid sequence alignments and phylogenetic analyses

Amino acid sequences corresponding to families of PCWDEs curated from our transcriptomes and their counterparts from other animal and microbes extracted from BLASTp against the NCBI nr database were recovered. In detail, beetle PCWDEs were used as queries in BLAST searches with relaxed constraints to obtain the top-100 hits. In case of the sequences extracted from NCBI, redundancy at 90% identity level from each sequence dataset was removed using CD-HIT^90^ installed on the European Galaxy server (https://usegalaxy.eu/)^91^. We note that clustering sequences at 90% identity may reduce representation of closely related taxa and could affect fine-scale inference of HGT donor lineages. However, this approach was adopted to minimize redundancy and computational burden while preserving the broader phylogenetic signal relevant to our study objectives. Moreover, based on previous findings PCWDE gene transfers did not happen recently, which makes it extremely unlikely that a donor sequence shares >90% identity with the beetle counterparts and is not included in the phylogenetic analyses because of that. Furthermore, the 90% identity threshold only applies to the backbone phylogenies (sequences taken from ncbi) and not to sequences identified in our datasets. Thus, there was no chance to eliminate publicly available sequences that were very similar to the ones we sequenced ourselves. Sequences were aligned with MAFFT (v.1.3.7) using default settings^80^. Maximum likelihood phylogenetic analyses were inferred using IQ-TREE^84^. The best-fit substitution model for each dataset was automatically determined using ModelFinder^85^ implemented in IQ-TREE and is indicated for each analysis in the corresponding figure legend. Branch supports were assessed using an ultrafast bootstrap approximation (1000 replicates)^86^. For more details, please refer to Supplementary data 3.

### Correlation of PCWDE gene family sizes with larval and adult diets

We compiled a gene number matrix for 48 weevils, covering PCWDE encoding genes. We categorized gene sets into three groups: cellulolytic enzymes, hemicellulolytic enzymes, and pectolytic enzymes. All gene number profiles were manually curated from genome/transcriptome data. Each species was assigned to feeding groups based on ecological literature for larval and adult stages. Feeding habits were assigned to each species from the plant perspective as follows. Feeding on reproductive tissues (strobili, pollen, flowers or seeds: R); feeding on photosynthetic tissue (leaves: P); or feeding on vascular tissue (stem, twig, trunk, or roots: V). Additionally, fungal feeding (fungi: F) was included as a “non-plant based” diet. The majority of data was taken from ref. ^92^.

To analyze the relationships between food sources and PCWDE encoding gene number profiles, a Principal Component Analysis (PCA) was performed using the prcomp() function using R (v 4.2.3). The gene numbers for 48 species were normalized by z-score normalization. The first two principal components were visualized with ggplot2. To test whether gene number significantly differed among dietary groups, a Procrustes distance-based multivariate analysis of variance (MANOVA) was conducted using procD.lm() function from the geomorph package with 999 permutations without phylogeny implement. The response matrix consisted of normalized gene number values, and the predictor was a categorical variable significance. Permutation-based *p* values were used to assess statistical significance. To further assess differences in gene number patterns among dietary groups while accounting with phylogenetic correction, we applied a phylogenetically informed multivariate model using procD.pgls() function from the geomorph package^93^, with 999 permutations to test for significance.

## Supplementary information

Transparent Peer Review file

Supplementary Information

Description of Additional Supplementary Files

Supplementary Data 1-4

## Data Availability

Raw reads corresponding to the newly generated transcriptomes as well as the draft genome of *Araecerus fasciculatus* have been submitted to Genbank under Bioprojects PRJNA983531 and PRJEB75246, respectively. Supplementary Tables (Supplementary Tables S1–S6) have been submitted with the present paper and are provided as Supplementary Data 1. The following supplementary data are freely accessible on EDMOND - the Open Research Data Repository of the Max Planck Society with https://doi.org/10.17617/3.5MTAYX: Supplementary Data 2 contains the draft genome assembly (unpolished and polished with final error correction) of *A. fasciculatus*. Supplementary Data 3 contains the codon-based nucleotide alignment (FASTA file), the IQ-TREE log file and the resulting tree file (Newick file) used to generate Fig. 1. Supplementary Data 4 contains the amino acid alignments (FASTA file) and the resulting tree files (Newick format) of all the phylogenetic analyses of PCWDE families presented in the paper.
