# Supplementary material for: Evolutionary dynamics of plant cell wall-degrading enzymes reflects feeding ecology in weevils
Source: Commun Biol. 2026 Sep 23;9:1243. doi: 10.1038/s42003-026-10927-0 (PMC13601563; doi:10.1038/s42003-026-10927-0)
Supplement: Supplementary file 1 [file 42003_2026_10927_MOESM1_ESM.pdf]

nature portfolio

Peer Review File

Evolutionary dynamics of plant cell wall-degrading enzymes reflects feeding ecology in weevils

Corresponding Author: Dr Roy Kirsch

This file contains all reviewer reports in order by version, followed by all author rebuttals in order by version.

Version 0:

Reviewer comments:

Reviewer #1

(Remarks to the Author)
This study utilized transcriptomes and genomes to explore the evolutionary dynamics of PCWDEs in weevils, most of which are horizontally transferred genes, and aimed to fill the gaps in our understanding of these genes in this large group of herbivorous beetles. The authors identified evolutionary patterns across 13 PCWDE families in weevils and found correlations between PCWDE repertoires and dietary specialization. This topic is compelling as it reveals the insight of correlation between special evolutionary events (e.g. HGT) and the ecological success of a taxa group.

Based on my experience, the final conclusion of the manuscript—the correlations between PCWDE repertoires and dietary specialization in beetles, particularly the reduction of PCWDEs in non-herbivorous beetles—appears reasonable and expected. However, I have three major concerns regarding this study, which may reduce its convincing power. To address these concerns, the authors should enhance their analyses to mitigate potential issues, or make the descriptions and discussions more rigorous and well-supported, providing necessary explanations and definitions within the manuscript.

1. The authors used transcriptomes to represent most species and even entire lineages. However, gene counts from transcriptomes often differ significantly from the true numbers in the genomes. Compared to genomes, transcriptomes face several challenges in providing accurate gene numbers and evolutionary patterns:
Redundant Gene Copies: Transcriptomes often contain multiple transcripts of the same gene, and misassembled transcripts, leading to gene counts that differ from the actual numbers and is positively correlated with the sequencing data size. Even after removing redundancy based on sequence similarity, these discrepancies remain, potentially compromising the correlation analysis between PCWDE repertoires and dietary specialization.
Missing Genes: Genes that are not expressed or have low expression under specific conditions may be absent from transcriptomes. While transcriptomes generally capture most genes present in genomes and offer a broad overview of gene presence, missing genes may be erroneously interpreted as gene losses, even though the genes may still exist in the genome.
Outgroup Contamination: Transcript sequences are often short and lack flanking genes that would help verify whether contamination from outgroup species, particularly symbionts in the beetle gut, is present. Excluding the possibility of such contamination is critical when detecting HGT, but this is challenging to address using transcriptomes alone.
Cross-contamination: Transcriptomes are also susceptible to cross-contamination, where sequences from other species in the same sequencing batch may be incorporated into the transcriptome, distorting the reconstruction of HGT gene evolutionary history.
Overall, I disagree with treating transcriptome data with the same weight as genome data in the analyses. I believe genome data should be considered primary, with transcriptome data serving as supplementary.

2. The authors sequenced and assembled a new genome of Araecerus fasciculatus. However, this genome appears to have been assembled solely from Nanopore reads, without subsequent polishing using Illumina reads. The accuracy of a genome assembled from Nanopore reads may not be sufficient, which could explain why the genes from this species appear unusual and occasionally cluster with genes (e.g., GH28, GH5_2) from species in other lineages.

3. The authors frequently propose intra-phylum HGT in the manuscript, which I find somewhat arbitrary and insufficiently supported. It is not uncommon for HGT genes from distant lineages to cluster together in a clade. Similar sequence compositions and conserved intron sites may also be the result of convergent evolution of these HGT genes under selective

pressures in insect genomes. And the systematic errors in gene phylogenetic reconstruction should be considered. Unless more robust supporting evidence is presented, I suggest that the concept of intra-phylum HGT should be treated as a possibility and discussed more conservatively, even if the authors lean toward this hypothesis.

Other comments:

Line 54-59: The authors describe PCWDEs derived from bacterial symbionts or the insects' own genomes. I agree with the categorization of PCWDEs into these two sources (although PCWDEs may also be secreted by symbiotic fungi or protists, in addition to bacteria). However, from the perspective of a specific insect, these two types of PCWDEs are not always distinct. For instance, most insects possess GH1 and GH9 genes (which are not derived from HGT) in their genomes, which can also aid in the digestion of plant cell wall components. Furthermore, most herbivorous insects harbor symbionts that produce PCWDEs. Therefore, I suggest refining these sentences to make them more precise, focusing on the two sources (symbionts and insects themselves) rather than on insect lineages.

Line 85-87: I would like to know how many dietary types have been included in previous studies. Since this article primarily addresses the feeding ecology of weevils, improving the representation of diets is important. I note that McKenna et al. (2019) examined about half of the weevil subfamilies, suggesting that further improvements in subfamily representation in this study may not be particularly significant. Therefore, if the enhancement of diet representation is substantial, I recommend that the authors mention it here.

Line 123: For analysis?

Line 149: Please provide the rationale or reference for selecting the midgut.

Line 233-234: I am somewhat confused by this analysis. Does it use cDNA sequences from this species to align with the genome sequences? If so, it would not detect newly discovered genes or genes absent from the transcriptomes. Or does it use cDNA sequences from other species to align with the genomes? The method description should be clarified for better understanding.

Line 237: The method or criteria used to determine HGT donors should be provided.

Line 240: Please provide the rationale for removing redundancy at the 90% identity level.

Line 249: A two-factor MANOVA could be used to assess the impact of both adult and larval diets, if feasible.

Line 251: Since PCWDEs is not mentioned for the first time, the full term should not be provided here.

Line 276-277: According to the data mentioned in Line 94 (only 2-4 subfamilies are absent in this study), do these two absent families consist of only one subfamily (or even a single genus/species) in each case? I suggest mentioning this or describing the absence at the subfamily level.

Line 520: GH9 is missing. While I understand that GH9 is not the focus of this study's HGT analysis, it is shown in the results and should at least be briefly discussed.

Line 526-537: Regarding the patchy distribution of HGT hemicellulases in independent insect lineages, one possibility is that the mechanisms for hemicellulose digestion are already present in most insects (e.g., via GH1 or symbionts).

Reviewer #2

(Remarks to the Author)
Comments to Authors.
This manuscript presents a comprehensive analysis of the distribution and evolution of plant cell wall-degrading enzymes (PCWDEs) across 24 representative subfamilies of the superfamily Curculionoidea, based on both transcriptomic and genomic data. The study provides new insights into the relationships between lineage-specific gene family expansions or contractions and feeding ecology of this group. The dataset is extensive, and the work represents a valuable contribution to understanding the molecular evolution underlying dietary adaptation in beetles. However, several aspects of the phylogenetic interpretation, the definition and categorization of feeding types, and the treatment of phylogenetic non-independence remain somewhat unclear. Further clarification and elaboration on these points would strengthen the reliability of the conclusions.

Major comments:
1. Classification of feeding categories
The authors categorize feeding habits into four groups based on the plant tissues utilized: reproductive organs (R), photosynthetic organs (P), vascular tissues (V), and non-plant-based fungal feeding (F). However, larvae of many weevils and longhorn beetle species feed on decaying wood (mixture of wood and fungal mycelia). The treatment of such "xylomycetophagous" feeders is not clearly described. It should be clarified which category these species were assigned to, and the potential impact of omitting the intermediate category on the results—especially the correlation analyses between feeding type and gene family size—should be discussed.

2. Phylogenetic inference
Although this study includes a large number of loci and a wide taxonomic range, the differences between the resulting phylogeny and previous works (e.g., Shin et al. 2018) are not sufficiently discussed. For example, the relationships among families outside Curculionidae, such as Belidae, are not mentioned. Furthermore, for branches with low support values, it should be clarified to what extent these reflect technical issues. The potential implications of the obtained topology for the higher-level classification of Curculionoidea could also be briefly discussed.
3. Feeding habits within lineages
It is unclear to what extent the sampled taxa represent the ecological diversity of their respective subfamilies or lower groups. This could affect the inferred relationships between feeding type and PCWDE gene family size. This is particularly important when considering lineage-specific patterns of gene family dynamics in relation to evolutionary time (divergence age). For example, Hypothenemus hampei is classified as feeding on reproductive tissues (R), but many congeners are phloeophagous (feeding on inner bark) or myelophagous (feeding in twig pith), suggesting a relatively recent dietary shift. In contrast, groups such as Platyopodinae appear to have maintained fungal feeding since the common ancestor of the subfamily. Such evolutionary and sampling contexts should be clarified to aid interpretation.
4. Statistical support for the reduction of gene family size in fungivorous taxa
Although the reduction in PCWDE gene numbers among fungivorous lineages appears reasonable result, the small sample size and the issue of phylogenetic non-independence call for a cautious interpretation. The authors applied a phylogenetically corrected multivariate analysis using procD.pgls(), but the statistically significant relationship seems to be limited to the overall correlation between feeding type and total gene number. This should be explicitly stated, and the reduction in fungivorous taxa should not be overemphasized if it lacks robust statistical support.

Minor comments:
L67: Kirsch et al., 2025 did not investigate longhorn beetles (Cerambycidae). Is this a miscitation or omission?
L324: There seem to be three rather than four events; two Brentinae species lost the gene but form a single clade, so these losses are not independent.
L462: Euparius marmoreus is not shown in the phylogenetic tree.
L470: The text states “five species feed on fungi,” but only four are shown in the phylogeny.
L550: Same issue as L67.

Version 1:

Reviewer comments:

Reviewer #1

(Remarks to the Author)
The authors have made substantial improvements in response to the previous round of reviews. They have addressed most of my concerns, thereby strengthening the manuscript. I particularly appreciate the benchmarks and checks conducted by the authors to demonstrate the relative reliability of their transcriptomic data. However, the authors also acknowledge that the potential risk of misestimated gene numbers (e.g., redundancy) in the transcriptomes persists. For stringency, I suggest that the authors mention this in the discussion section.

There are a few other issues in the revised manuscript:

Lines 139-142: The step involving the assembly of different read proportions appears abruptly in the manuscript. From the reader's perspective, there is either no corresponding result of the impact assessment, or no description of the downstream purpose of this step, which may be to manually curate the CAZymes, as mentioned in the response. I recommend adding downstream content of this step within the manuscript.

Line 244: "pcwde" should be capitalized.

Reviewer #3

(Remarks to the Author)
This study addresses a highly interesting and important research topic, as it may help explain why phytophagous beetles, particularly plant-feeding lineages such as weevils, have achieved remarkable species diversity. I first examined the revised manuscript and then carefully reviewed the previous reviewers’ comments together with the authors’ responses. While the authors have made substantial efforts to address earlier concerns, I would like to provide several additional comments.
My primary concern relates to the reliability of the identified genes, which overlaps considerably with issues raised by previous reviewers. Most of the PCWDE genes analyzed in this study were derived from transcriptome assemblies. Consequently, assembly errors, insufficient sequence depth, microbial contamination, and cross-sample contamination during sequencing remain potential sources of bias. If these issues are not adequately addressed, some of the inferred evolutionary patterns of PCWDEs, including horizontal gene transfer (HGT) events, may be misinterpreted. My specific comments are as follows:

1. As far as I know, sequencing based on the MinION platform is associated with relatively high error rates. Although several polishing and error-correction approaches are available, the authors should explain why this platform was chosen instead of

alternatives such as PromethION or PacBio, and provide evidence demonstrating that the resulting genome assembly is sufficiently accurate and complete for reliable target-gene identification and evolutionary analyses.
2. Please clearly describe the contents of Supporting Data 2.
3. The authors attempted to evaluate potential contamination from bacteria, fungi, and protozoa, as discussed in their response to Reviewer 1. However, potential cross-contamination among RNA-seq samples is also an important concern because it may directly affect the detection and interpretation of candidate HGT genes. The authors stated that they manually checked for identical sequences and examined COI sequences, but these approaches alone do not adequately quantify or exclude potential cross-contamination across the entire dataset. Additional analyses using software such as CROCO, together with independent validation methods (e.g., PCR amplification in additional individuals of the same species), would substantially strengthen confidence in the results.
4. Were any methods applied to treat or filter alignment gaps in the PCWDE protein sequence alignments? Please clarify.
5. Please note that removing redundant sequences at a 90% identity threshold may influence the inference of HGT donor lineages. A preferable approach would be to retain all closely related sequences in phylogenetic analyses while reducing only highly redundant representatives from the same taxonomic groups and with nearly identical sequences.
6. What criteria were used to classify genes into the three categories: cellulolytic enzymes, hemicellulolytic enzymes, and pectolytic enzymes? Please provide the classification standard.
7. It would be helpful to provide the feeding habits of each species together with supporting references. Because these data form an important basis for downstream analyses, potential observational biases should also be considered. For example, a species may appear specialized simply because its host range has been insufficiently studied, whereas it may actually be a generalist.
8. Information regarding species validation (e.g., COI-based identification) would be more appropriately placed in the Materials and Methods section rather than in the Results.
9. A substantial portion of the Results section is devoted to describing species phylogeny, whereas the main focus of the study is the evolution of PCWDE genes. This section could be streamlined, with detailed phylogenetic descriptions moved to the Supplementary Materials.
10. In most cases, candidate HGT genes were identified primarily on the basis of phylogenetic evidence. Relying on a single line of evidence may be risky because some candidates could instead represent microbial contamination (e.g., GH28 sequences clustering with fungi, which commonly infect beetles), rapidly evolving endogenous genes (e.g., PL4 genes, depending on their sequence characteristics), or assembly artifacts. I strongly recommend incorporating at least one additional line of evidence for each putative HGT event, such as the presence of flanking eukaryotic genes in genomic regions, signal peptide analyses, genomic read support, or independent PCR validation. For example, flanking eukaryotic genes were examined for GH28 in the Araecerus genome, but similar analyses should ideally be performed for other GH28-containing lineages as well. Otherwise, the discussion and conclusions regarding HGT should be presented more cautiously.
11. Similar concerns apply to the patchy distribution of PCWDE genes among species. Such patterns may not necessarily reflect true biological absence and could instead result from insufficient sequencing depth, low transcript expression, incomplete transcript assembly, difficulties in assembling short genes, or cross-contamination among samples.
12. Regarding the PCA analysis, the separation among feeding-habit groups does not appear visually distinct, despite the significant results from permutational MANOVA. Therefore, I am not convinced that the PCA figure provides substantial additional biological insight. It may not be necessary to retain this analysis in the main text and could instead be moved to the Supplementary Materials.

Version 2:

Reviewer comments:

Reviewer #3

(Remarks to the Author)
The authors have addressed all of my comments thoroughly and point by point. I have no further comments and recommend that the manuscript be accepted in its current form.

Open Access This Peer Review File is licensed under a Creative Commons Attribution 4.0 International License, which permits use, sharing, adaptation, distribution and reproduction in any medium or format, as long as you give appropriate credit to the original author(s) and the source, provide a link to the Creative Commons license, and indicate if changes were made.
In cases where reviewers are anonymous, credit should be given to 'Anonymous Referee' and the source.
The images or other third party material in this Peer Review File are included in the article's Creative Commons license, unless indicated otherwise in a credit line to the material. If material is not included in the article's Creative Commons license and your intended use is not permitted by statutory regulation or exceeds the permitted use, you will need to obtain permission directly from the copyright holder.
To view a copy of this license, visit https://creativecommons.org/licenses/by/4.0/

Authors' responses are given below the reviewers' comments (in red font, preceded by ">>")

Reviewer #1:

This study utilized transcriptomes and genomes to explore the evolutionary dynamics of PCWDEs in weevils, most of which are horizontally transferred genes, and aimed to fill the gaps in our understanding of these genes in this large group of herbivorous beetles. The authors identified evolutionary patterns across 13 PCWDE families in weevils and found correlations between PCWDE repertoires and dietary specialization. This topic is compelling as it reveals the insight of correlation between special evolutionary events (e.g., HGT) and the ecological success of a taxa group.

Based on my experience, the final conclusion of the manuscript—the correlations between PCWDE repertoires and dietary specialization in beetles, particularly the reduction of PCWDEs in non-herbivorous beetles—appears reasonable and expected. However, I have three major concerns regarding this study, which may reduce its convincing power. To address these concerns, the authors should enhance their analyses to mitigate potential issues, or make the descriptions and discussions more rigorous and well-supported, providing necessary explanations and definitions within the manuscript.

>> We thank the reviewer for the overall positive evaluation and the constructive comments that have allowed us to improve the manuscript. We have addressed all the specific comments (see below).

1. The authors used transcriptomes to represent most species and even entire lineages. However, gene counts from transcriptomes often differ significantly from the true numbers in the genomes. Compared to genomes, transcriptomes face several challenges in providing accurate gene numbers and evolutionary patterns:
Redundant Gene Copies: Transcriptomes often contain multiple transcripts of the same gene, and misassembled transcripts, leading to gene counts that differ from the actual numbers and is positively correlated with the sequencing data size. Even after removing redundancy based on sequence similarity, these discrepancies remain, potentially compromising the correlation analysis between PCWDE repertoires and dietary specialization.

>> As detailed in the methods section, we used the *de novo* assembler implemented in CLC Genomics Workbench v21.0, which has (with the parameters we have set) a more relaxed threshold for collapsing e.g. allelic transcript variants and thus creates fewer, longer contigs compared to e.g., Trinity. Moreover, single individuals were used to reduce heterozygosity to a minimum. Additionally, the same assembly strategy was used for each of the datasets starting with similar sequencing data sizes. This is why we assume the same error rate (false positives or negatives) for each of the assemblies. Also, for each dataset we performed several assemblies by using 6 million reads, 25%, 50%, 75% and 100% of the total reads for each transcriptome to further minimize potential artefacts related to sampling size. Following their identification, each CAZyme was manually curated, which also reduces the risk of inflating gene numbers because of isotig/isoform misinterpretation. Still,

redundancy cannot be excluded but may also be a problem with genome data when allelic variants/heterozygous genomic regions are retained, e.g when purging of the original assembly is not efficiently collapsing alleles.

Missing Genes: Genes that are not expressed or have low expression under specific conditions may be absent from transcriptomes. While transcriptomes generally capture most genes present in genomes and offer a broad overview of gene presence, missing genes may be erroneously interpreted as gene losses, even though the genes may still exist in the genome.

>> We thank the reviewer for bringing up the potential expression level bias that may come with transcriptome data. However, insect PCWDEs are usually of a moderate to high expression level reducing the risk of underrepresentation in the transcriptomes. Moreover "missing genes" is not systematically biased but applies likely to each transcriptome in a similar way and thus would have minor impacts on the correlation analysis between PCWDE repertoires and dietary specialization.

Depending on the sequencing technology, genome structure and gene content variation, genome sequencing data may also miss genes erroneously for several reasons. First, depending on the sequencing technology, the genome size and other factors - such as genomic repeat length and complexity - genomic regions can be mis-assembled or are not represented in the final genome assembly at all. Second, highly similar genes (e.g., resulting from recent gene duplication events) might collapse when using Illumina short reads only. Third, multi-copy gene families can differ with respect to the copy number between individuals of the same species leading to over- or underestimations of gene numbers. Additionally, the level of completeness of a predicted gene set is only as good as the strategy used for genome annotation and prior repeat masking leading to missing genes simply because of them not being annotated.

As a benchmark we used four available datasets of *Araecerus fasciculatus* and compared pcwde gene numbers. We generated a transcriptome and a genome assembly in this study, the latter of which is included in an unpolished but also a polished version. A recently released genome assembly from another *Araecerus fasciculatus* population is included as well (ncbi: GCA_050578095.1). Results were obtained by BLAST searches using PCWDE sequences of the entire Curculionoidea as queries. Additionally, the two genomes were annotated using Helixer implemented in Galaxy (https://usegalaxy.eu/root?tool_id=helixer). Predicted gene sets were screened for CAZymes using dbcan3. Results are summarized in the Supplementary Table S1 showing that even two genome datasets from the same species but different locations and assembly strategies can differ not only with respect to gene number of a given gene family (8 to 12 GH5_2 genes) but also regarding the absence/presence of gene families (GH48). In contrast, our genome and transcriptome originating from the same population, showed no differences except for two GH5_2 pseudogenes that possess frame shifts on gene level and were absent from the transcriptome.

Outgroup Contamination: Transcript sequences are often short and lack flanking genes that would help verify whether contamination from outgroup species, particularly symbionts in the beetle gut, is present. Excluding the possibility of such contamination is critical when detecting HGT, but this is challenging to address using transcriptomes alone.

>> We are aware of the possibility of such kind of contamination and we cannot exclude them completely. However, as detailed in the methods section, poly-A+ mRNA was enriched prior library preparation, usually excluding the vast majority of prokaryote-derived mRNAs. Additionally, typical insect-like untranslated regions were identified for most of the transcripts. Thus, bacterial contamination can be excluded as prokaryotes usually lack both poly-A and UTRs. Nevertheless, we agree with the reviewer that contaminants, such as fungal or protozoan transcripts can be problematic and also AT-biased bacterial symbiont genomes could result in transcript capture during poly-A+ enrichment. To address the reviewers´ concern and to identify potential contaminants, we conducted taxonomic classifications of our transcriptome assemblies by kraken2 (version 2.1.3) using exact k-mer matches. Kraken2 implemented in Omicsbox (version 3.5.3) was used with confidence filter set 0.4, minimum hit groups set 2 and the reference database RefSeq 2024-11 WGS to detect "outgroup contamination". Contigs classified as either bacteria or eukaryotes were extracted and used as a database to run tBLASTn and BLASTn with the previously annotated PCWDEs as queries. No hits indicating contamination were found in any of the assembled transcriptomes. A paragraph was added to the material and methods section of the revised version of the manuscript describing the procedure (line 251 ff.).

Cross-contamination: Transcriptomes are also susceptible to cross-contamination, where sequences from other species in the same sequencing batch may be incorporated into the transcriptome, distorting the reconstruction of HGT gene evolutionary history.

>> Spill-over can only occur when libraries are sequenced on the same lane, which rarely happened for the species sequenced in our case. For the samples that were processed on the same lane, the following would be expected to happen in case of contamination issues: two identical transcripts in two datasets (two species) are present with the donor-library to be expected to have a much higher expression level compared to the recipient library. We are aware of the risk of cross-contamination and manually checked for identical sequences but never found indications of cross-contamination. Moreover, each dataset was COI barcoded (see table S2) and only a single COI sequence was found per transcriptome. As COI is usually highly expressed, a cross-contamination should have become obvious.

Overall, I disagree with treating transcriptome data with the same weight as genome data in the analyses. I believe genome data should be considered primary, with transcriptome data serving as supplementary.

>> We politely disagree with the reviewer in considering genome data primary and transcriptome data accessory for the reasons detailed above. In general, each sequencing technology comes with its own pros and cons. Whereas transcriptomes are always snapshots and genomes the blueprint of all living beings, the challenges that go along with genome assembly and proper gene annotation (which are often ignored) can lead to similar artifacts the reviewer summarized for transcriptomes, including missing genes, chimeras, redundant genes and contaminations, which altogether can have an impact on real gene numbers. Furthermore, benchmarking our approach with two different *Araecerus fasciculatus* genomes and a transcriptome revealed the same PCWDE results for the genome and transcriptome of the same beetle population, indicating that the transcriptome data reliably reflect

PCWDE numbers.

2. The authors sequenced and assembled a new genome of Araecerus fasciculatus. However, this genome appears to have been assembled solely from Nanopore reads, without subsequent polishing using Illumina reads. The accuracy of a genome assembled from Nanopore reads may not be sufficient, which could explain why the genes from this species appear unusual and occasionally cluster with genes (e.g., GH28, GH5_2) from species in other lineages.

>> We thank the reviewer for pointing out the need for polishing Nanopore reads. Previously obtained PCWDEs from the *Araecerus* transcriptome and unpolished genome were used as queries in a BLASTn against the polished genome assembly as a database. All queries were covered to 100% and sequence identity was between 99-100% on nucleotide level. No additional PCWDE family or additional genes of the previously identified PCWDE families were found by using a set of other weevil-encoded PCWDEs as queries. Nevertheless, both polished and unpolished genomes will be uploaded to Edmond, the Open Research Data Repository of the Max Planck Society.

In addition, PCWDE nucleotide sequences were used as queries in a BLASTn against the *Araecerus fasciculatus* genome recently released (ncbi: GCA_050578095.1) and generated from the highly accurate PacBio sequencing technology. Sequence identity was between 96-98% on nucleotide level and queries were covered to 100%.

Moreover, we want to highlight here that we also generated a transcriptome for *Araecerus fasciculatus* (larva). Sequence comparison between the manually curated GH5_2 and GH28 ORFs revealed 96-100% sequence identity on nucleotide level and 100% on amino acid level. Gene trees were built on amino acid sequences. Thus, we can be pretty sure that sequencing errors did not affect GH28 and GH5_2 phylogenetic placement.

3. The authors frequently propose intra-phylum HGT in the manuscript, which I find somewhat arbitrary and insufficiently supported. It is not uncommon for HGT genes from distant lineages to cluster together in a clade. Similar sequence compositions and conserved intron sites may also be the result of convergent evolution of these HGT genes under selective pressures in insect genomes. And the systematic errors in gene phylogenetic reconstruction should be considered. Unless more robust supporting evidence is presented, I suggest that the concept of intra-phylum HGT should be treated as a possibility and discussed more conservatively, even if the authors lean toward this hypothesis.

>> We took the reviewers suggestions into account and toned down the intra-phylum passages and we now discuss them as one potential scenario among others.

Other comments:

Line 54-59: The authors describe PCWDEs derived from bacterial symbionts or the insects' own genomes. I agree with the categorization of PCWDEs into these two sources (although PCWDEs may also be secreted by symbiotic fungi or protists, in addition to bacteria). However, from the perspective of a specific insect, these two types of PCWDEs are not always distinct. For instance, most insects possess GH1 and GH9 genes (which are not derived from HGT) in their genomes, which can also aid in the digestion of plant cell wall components. Furthermore, most herbivorous insects harbor symbionts that

produce PCWDEs. Therefore, I suggest refining these sentences to make them more precise, focusing on the two sources (symbionts and insects themselves) rather than on insect lineages.

>> We thank the reviewer for pointing out this imprecise wording. This passage has been rephrased accordingly.

Line 85-87: I would like to know how many dietary types have been included in previous studies. Since this article primarily addresses the feeding ecology of weevils, improving the representation of diets is important. I note that McKenna et al. (2019) examined about half of the weevil subfamilies, suggesting that further improvements in subfamily representation in this study may not be particularly significant. Therefore, if the enhancement of diet representation is substantial, I recommend that the authors mention it here.

>> We agree with the reviewer that covering additional weevil subfamilies compared to taxon sampling in previous studies does not automatically increase the representation of different diets. Additionally, we are aware of the fact that fungivory and mixed-feeding like xylomycetophagy is not well represented in our dataset, similar to previous ones. Compared with McKenna et al. 2019 we added reproductive organ feeding Belidae, Anthribidae, Scolytinae as well as a vascular tissue feeding Lixinae species. Thus, within certain already previously covered clades, our dataset provides more dietary shifts by adding more species. With respect to previously uncovered subfamilies, no overall additional dietary type was added based on the level of classification we applied. However, by including additional species, each of the dietary types is represented by a higher number of species compared to previous studies, increasing the statistical power to detect differences in PCWDE gen sets correlating with diet.

Line 123: For analysis?

>> "RNA-Seq analysis" was replaced by "RNA sequencing".

Line 149: Please provide the rationale or reference for selecting the midgut.

>> We believe that the reviewer´s comment is based on a misunderstanding. We have not selected RNA-Seq data from midguts only but excluded data from samples that lacked midgut tissue for RNA extraction. The rationale of this criterion is that beetle-encoded PCWDE genes are expressed in the gut, since PCWDEs are digestive enzymes and are secreted into the gut lumen to fulfill their function.

Line 233-234: I am somewhat confused by this analysis. Does it use cDNA sequences from this species to align with the genome sequences? If so, it would not detect newly discovered genes or genes absent from the transcriptomes. Or does it use cDNA sequences from other species to align with the genomes? The method description should be clarified for better understanding.

>> This passage applies only to *Apoderus coryli* and *Araecerus fasciculatus*. In case of the *Apoderus coryli* genome, cross-species BLAST was used with queries obtained from the *Apoderus praecellens* transcriptome. In case of *Araecerus fasciculatus*, PCWDE queries from the transcriptome were used to run BLAST against the genome assembly. In both cases genes absent from transcriptomes or present in a truncated version could potentially be discovered by running tBLASTn, as a relaxed cut-off (Expect value of 0.00001) was used. Details were added accordingly to the paragraph.

Line 237: The method or criteria used to determine HGT donors should be provided.

>> Beetle PCWDEs curated from our transcriptomes were used as queries in BLASTp searches with relaxed constraints against the NCBI nr database. Top-100 hits were taken into account for each gene family. Following these sequence extractions, phylogenetic analyses were performed to elucidate the closest relatives of the beetle PCWDEs in our datasets. We added a more detailed description in the Methods section to explain how sequences were retrieved.

Line 240: Please provide the rationale for removing redundancy at the 90% identity level.

>> It is important to note that this identity cutoff applies only to the non-beetle-sequences extracted from NCBI and not to the sequences obtained from the transcriptomes and genomes from the beetles. The rationale of using this cutoff is to reduce computing time by using a single representative sequence per cluster of sequences sharing 90% identity or more.

Line 249: A two-factor MANOVA could be used to assess the impact of both adult and larval diets, if feasible.

>> We appreciate the reviewer's suggestion to consider a two-factor MANOVA incorporating both adult and larval diets. In this study, we evaluated the effects of larval and adult dietary preferences on gene copy number variation using separate phylogenetically informed MANOVAs. This approach allowed us to assess the contribution of each life stage independently while accounting for shared evolutionary history.

Line 251: Since PCWDEs is not mentioned for the first time, the full term should not be provided here.

>> Done.

Line 276-277: According to the data mentioned in Line 94 (only 2-4 subfamilies are absent in this study), do these two absent families consist of only one subfamily (or even a single genus/species) in each case? I suggest mentioning this or describing the absence at the subfamily level.

>> We did not include Rhinorhynchinae, Nemonycinae, Conoderinae sensu strictu and Mesoptiliinae. We did not take into account the four smaller groups of unresolved phylogenetic position and relationships in Brentidae for subfamily counting, the Eurhynchinae, Ithycerinae, Microcerinae and Nanophyinae. These details were added to the methods section accordingly (line 115 ff.).

Line 520: GH9 is missing. While I understand that GH9 is not the focus of this study's HGT analysis, it is shown in the results and should at least be briefly discussed.

>> We share the opinion of the reviewer about the GH9 to some extent and also briefly discussed this gene family in an earlier version of the manuscript. However, we decided to remove it from the final version as it is not HGT derived. Interestingly, its widespread presence in non-herbivorous insects including non-herbivorous beetles strongly indicates a role independent from herbivory. As, on the other hand, there are some functionally characterized insect GH9 cellulases, we decided to also list

this gene family in our study (Figure 1 and supplementary tables). Now that the reviewer is pointing out the inconsistency of showing genes presence in Figure 1 but not discussing it, we mention GH9 in the context of widely distributed PCWDEs.

Line 526-537: Regarding the patchy distribution of HGT hemicellulases in independent insect lineages, one possibility is that the mechanisms for hemicellulose digestion are already present in most insects (e.g., via GH1 or symbionts).

>> We raised the possibility of symbionts being the source of hemicellulases in a dedicated paragraph entitled "The hemicellulases perspective: Functional implications" in our discussion (line 623 ff.). In this part of the manuscript, we also pointed out three additional potential reasons for why hemicellulases are so patchily distributed.

We thank the reviewer for bringing up the idea of GH1 proteins being involved in hemicellulose digestion. GH family 1 is one of the biggest families of CAZymes and a moderately sized gene family in insects with highly diverse functions. Whereas several studies showed that GH1 proteins contribute to cellulose hydrolysis in bacteria and a few insects (cellobiosidase activity), there is very limited information about other plant cell wall degrading enzymatic activities in this gene family. In termites it has been shown that a bacterial GH1 possesses xylosidase activity and some free-living bacteria encode GH1 exo-enzymes that might hydrolyze short galactose and arabinose side-chains of plant cell wall polysaccharides. In summary, we decided against discussing GH1 proteins as a potential source of hemicellulose activity due to the lack of sufficient functional data. Still, we believe that a comprehensive functional screening of GH1 enzymes in herbivorous insects might reveal new activities so far not assigned to this gene family.

Reviewer #2:

Comments to Authors.

This manuscript presents a comprehensive analysis of the distribution and evolution of plant cell wall-degrading enzymes (PCWDEs) across 24 representative subfamilies of the superfamily Curculionoidea, based on both transcriptomic and genomic data. The study provides new insights into the relationships between lineage-specific gene family expansions or contractions and feeding ecology of this group. The dataset is extensive, and the work represents a valuable contribution to understanding the molecular evolution underlying dietary adaptation in beetles. **However, several aspects of the phylogenetic interpretation, the definition and categorization of feeding types, and the treatment of phylogenetic non-independence remain somewhat unclear**. Further clarification and elaboration on these points would strengthen the reliability of the conclusions.

>> We appreciate the reviewer's time to read the manuscript and provide feedback.

Major comments:

1. Classification of feeding categories

The authors categorize feeding habits into four groups based on the plant tissues utilized: reproductive organs (R), photosynthetic organs (P), vascular tissues (V), and non-plant-based fungal feeding (F). However, larvae of many weevils and longhorn beetle species feed on decaying wood (mixture of wood and fungal mycelia). The treatment of such "xylomycetophagous" feeders is not clearly described. It

should be clarified which category these species were assigned to, and the potential impact of omitting the intermediate category on the results—especially the correlation analyses between feeding type and gene family size—should be discussed.

>> We thank the reviewer for highlighting the diversity of food sources in weevils and we are aware of the limitations of our broader category assignments. To the best of our knowledge, the only species in our dataset that might be xylomycetophagous is the larval stage of *Platyrhinus resinosus*. To accommodate for the lack of detailed information about the feeding habits in this species we coded its feeding habit F/F (fungivore in larval and adult stage).

2. Phylogenetic inference
Although this study includes a large number of loci and a wide taxonomic range, the differences between the resulting phylogeny and previous works (e.g., Shin et al. 2018) are not sufficiently discussed. For example, the relationships among families outside Curculionidae, such as Belidae, are not mentioned. Furthermore, for branches with low support values, it should be clarified to what extent these reflect technical issues. The potential implications of the obtained topology for the higher-level classification of Curculionoidea could also be briefly discussed.

>> We thank the reviewer for pointing out the different phylogenetic placement of Belidae (sister of Cimberididae) compared to Shin et al. 2018 (sister of Anthribidae + Rhinorhynchinae). Unfortunately, we did not have access to Rhinorhynchinae or Caridae. In contrast, these two clades were present in Shin et al. 2018. Such sampling differences may already cause the different affiliations of Belidae in the phylogenetic analysis. However, as we mainly focused on weevil PCWDEs and not their species phylogeny, we decided to not include a discussion about similarities and differences compared to previous topologies although the latter is a fascinating topic on its own. Moreover, we believe that the different placements of Belidae in the two studies have no impact on the understanding of PCWDE evolution in weevils. For example, both topologies support the hypothesis that Anthribidae lost previously acquired PCWDE families (GH45, 48, 28), as both Belidae and Cimberididae possess them and McKenna et al. 2019 revealed their presence also in Rhinorhynchinae.

Moreover, we want to mention here a recent study focusing on the Cimberididae, which revealed the same sister-group relationship with Belidae as we did (doi: 10.14411/eje.2024.047). Again, although these findings are worth to discuss in general, we believe they would at the same time distract the reader from our main focus: the evolutionary history of weevil PCWDEs.

3. Feeding habits within lineages
It is unclear to what extent the sampled taxa represent the ecological diversity of their respective subfamilies or lower groups. This could affect the inferred relationships between feeding type and PCWDE gene family size. This is particularly important when considering lineage-specific patterns of gene family dynamics in relation to evolutionary time (divergence age). For example, Hypothenemus hampei is classified as feeding on reproductive tissues (R), but many congeners are phloeophagous (feeding on inner bark) or myelophagous (feeding in twig pith), suggesting a relatively recent dietary shift. In contrast, groups such as Platyopodinae appear to have maintained fungal feeding since the

common ancestor of the subfamily. Such evolutionary and sampling contexts should be clarified to aid interpretation.

>> We completely agree with the reviewer that ideally sampled taxa should reflect the ecological diversity of the respective clade. However, although we tried to cover as many feeding habits as possible, this is a challenging task in such a diverse group like weevils, especially within the Scolytinae. This is also why we did not attempt to correlate food source with PCWDE composition in lower taxonomic units (e.g., within subfamilies) but instead focused on the broad pattern. Adding a temporal dimension to the PCWDE evolutionary dynamics by comparing recent and old dietary shifts, would definitely be an interesting future direction!

4. Statistical support for the reduction of gene family size in fungivorous taxa
Although the reduction in PCWDE gene numbers among fungivorous lineages appears reasonable result, the small sample size and the issue of phylogenetic non-independence call for a cautious interpretation. The authors applied a phylogenetically corrected multivariate analysis using procD.pgls(), but the statistically significant relationship seems to be limited to the overall correlation between feeding type and total gene number. This should be explicitly stated, and the reduction in fungivorous taxa should not be overemphasized if it lacks robust statistical support.

>> We thank the reviewer for this thoughtful comment regarding sample size and phylogenetic non-independence. The small number of fungivorous species in our study is indeed problematic. However, even by an increase of sample size, the issue of phylogenetic non-independence will not be solved, as shifts to a fungal diet evolved just in a limited number of weevil taxa. As noted, our phylogenetically corrected MANOVA, procD.pgls() identified a significant association between overall dietary category and gene copy number variation, but we refrain from drawing strong conclusions about specific dietary groups unless supported by sufficient sample size and phylogenetic structure. Nevertheless, we now explicitly state this limitation in the discussion and avoid over-interpreting reductions observed in individual fungivorous lineages.

Minor comments:
L67: Kirsch et al., 2025 did not investigate longhorn beetles (Cerambycidae). Is this a miscitation or omission?

>> The reference has been removed.

L324: There seem to be three rather than four events; two Brentinae species lost the gene but form a single clade, so these losses are not independent.

>> We agree with the reviewer and have changed the manuscript accordingly.

L462: Euparius marmoreus is not shown in the phylogenetic tree.

>> Transcriptome assemblies from the publicly available *Euparius marmoreus* Illumina reads (SRR2083663) revealed ~30% of insect BUSCO genes as "missing". Thus, this dataset was screened for PCWDEs and results are represented in the supplementary table. However, we refrained from

including *Euparius* in the species phylogeny, as this would have reduced the number of BUSCO genes common to all datasets, i.e. the genes our species phylogeny was inferred from.

L470: The text states “five species feed on fungi,” but only four are shown in the phylogeny.

>> We added an explanation for this discrepancy. Euparius was not added in the phylogenetic reconstruction due to its low BUSCO stats (line 494).

L550: Same issue as L67.

>> A reference covering Cerambycidae was added here.

Authors' responses are given below the reviewers' comments (in red font)

Reviewer #1:

The authors have made substantial improvements in response to the previous round of reviews. They have addressed most of my concerns, thereby strengthening the manuscript. I particularly appreciate the benchmarks and checks conducted by the authors to demonstrate the relative reliability of their transcriptomic data. However, the authors also acknowledge that the potential risk of misestimated gene numbers (e.g., redundancy) in the transcriptomes persists. For stringency, I suggest that the authors mention this in the discussion section.

We appreciate the reviewer's time to read the manuscript again and provide feedback.

We want to highlight that we added a *Limitation of the Study* statement at the end of the Discussion section (lines 776-788), to especially address the potential risk of misestimated gene numbers in our analyses by saying: "A limitation of this study is that it relies predominantly on transcriptome data rather than complete genome assemblies. Consequently, estimates of gene copy number may not fully capture the entire genomic repertoire, as both underestimation (owing to genes not expressed under the sampled conditions) and overestimation (resulting from assembly artifacts or transcript redundancy) remain possible. To minimize such biases, all datasets were processed using an identical assembly and annotation pipeline, and each PCWDE candidate was subjected to manual curation, thereby reducing methodological inconsistencies among species. We therefore expect any residual false-positive or false-negative rates to be broadly comparable across assemblies. Furthermore, PCWDEs are typically expressed at moderate to high levels, which likely reduces the risk of their underrepresentation in transcriptome-based datasets. Nevertheless, the availability of additional high-quality, chromosome-level Phytophaga genomes will enable further refinement and validation of the gene repertoires reported here."

There are a few other issues in the revised manuscript:

Lines 139-142: The step involving the assembly of different read proportions appears abruptly in the manuscript. From the reader's perspective, there is either no corresponding result of the impact assessment, or no description of the downstream purpose of this step, which may be to manually curate the CAZymes, as mentioned in the response. I recommend adding downstream content of this step within the manuscript.

As mentioned in our previous point-by-point response, for each dataset we performed several assemblies by using 6 million reads, 25%, 50%, 75% and 100% of the total reads for each transcriptome to further minimize potential artifacts related to sampling size. We agree with the reviewer that a more specific description of the impact of this strategy would be helpful to the reader methodologically interested in *de-novo* assembling. Generally, it is known that Illumina read coverage can affect transcriptome assembly results. While low coverage leads to fragmented, incomplete transcripts, excessive coverage creates its own issues, increasing false-positive splice variants, computational bloat, and artifactual chimeric fusion transcripts. As genes are differentially expressed,

a specific read proportion used for *de-novo* transcriptome assembly might be optimal for many but not all genes. We were aware of such phenomena and thus generated several assemblies per sample with different read depths. Actually, we found PCWDE-encoding transcripts full-length or fragmented depending on the read proportions. However, as these phenomena are widely discussed in the literature and we are not aiming at a methodological focus of the paper, we decided not to go into further detail. To take the comment of the reviewer into account, we added "It is known that Illumina read coverage directly shapes transcriptome assembly. While low coverage leads to fragmented, incomplete transcripts, excessive coverage creates its own issues, increasing false-positive splice variants and artifactual chimeric fusion transcripts. As genes are differentially expressed, one specific read proportion used for de-novo transcriptome assembly might be optimal for many but not all genes." to the corresponding methods section (lines 144-149), to explain what is meant with "artifacts related to sampling size".

Line 244: "pcwde" should be capitalized.

Done.

Reviewer #3:

This study addresses a highly interesting and important research topic, as it may help explain why phytophagous beetles, particularly plant-feeding lineages such as weevils, have achieved remarkable species diversity. I first examined the revised manuscript and then carefully reviewed the previous reviewers' comments together with the authors' responses. While the authors have made substantial efforts to address earlier concerns, I would like to provide several additional comments.
My primary concern relates to the reliability of the identified genes, which overlaps considerably with issues raised by previous reviewers. Most of the PCWDE genes analyzed in this study were derived from transcriptome assemblies. Consequently, assembly errors, insufficient sequence depth, microbial contamination, and cross-sample contamination during sequencing remain potential sources of bias. If these issues are not adequately addressed, some of the inferred evolutionary patterns of PCWDEs, including horizontal gene transfer (HGT) events, may be misinterpreted. My specific comments are as follows:

We thank the reviewer for the overall positive evaluation and the constructive comments that have allowed us to improve the manuscript. We have addressed all the specific comments (see below).

1.As far as I know, sequencing based on the MinION platform is associated with relatively high error rates. Although several polishing and error-correction approaches are available, the authors should explain why this platform was chosen instead of alternatives such as PromethION or PacBio, and provide evidence demonstrating that the resulting genome assembly is sufficiently accurate and complete for reliable target-gene identification and evolutionary analyses.

We thank the reviewer for pointing out again that MinION compared with PacBio, especially in the early phase of release (including chemistry for library preparation), was more prone to sequencing errors. We generated the *Araecerus* genome dataset already three years ago and we did not have access to PromethION back then. In general, whether using the MInION or PromethION sequencer does not have an impact on error levels, but instead the flow cell type, library kit and software for basecalling is relevant. Reviewer #1 previously pointed out these potential problems related to ONT-only data. This is why we generated Illumina-based sequencing data for polishing the ONT-assembly as a response to the reviewer comment before resubmission of our manuscript and added relevant details to the Material and Methods section as follows (lines 203-205) "For final error correction, ntEdit was used with ~25 Gb paired-end (2 × 150-bp) Illumina data generated from *A. fasciculatus* HMW DNA." No additional PCWDE family or additional genes of the previously identified PCWDE families were found by using a set of weevil-encoded PCWDEs as queries. Nevertheless, both polished and unpolished genomes will be uploaded to Edmond, the Open Research Data Repository of the Max Planck Society.

2.Please clearly describe the contents of Supporting Data 2.

As indicated in the Data Accessibility Statement, supporting data 2 contains the codon-based nucleotide alignment (FASTA file), the IQ-TREE log file and the resulting tree file (Newick file) used to generate Fig. 1, the weevil species phylogeny. The caption of Fig. 1 provides details about how the tree was generated by saying "A maximum likelihood analysis, based on a codon-based nucleotide sequence alignment of 107 BUSCO single copy orthologous genes (containing 78,321 sites) common to all genome and transcriptome datasets was conducted using the program IQ-TREE with 1,000 ultrafast-bootstrap replicates." We would like to address the point raised by the reviewer, but are not sure what kind of additional information would be required to further describe supporting data 2.

3.The authors attempted to evaluate potential contamination from bacteria, fungi, and protozoa, as discussed in their response to Reviewer 1. However, potential cross-contamination among RNA-seq samples is also an important concern because it may directly affect the detection and interpretation of candidate HGT genes. The authors stated that they manually checked for identical sequences and examined COI sequences, but these approaches alone do not adequately quantify or exclude potential cross-contamination across the entire dataset. Additional analyses using software such as CROCO, together with independent validation methods (e.g., PCR amplification in additional individuals of the same species), would substantially strengthen confidence in the results.

We are aware of the risk of cross-contamination and thank the reviewer for pointing this out again, as it can cause problems in downstream analyses. However, spill-over can only occur when libraries are sequenced on the same lane, which rarely happened for the species sequenced in our case. For the samples that were processed on the same lane, the following would be expected to happen in case of contamination issues: two identical transcripts in two datasets (two species) are present with the donor-library to be expected to have a much higher expression level compared to the recipient library. This expectation/assumption is exactly what CroCo takes into account: i) high transcript level in general, ii) contamination of lower abundance in the contaminated sample compared with the "donor" sample, and iii) high level of sequence identity (> 95% on nucleotide level) over a fragment > 40 nucleotides. Still, we agree with the reviewer that CroCo would be an unbiased way of detecting

cross-species contamination. However, as we are focusing on PCWDE families and not aimed at cross entire-dataset comparisons, we believe that cross-species BLAST using these PCWDEs as queries are sufficient to test for potential carry over during library processing or sequencing. We did not find a single PCWDE that was present in more than one species (datasets) showing more than 90 % sequence identity at the amino acid level.

4.Were any methods applied to treat or filter alignment gaps in the PCWDE protein sequence alignments? Please clarify.

No additional gap-trimming or gap-filtering steps were applied to the PCWDE amino acid sequence alignments. After alignment, the sequences were manually inspected only for obvious alignment errors. The final alignments, with gaps retained, were directly used for IQ-TREE phylogenetic analyses. The final alignment files are provided as supporting Data 3.

5.Please note that removing redundant sequences at a 90% identity threshold may influence the inference of HGT donor lineages. A preferable approach would be to retain all closely related sequences in phylogenetic analyses while reducing only highly redundant representatives from the same taxonomic groups and with nearly identical sequences.

We thank the reviewer for this important observation. We agree that the choice of sequence redundancy threshold can potentially influence the representation of closely related taxa and, consequently, the inferred phylogenetic relationships used to identify putative HGT donor lineages. However, we kindly want to point out that the 90 % identity threshold only applies to the backbone phylogenies (sequences taken from ncbi) and not to sequences identified in our datasets. Thus, there was no chance to eliminate publicly available sequences that were very similar to the ones we sequenced ourselves.

In this study, we applied a 90% sequence identity threshold to reduce dataset size and overrepresentation of highly similar sequences. Our objective was to obtain a manageable and taxonomically diverse set of homologs for phylogenetic reconstruction. While retaining all near-identical sequences could provide additional resolution within specific clades, we do not expect the removal of highly similar sequences to substantially affect the broader phylogenetic placement of the focal genes or the major evolutionary patterns that support our HGT inferences, as known HGT event are ancient.

To clarify this point, we have added text to the Methods section (lines 275-285) acknowledging that sequence clustering at 90% identity may reduce representation of some closely related lineages and therefore could influence fine-scale donor attribution. Again, the 90 % identity threshold only applies to the backbone phylogenies (sequences taken from ncbi) and not to sequences identified in our datasets. Thus, there was no chance to eliminate publicly available sequences that were very similar to the ones we sequenced ourselves. "We note that clustering sequences at 90% identity may reduce representation of closely related taxa and could affect fine-scale inference of HGT donor lineages. However, this approach was adopted to minimize redundancy and computational burden while preserving the broader phylogenetic signal relevant to our study objectives. Moreover, based on previous findings PCWDE gene transfers did not happen recently, which makes it extremely unlikely that a donor sequence shares > 90% identity with the beetle counterparts and is not included in the

phylogenetic analyses because of that. Furthermore, the 90 % identity threshold only applies to the backbone phylogenies (sequences taken from ncbi) and not to sequences identified in our datasets. Thus, there was no chance to eliminate publicly available sequences that were very similar to the ones we sequenced ourselves." We believe this approach remains appropriate for the goals of the present study and does not alter the principal conclusion.

6.What criteria were used to classify genes into the three categories: cellulolytic enzymes, hemicellulolytic enzymes, and pectolytic enzymes? Please provide the classification standard.

We added more information to the Methods section (lines 245-247) to clarify the classification of CAZymes by saying "More precisely, CAZymes were classified into cellulases, pectinases and hemicellulases according to the functional data linked with each gene family on the CAZY webpage (https://www.cazy.org/)." The CAZy database describes the families of structurally-related catalytic and carbohydrate-binding modules (or functional domains) of enzymes that degrade, modify, or create glycosidic bonds and thus functions as the classification standard.

7.It would be helpful to provide the feeding habits of each species together with supporting references. Because these data form an important basis for downstream analyses, potential observational biases should also be considered. For example, a species may appear specialized simply because its host range has been insufficiently studied, whereas it may actually be a generalist.

Potential observational bias affecting the categorization of an herbivore along the generalist-specialist gradient is an important point raised by the reviewer. However, correlating host plant range with gene numbers of PCWDE families is beyond the scope of our study and we are aware of the challenges to group herbivorous insects into poly-, oligo- or monophagous, especially when also taking larval stages into account, which are usually understudied. This is why we restricted our categorization to very broad feeding habits: F, fungi; P leaves; V, vascular tissue; R, reproductive organs. We agree with the reviewer that correlation between metabolic capacities (set of PCWDEs) and diet breadth (set of host plant taxa) would be very interesting but we are afraid that our current taxon sampling and the amount of information available on their diets is not sufficient to address this aspect.

8.Information regarding species validation (e.g., COI-based identification) would be more appropriately placed in the Materials and Methods section rather than in the Results.

We are principally in line with the reviewer in describing species identification in the Methods not in the Results section. However, in this specific case we politely disagree with the reviewer and believe that the incorrect taxonomic assignment of a publicly available transcriptome dataset is it worth mentioning in the Results section and we therefore prefer to keep its placement as is.

9.A substantial portion of the Results section is devoted to describing species phylogeny, whereas the main focus of the study is the evolution of PCWDE genes. This section could be streamlined, with detailed phylogenetic descriptions moved to the Supplementary Materials.

We appreciate the reviewer´s suggestion to further streamline the Results section by moving phylogenetic inference to the Supplement. We totally agree with the reviewer about the focus of our study: the evolutionary dynamics of PCWDEs. At the same time, we believe that the weevil species

phylogeny is the basis for the conceptualization of PCWDE evolutionary history. Moreover, we want to call the reviewer´s attention to a comment raised by the previous #Reviewer2 about the species phylogeny and its discussion in our study. This reviewer was asking for more instead of less description and discussion about the tree topology and its implications. We believe that the current version of this part of the Results section accommodate these very different views on how pronounced the species phylogeny should be explained in our study.

10.In most cases, candidate HGT genes were identified primarily on the basis of phylogenetic evidence. Relying on a single line of evidence may be risky because some candidates could instead represent microbial contamination (e.g., GH28 sequences clustering with fungi, which commonly infect beetles), rapidly evolving endogenous genes (e.g., PL4 genes, depending on their sequence characteristics), or assembly artifacts. I strongly recommend incorporating at least one additional line of evidence for each putative HGT event, such as the presence of flanking eukaryotic genes in genomic regions, signal peptide analyses, genomic read support, or independent PCR validation. For example, flanking eukaryotic genes were examined for GH28 in the Araecerus genome, but similar analyses should ideally be performed for other GH28-containing lineages as well. Otherwise, the discussion and conclusions regarding HGT should be presented more cautiously.

We completely agree with the reviewer that ideally for each proposed HGT-event (each independent acquisition of a PCWDE-encoding gene), genomic information should be provided. However, for most of the PCWDE families, potential donors were already discussed previously and the presence of flanking insect genes shown. Relevant literature is cited in the introduction (Wybouw, et al. 2016, McKenna, et al 2019). This is why we restricted ourselves to show genomic context only for gene families i) reported for the first time in animals (GH43_6), ii) the first time in weevils (*Araecerus* GH5_2) or iii) derived from a not yet described donor (*Araecerus* GH28). In case of the weevil PL4_4 gene family, we trust that the presence of introns rules out microbial contamination.

11.Similar concerns apply to the patchy distribution of PCWDE genes among species. Such patterns may not necessarily reflect true biological absence and could instead result from insufficient sequencing depth, low transcript expression, incomplete transcript assembly, difficulties in assembling short genes, or cross-contamination among samples.

Like above, we agree with the reviewer that ideally for each proposed HGT-event (each independent acquisition of a PCWDE-encoding gene), genomic information should be provided. Moreover, the potential issue of missing PCWDEs in transcriptomic vs genomic datasets is of course a valid point, but we believe that we already acknowledge this above and in our *Limitation of the study* statement.

12.Regarding the PCA analysis, the separation among feeding-habit groups does not appear visually distinct, despite the significant results from permutational MANOVA. Therefore, I am not convinced that the PCA figure provides substantial additional biological insight. It may not be necessary to retain this analysis in the main text and could instead be moved to the Supplementary Materials.

We consider the visualization of the PCA analysis (i.e., the PCA figure) to be important to include in the main manuscript especially because at a first glance the groups do not appear to be clearly distinct – but the permutational MANOVA tests were significant. What figure panels a-f show is that for each

PCWDE family there is a distinct clustering pattern in the PCA analysis, which is informative in its own right. We would therefore prefer to retain this analysis and its visualization in the main text.

Authors' responses are given below the reviewers' comments (in red font)

Reviewer #3 (Remarks to the Author):

The authors have addressed all of my comments thoroughly and point by point. I have no further comments and recommend that the manuscript be accepted in its current form.

We highly appreciate the reviewer's time to read the manuscript again and we are happy that our work is finally recommended for publication.
