# Supplementary material for: Evolutionary dynamics of plant cell wall-degrading enzymes reflects feeding ecology in weevils
Source: Commun Biol. 2026 Sep 23;9:1243. doi: 10.1038/s42003-026-10927-0 (PMC13601563; doi:10.1038/s42003-026-10927-0)
Supplement: Supplementary file 2 [file 42003_2026_10927_MOESM2_ESM.pdf]

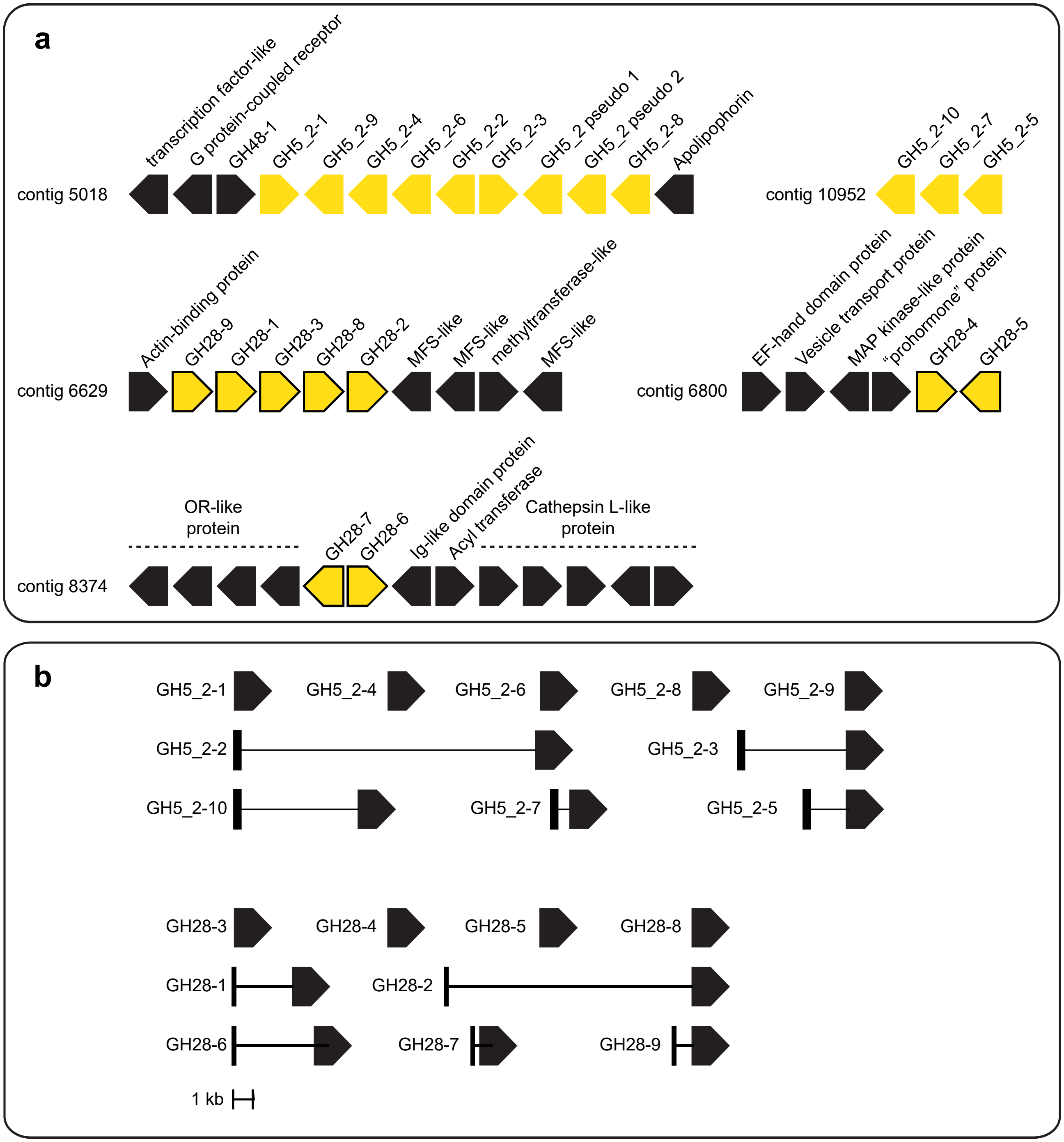

Figure S1. Gene Organization and Structure of GH5_2 and GH28 in the *Araecerus* Genome. (a) The arrangement of GH5_2 (yellow) and GH28 (yellow enframed) genes on draft genome contigs is illustrated, including the identified flanking genes. The representation is not drawn to scale. (b) The structure of both GH5_2 and GH28 genes is presented. Exons are depicted as blocks, and introns are represented by thin lines. Each gene structure is drawn to scale, with a scale bar indicating 1 kb. It is noteworthy that the intron detected in several GH28 genes does not disrupt the open reading frame but is located in the 5'-untranslated region (see Figure S2 for details).

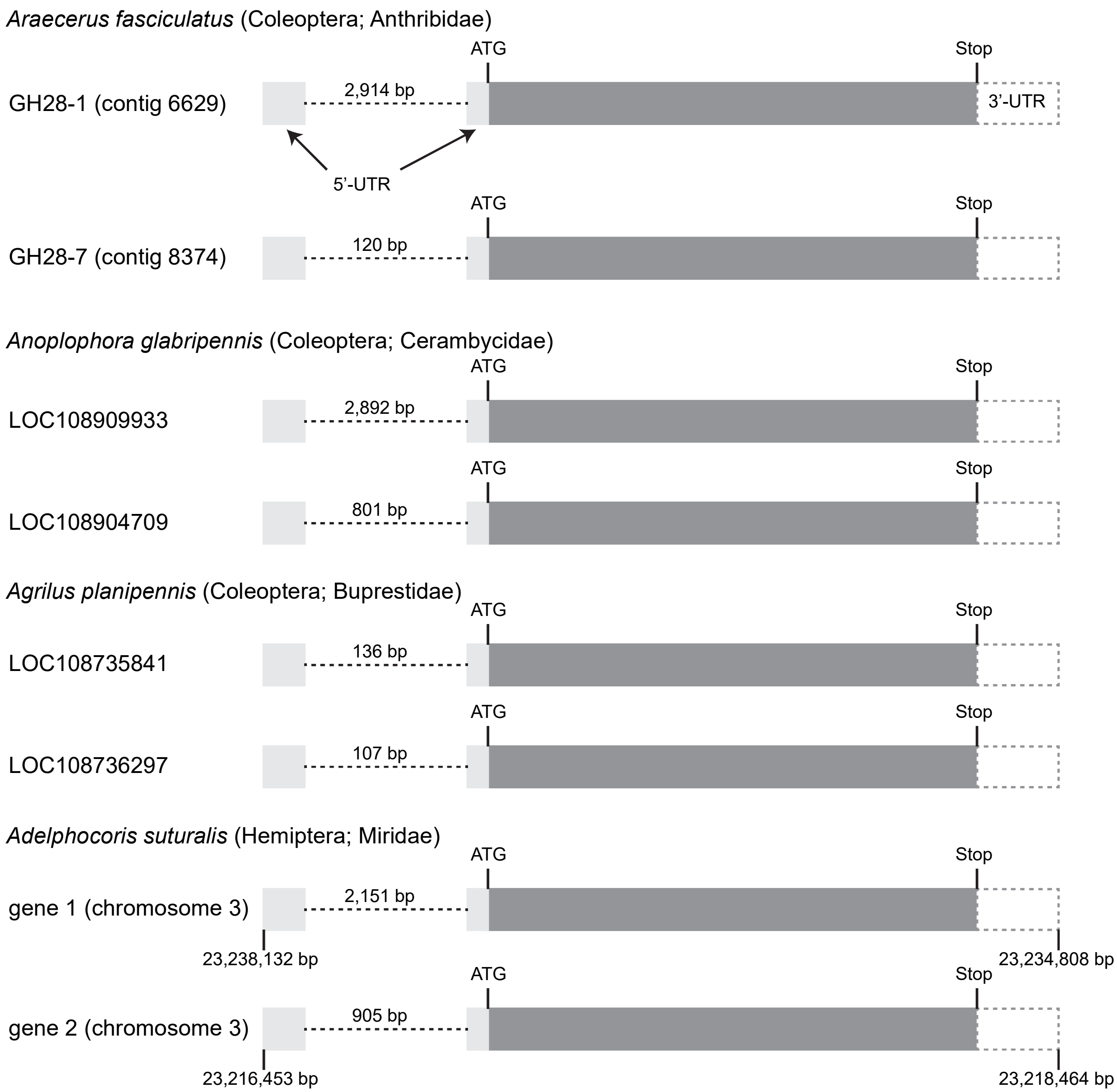

Figure S2. Gene structure comparison of GH28-encoding genes. Genes were derived from the genomes of *Araecerus fasciculatus* with counterparts from *Anoplophora glabripennis* (Cerambycidae; Lamiinae), *Agrilus planipennis* (Buprestidae) and *Adelphocoris suturalis* (Hemiptera; Miridae). Sequences of cDNA encoding GH28 proteins for *A. glabripennis*, *A. planipennis* and *A. suturalis* were recovered from the NCBI database. Gene structures were analyzed by aligning the cDNA sequence to the corresponding genomic region containing the GH28-encoding gene.

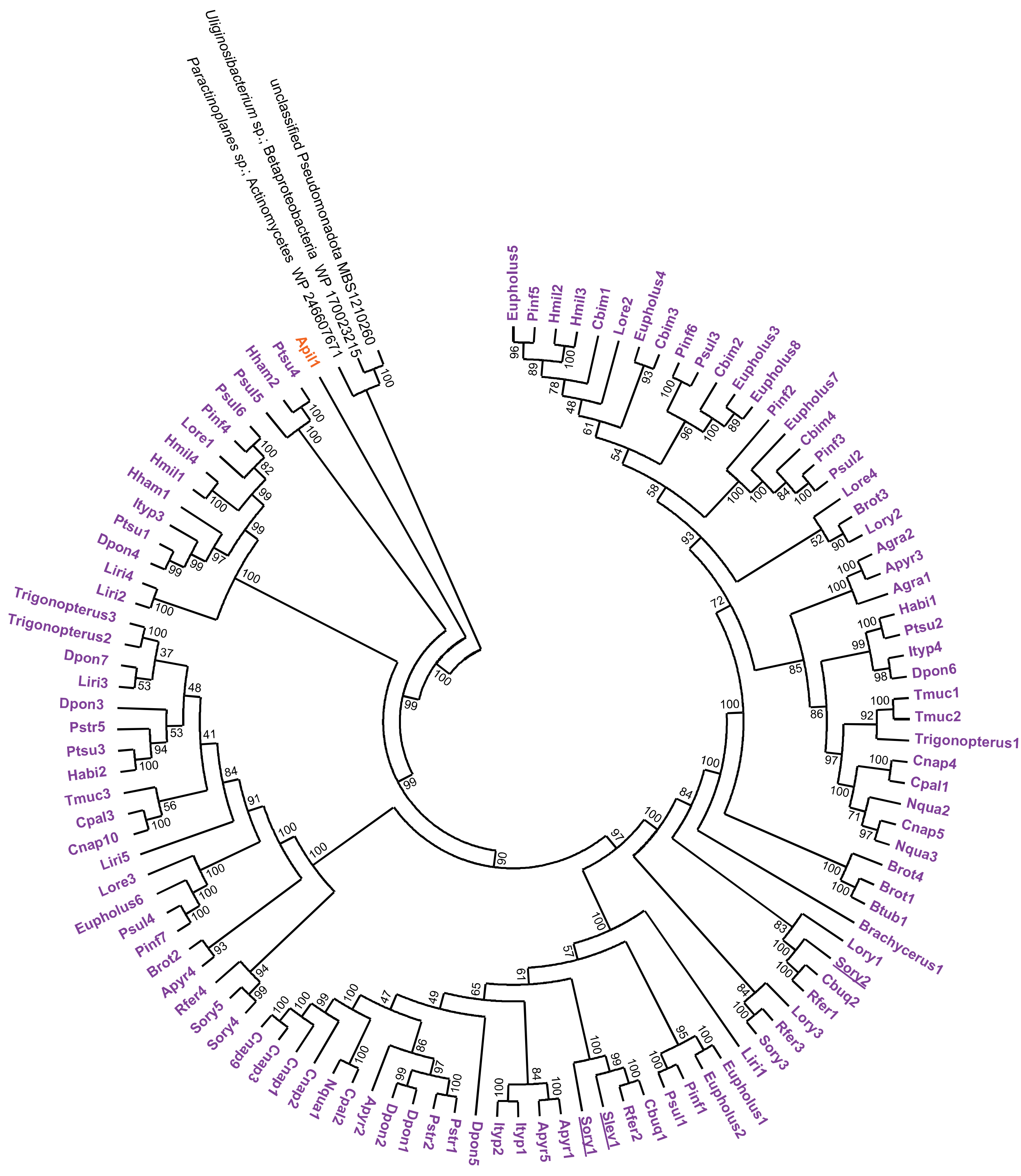

Figure S3. CE8 phylogeny. The phylogenetic relationships of weevil-derived CE8 sequences (Curculionidae: purple; Belidae: orange) with their bacterial counterparts were analyzed using maximum likelihood with 1,000 UltraFast bootstrap replicates in IQ-TREE.

a

```
                            10        20        30        40        50        60        70        80        90       100       110       120
DPO_XP_019760809.2 -------------------------------MALRGSAFLVF--ATL-----------------------------------ISVSLAKVTLTTNGLAATVSNGNVEVVFKADATVSAVKVNGVNIV
DPO_XP_019760811.1 -------------------------------MALWGVVLLLI--ATS-----------------------------------ISVAVATVTLTTSGLTATVSNGDVTVVFNAHARVSSVKIDDVNIV
DPO_XP_048516941.1 ------------------------------MRLILCLLLFVLLTFVG-----------------------------------LSPVDAAVTLTVDGLAATIRNDYVELNFKADATISTVNVQGTNLA
AGR_XP_050296914.1 ------------------------------MNLKIFLTVLVS--LGY-----------------------------------ASDALASVTLTVSGLKANIKNSLVELDFKADSTISAVKVGGKNIA
CAS_CAG9766945.1   ------------------------------MKSITVLLLI----FGY-----------------------------------ISASQAAVSLTVDGLKATLKNKHVELNFNANAQIITAKVDGTSLQ
HHA_KAL1490002.1   ------------------------------MQALSEVVLIL----CL-----------------------------------IGSSFAAVSLKTDGLAATVANDKVSITFKSDSTVSAVTINGVNIA
PVI_PL4_5-trunc    ------------------------------------------------------------------------------------HVDAKVSVTIKGLYLTLKNDHFQAEFSS-GIGTKLYINGVNVI
SSE_PL4-1_5-trunc  -----------------------------------------------------------------------------------------VTVDAAGLTLTLDNGLVKLIFNA-GVATSVTKSGKNII
BKI_XP_033229104.1 -----MDSTIYDSTNSFSSILSVEAVSTNEIRTSENIDSVDEVGTNG-----------------------------------LDSQHPKVSVKINGLTATLDNGLINISFDSHATVSSLYANDQNRV
BKI_XP_033229094.1 ------------------MSAAVAAEPVKKNQKSKNIDSVAEAGTSG-----------------------------------KDSPHPDVSVKINGLTASLDNGLITISFDSRATVSSLYKNGKNRV
BKI_XP_033222742.1 MNKLKKIIRQQQREHRNKNWITKLTRLQTKDNSLWKMTEALKGSIKTTVPILHGPQGLVFSEKDKADVLALQYEQFHRLTTDMINAAVPGVYYEVKGLTAFMNNGIIKIFFDKDATVSALYKNGKNLV
BKI_XP_033222743.1 ----------------------------MMFQIPAYVTLVGIITFTF-----------------------------------GKETSTPVSLVTNGLTAKLDNGLIEVSFRRSGVISSLIKNGNNLV

                     130       140       150       160       170       180       190       200       210       220       230       240       250
DPO_XP_019760809.2 ST--SQKTFYLDWNENGQVTFNPSELKVGENSDSLAHFYWIQNGASNQFHIEFHYVMVDGVSGIYSWAKYINSQSGTVSLGETRTIYRFDSSILTQGTNQARSGTLYLYSYLNTQTKVQDETWQLADG
DPO_XP_019760811.1 ST--TENSFYLDWNENGEVSFSPSSLTVVNDTDSLAHFYWLQDGASDQFEIELHYLMVEGVSGVYSWAKFINSQSANVSLGEARTVYRFNADLLTQGTNQARSGTLYLYSYLNTQTKVQDETWELADG
DPO_XP_048516941.1 AS--GVKSFYLDWNDNGEGVFNPSSIHIVEQTSSRVHFYWLQENIENVFTIELHYVMEEDISGIYSYAKYTNDKSYAVTLGETRMVYRFQASILTQGTNQVRSGTTPTTVDLNQCTTVQDSTWQFPNG
AGR_XP_050296914.1 ST--SERSFYLDWNNNGQGVFKPSSIHIVEQTSSRVHFYWLQENIDNVFRIELHYIMVDGVSGIYSYAKYTNDKSSDLTLGETRVVYRFNPSILTQGSNQARTGTLYTSTYLNKQTKIQDETWQLPDG
CAS_CAG9766945.1   AR--GASSFYLDWNNNGQGYFHPSSIHIVEQTADRVHFYWLQKNIQKVFRIELHYIMEAGLNGVYSYAKYTNDKTYNLTLAETRTVYRFDASIMSQGTNQQRSGILPTTKELNQCTTIQDNTWKFPNG
HHA_KAL1490002.1   ST--KERTFYLDWNANGQGVFAPKTITVVENTSSRAHFYWTQPSSSTTFKIELHYLLEDGVSGIYSWAKYTNDQSGSLSLGETRTIYRMDPAKLTEGTNSARTGTLYLYSYLNQQTKIQDETWQLPDG
PVI_PL4_5-trunc    TNLKNKASFYIDYSDGKDNGFNPTLLKLISETDDLVHVAYYHNS-TTNLSIEEHFLVSSSVSGLYQYVVVRNNAGKTLSVAEIRMVCRFNPSIMKYIHNGKISMETPLYADLEKVTKVQDETWQLADG
SSE_PL4-1_5-trunc  ANLGGKKSFYIDY-DGGKVSFAPQRLDIVEQTTERVHAVYYQDS-TSAFSIEYHLVLEQGISGVYTYVVLSNKASSELTLSEFRTICRFDPSLMNHVHNGKKTIIPPLYAALASMTSIQDETWQLPDG
BKI_XP_033229104.1 ENVAVGETFYLDWHNGTFNHFSPNSLEVISESAKHAHIMYLQDS-PGFLKLEYHLIIKRGLSGIYTYVVKSTNNQDHLLTFGELRVIYRFNPKATHQVTNSIQQGILPDSSVLTNDTLVQDTTWRLKDG
BKI_XP_033229094.1 ENVAAGATFYLDWYNSTFNQFSPSSLQVISKSAKHAHIMYLQDS-PGFLKLEYHLIIERGLSGLYTYVVKSTNNQDHPLTFDELRVIYRFNPKATHQVTNSVQQGILPDSSVLTNDKLIQDTTWRLKNG
BKI_XP_033222742.1 ANVGDDKTFYIDWNNSKTYHFFPSTLTIIRKSVDHAHIMYVQDD-SQFAKIEYHIIMEKGLSGIYSYIRVQNNKRYPISFGLMRIVYRFDPKLMYQITNGVQEGMPPPEFELRKKNEVQDTTWKLKDG
BKI_XP_033222743.1 AN--TEVSGYFDWNSRRNHRFSPSRLDVITKSPQHVHIMYSQDV-RGFLKLEYHIIMEQGISGIYTYVVKATNKNKKPSKLKEMRLIYVFNPKIMYQISNGVQEGTPPTEKELRKLPMIWDATWKLPNG

                        260       270       280       290       300       310       320       330       340       350       360       370       380
DPO_XP_019760809.2 TYYTKYDYAGYLRETSYQGVYGNGYGAFVISPSREYHAGGPLKQDLLIHQDSLIANYFVSSHFGTSGLSAPSGWTHIYGPWLIYFNTGSDSAILSDVANRAQTEKSSWPYSFVNDDAYPRSRGTVTGK
DPO_XP_019760811.1 TYYTKYDYAGYLRNTTYQGVYGSGYGAFLISPSREYHAGGPLKQDLLIHQDSLITNYFVSSHFGTSGLTAPSGWTHIYGPWLLYFNTGTNSAILSDVATQAETEKDSWPYSFVNDDDYPVDRGTVKGT
DPO_XP_048516941.1 TYYSKYDYAAYIRQINYQGVYGNGFGAFVVSPSREYHGGGPLKQDLTVHQECLVANYFLSGHFGTPGLSASPGWTHIYGPFLLYFPTGDDGSIISAVQNQVASEQSKWPYSFVNDAEYPTSRGKVSGN
AGR_XP_050296914.1 TYYTKYDYAGYLRETDYQGVYGNGYGAFVISPSREYHGGGPLKQDLLIHQNSLIANYFISSHFGTAGLTAPSGWSHIYGPWLLYFNTGSDSAILSDVVNRVATEKKSWPYSFVNDSDYPLSRGSVSGT
CAS_CAG9766945.1   TYYTKYDYVAYLRQTNYQGVYGNGFGAFIISPSREYHGGGPLKQDLTVHQECLITNYFVSSHFGTPPTVAAPGWNHIYGPWLIYFPTGNNNAIMSSVVKQVTTEQSRWPYAFVKDDEYPRTRGQVSGK
HHA_KAL1490002.1   TYYTKYDYANYIRETPYQGVYGNGYGAFVISPSREYHAGGPLKQDLLVHQDSLIANYFVSSHFGTTGLTAPAGWTHIYGPWLIYFNTGSDSAILSDLQSRVQTEKNAWPYNFVGDNEYPTARGTVKGT
PVI_PL4_5-trunc    SYYSKYDMADYVKERNFFGVYGGGYGAWFIHPSHEYFSGGPLKQDLIVHQDSLMATYMTGGHFGTPAISMVHGFQKMYGPWLLYFNTGSDSALVSDVQSQAQKEMAKWPYTFVSNPLYPLSRGIVTGT
SSE_PL4-1_5-trunc  SYYSKYDLTGYMRERNFWGIFGNGFGAWTVTASHEYFSGGPLKQDLIVHQDALMAVYMTSTHFGTPSLVAPVGWSKLYGPWLVYFNEGSDDSLVNDVQSRAAQEMAKWPYTWVTNSLYPLKRGAVKGR
BKI_XP_033229104.1 TIYTKYDYAGYIRNSPYLGVFGDGYGAWLLSASREYHSGGALKQDLLVHQDALIANYMTGAHFGTPELKAPVGWSKIYGPWLLYFNEGTDDEMKEDAIRQVKVEQDLWPYKWMHETDYPLKRGSLTGR
BKI_XP_033229094.1 TIYTKYDYAGYIRNSTYLGVFGDGYGAWLISASPEYHSGGPLKQDLLVHEDALIANYMTGSHFGTPALQAPVGWSKIYGPWLLYFNEGTDDKMKADAIRQVKLEKGLWPYKWMKEADYPLKRGALTGR
BKI_XP_033222742.1 NIYSKYDYAGYIRNTYYQGVFGHGYGVWLISASREYHSGGPLKQDLLVHHESLILNCLTAAHFGTPSLVAPPGWSKLYGPWLLYFNTGTDSEVKYNAIYQYKREKKKWPYQWMNESDYPLKRGTLSGR
BKI_XP_033222743.1 TVYSKYDYAGYVRNTSYHGAFGNGFGAWVVSASHEYYSGGPLKQDLLVHQGPLVANYMAGMHFGTPALNAPHGWTKVYGPWLVYFNEGSDDQVKLDAFRQATVEQSLWPYKWMNDKEYPQKRATLQGK

                          390       400       410       420       430       440       450       460       470       480       490       500       510
DPO_XP_019760809.2 ITGQPKAAVMLYDST-ETFDDQQLGYAFTTESDSSGSYTLTNVRPGTYNVVAYPLAGQGSENLATSTVTVAAGGTATVSTLNLPEPGNIIWNIGETDRRSDEFKYSNEPRNYLYETLPPETLTFVIGS
DPO_XP_019760811.1 ITGQPLATIMLYDTEETSYDDQQLGYVFTTESDSSGSYTLKNVRPGIYNVVAYPVAGQGSENEAKTTVTVAAGETVTVSSLDLPEPDDIIWNIGETNRRSSEFKYSAELRNYLYETLPPETLTFTIGT
DPO_XP_048516941.1 VSGQKSATVVLWDSTGEEFALQELGYLYSTQTDSTGYYAFDKVRPGNYRIAAYPTAGLGSDSLDESTVTVEAGATQHVG-FTLAEPDNILWSLGEANRLSSEFKYSDQPRNYQWEWVPPAENTFTIGS
AGR_XP_050296914.1 VSGQKSAMVVLWDDTTKTFEQQSLGHLYNTKTNSDGSFSIANIRPGSYLVAAYPLAGHGSDNLATSSVTVTAGGTANVGTLSLPEPSNIIWSIGETDRRSDQYKYSNEPRNYVYETLPPESLTFTIGS
CAS_CAG9766945.1   VHGQKSATVVLWSSLDEDFDKQQLGYLYSAETDSTGYYIITNVRPGTYRIAAYPTAGSGSDSLAESRVTLAAGAREHVA-LTLNEPKNIIWSLGQANRLSNEFKYSDQPRNYKWATLLPVNLNFTIGS
HHA_KAL1490002.1   VTGQTKAAVVLYDTT-ESFDDQQLGYLFNTETDSSGSFTLTNVRPGTYNIAAYPLAGQGSENLAKATVTVTAGQTATVSALNLPEPSNILWSIGETDRRSDEYKYSDQPRNYVYETLVPETLTFNVGS
PVI_PL4_5-trunc    VRG-KRAMVVL-SPQGEEFDLQTKGYSYNAEAN-NGAFYIKSVRPGTYLVTVYPLAGAAAGTQLESTVTVTAGGTANAGTFTITEP-NVIWQIGEANRRSSEFLYSSEKRNYKWHLLPPANLTFKVGS
SSE_PL4-1_5-trunc  VTTSEKVMVIL-SPQDEQFDIQVKGYSFHAETE-NQAFYIKNVRPGTYTLTAYPITGTGAGALAQQNIYVGEQE-LDVGDININLPQNVLWSLGVANRRSDGYQYSGETRNYLWQTLPPADITFVIGQ
BKI_XP_033229104.1 ITGPPRSMVVLSSSLKEEFDLQTLGYSYSIETNENGDFQLENIRPGIYKLTAYPIAGYGIGFQAEKQITVTAG--ANRATLNLVVPTKVKWSIGETNRRSDSYRYSDKPRNYIWHTLPPANLEFYIGK
BKI_XP_033229094.1 IIGPPRSMVVLSSSLKEEFDLQTLGYSYSTETNGNGDFHLENIRPGRYKLTAYPIAGYGIGFQAEKQITITAG--TIRATLNLSVPTKVKWSIGETNRRSDSYRYSDKPRNYIWHTLAPANLEFRIGK
BKI_XP_033222742.1 ITGPPQSMVVLSSSLRENFDMQTLGYSYSKQTRKDGTFKIGKIRPGMYKLTAYPVAGFGIGYQSEKIIKITAG--MNEAFLNLPVPTTVLWSIGETNRRSDSYRYSNEKRNYKWHLLPPANLNFQIGK
BKI_XP_033222743.1 VTSSARSMVVISSSLKEDFDKQTLGYSYFTETDKTGFFELNKISPGTYKLTAYPIEGYGIGCQAEKVITIREG--MNEASLELPVPTQVMWSIGETNRRSDTYRYSNERRSYVLQSLTPEDLEFEIGK

                               520       530       540       550       560       570       580       590       600       610       620       630
DPO_XP_019760809.2 SNAANDWYYSQSKAGIWTIQYEDTQDGNGRTLRVALAAASQ-------SPHLIVNINGHKIGDI-YFDNDQSVYRSAMQSGKFHSNIFTANNAQIVSGTNVITLQVSKGQVMYDAISLQRG-
DPO_XP_019760811.1 STDADDWYYAQSQAGIWTIEYDDAKDGNTRTLRVALAAASQ-------TPHLIVSVNSHKVGDV-YYGNDQAVYRSAMQSGTFHSNVFTVTNAQVVNGTNTITLQVGKGKVMYDAISLQRG-
DPO_XP_048516941.1 SDPREDWYYAQSQTGSWYIKYQDTADGSARTLRVAIAASSK--------SHLQVIVNGHRVGDT-YFDNDQSIYRSAMQSGRYTSNVYTVSSAQVVSGENTIEFHISIGQVMYDTISMQRS-
AGR_XP_050296914.1 SSISEDWYYAQSKPGVWTVQYQDTQDGNGRTLRVALAAASV-------KPHLIVYLNNHKIGDV-YFGNDQAVYRSAMQSGTFTSLVLTANNAQVVSGTNTLTFQISRGMVMYDTISLQRG-
CAS_CAG9766945.1   SDPKQDWYYAQP-PGHWSIKYQDTADGKGRILRVALAAVSR-------KPHLQVFVNGHRVGNI-YYGNDQSVYRSAMNSGTYYSNVFNVSAAQVVSGENSVSIYMTLGQIMYDVISLQRG-
HHA_KAL1490002.1   SNSKNDWYYSQAKPGIWTITYTDTKDGKGRNLRVALAAASQ-------SPHMIVTLNGHKIGDL-YFENDQSVYRSAMQSGTFYSNIFAANKDQIVDGTNTLTIQISKGQVMYDAISLQTA-
PVI_PL4_5-trunc    SSAAKNWYYAQVQKGTWTIEFTDTADGRARSILVSVAAASV---TSTGKPKLHISFNGKYVATVGQYENDSAIYRSAMQSGNYHYQKIDIPAASVKTGTNVISIDLVQGSFMYDCIVYTRD-
SSE_PL4-1_5-trunc  SNPAADWYYAQTKLGTWTIQFVDKPNGNVRHLLLSIASAST---FSGKNNHVEVAVNGLQLARI-GYANDQSIYREALQSGTYHFESWEIPAATIVDGQNTVTLKLDGGAFMYDSIVFTY--
BKI_XP_033229104.1 SDIHTDWYYAQTKPGTWNIRYTDTVDGQNRILRIGIAAASNGGPTYPGIPTFAVSVNDHLMNEF-HYDNDKSIYRSASQSGSFYFQNIKIPSNLVITGDNVVSLTLRDGMFMYDSINLSSDE
BKI_XP_033229094.1 SDIHTDWYYAQTKPGTWNIRYHDIVDGKNRILRIGIAAASKVNPSDSGIPIFAVSVNGHLMKEF-HYENDKSIYRSAPQSGNFHAEYINIPSNLVTTGDNVISLTVRHGLLMYDSINLSSDE
BKI_XP_033222742.1 SNPANDWYYAQTYKGTWNIIYQDIPNKVNRTLRIGIAAAST---SQSGLPILAVSMNECLITKF-RYQNDHSIYRGAQQSGNFHYANVIIPAKMIKKGRNIVNLSLHSGSVMYDSINLS---
BKI_XP_033222743.1 SSILNDWYYAQTKKGNWNIKYYDTPDQKNRKLRIGLAATSF-------NVTLIVSVNGRQMKQC-EYENDSAIYRCATQSGNFHSEAIAIPANQVVAGENVIQLTLRSGSVMYDSINFSIE-
```

b

```
                            10        20        30        40        50        60        70        80        90       100       110       120
APL_XP_018331663.1 ---MSIKCLIASLVLLLIHGSAGQKCNARSFQRLCVTDGSNVVLENEKLSLTINKAKAQISALYYNSPSDSSIKSTNLLRGGSGYYIAVISVDGKSVNTGPDVGEMKIRQNAEYIDLAFINKNTSNWP
APL_XP_018321695.1 ---MSTKCLIASLVLLLVHTSSGQRCNKLSFQRLCVTDGNDLVLENERLSMTINKTKGQIIELYYNSIIDTTIKSTNLVGGGSNYYLANIDVDGKNASVRPYMGNINITQNAELIDIAFISNDTSIWP
CAS_PL4-1_FL       MCPNIHEKILFCLVFSIFGIVTGQQCKHLSFERLCVTDDDIVVLENEMLSMTINKSMGQITSLYYNSQIDEDIKQTNLLKSGQGYYLGHASLNDTALKMAPDVDNMIITQKAEIIDLAFINKKNAYWP
CAS_PL4-2_FL       MCPNIHKKFLFYLALSIFGIVSGEQCKHLSFERLCVTDDDIVVLENEMLSMTINKSIGQITSLYYNSKTDEDIKQANLLEAGKGYYLGRASLKDEALKIEPEVDTMDVIQNANIIDLAFINKKNAYWP
CAS_PL4-3_FL       MCPNIYKKFLFYLALSILGIVSGEKCNHLSFKRLCVTDAHIVVLENERLSMTINKAKGQITSLHYNSRTDKDIKKTNLLKAGRGYYLGHATLKGEALSVAPEVDTMKVRQNASIIDLAFINKKNASWP

                     130       140       150       160       170       180       190       200       210       220       230       240       250
APL_XP_018331663.1 IHFELHLVLEKDSPIFYYYTIHKYLRD---GYTASQLRWAIRANADPFKYYSVDGKRAGRMPTQQAINSA--ETLQDWTYKLSDGSVYSKYQQISNNEYLHTVFGIYGDRIGLSVLQTHKEWVSGGPF
APL_XP_018321695.1 VYFEFHLVLEKNSSVFYYYSIHKYLRD---GYTAGQLRWAIRANMDIFKYYSVDGKRAGTMPLQRDIKSAQTQTVQDWTYMFKDGTVYSKYQQISSNEDINTVFGIYGNRFGLSLLQTHKEWVSGGPF
CAS_PL4-1_FL       IHFEFHLVLEKNSSLFYYYSIHKYLDDNPDGYSADELRWAIRANKWPFKYYSVDGNRMGAMPTQKDIDEA--QRVQDWTYKLKDGTVYSKYQQISANENINSVFGLYGDHIGFSLLQTSKEWLSGGPF
CAS_PL4-2_FL       IHFEFHLVLEKNSSLFYYYSIHKYFNDNPDGYSADNLRWAIRANNWPFKYYSVDGKRMGPMPAQKDIDEA--QRVQDWTYKLKDGTVYSKYQQISTNENINSVFGIYGDHIGLSLLQTRNEWLSGGPF
CAS_PL4-3_FL       IHFEFHLVLEKNSSLFYYYSIHKYLNDNPDGYTADELRWAIRANPWPFKYYSVDGKRMGVMPIQKDIDEA--QKVQDVTYKLKNGKVYSKYQHISANEGINTVFGLYGDHIGLSLIQTRKEWVSGGPF

                        260       270       280       290       300       310       320       330       340       350       360       370       380
APL_XP_018331663.1 KQSLTTHTGQMLLFCEHTGHYGTPDVVPTKGWSKVYGPIGFYLNDGKSLSQMYLDAQRQLKEEGKKWPYKFVTAPEYNAQGRGSVSGTLNVNGSPNKQWAYLILTEPHVDDPQFETATYMYSTVAATN
APL_XP_018321695.1 KQDLTTHTGHLLLFHEHSGHYGTPELVPTKGWTKVYGPIGLYFNEGLSLSEMYSDAQRQLREEEKKWPYQFVTAPEYNAQGRGSVSGVLTVDGSPNKEWTYLILTEPHVDDPQFESATYMYSAVAGKN
CAS_PL4-1_FL       KQDLTTHTGQMLLFHEHSRHYGTPALVPWKGWTKVYGPVGFYLNEGTGLGDLYADAHRQLKEEESKWPYWFVTAPEYAANGRGSVSGILHVDGVANKEWSYLILTEPHVDDPQYETLTYMYSANAAED
CAS_PL4-2_FL       KQDLTTHTGQMLLFHEHSRHYGTPALVPRKGWTKVYGPAGFYLNEGTDLGDLYADAQRQLKEEESKWPYRFVTAPEYAANGRGSVSGILHVDGVANKEWSYLILTEPHVDDPQYETLTYMYWANAAED
CAS_PL4-3_FL       KQDLTTHTGQMLLFQEQSRHYGTPALAPKKGWSKVYGPIGLYLNEGTGLGHLYADAQRQLKVEESKWPYCFVTAPEYAANGRGSVSGKLHVDGVANKEWSYLILTEPHVDDPQYETLTYMYSANAAKD

                          390       400       410       420       430       440       450       460       470       480       490       500       510
APL_XP_018331663.1 GRFSIPAVRPGNYKLQVIAKGVVGRYIQTNVNVESNKNTNLGTINWQEERHGKQLWQIGVPDRDSGEFKSPPGYPSPIPNLEEYRKFGTWLSYSKTFPNDPDFTVGSSDPAKDWGYFMPMIKTPGFPA
APL_XP_018321695.1 GEFKIPAVRPGTYKLQVIAKGVVGRYIQDNIHVYQKENSDLGTIDWKQETHGELLWQIGVPDRSSAEFKSPPGFPSPIPNLEEYRKFGTWLSYPKTFPNDPDFTVGLSDPAEDWCYFMPTIKTPGFPA
CAS_PL4-1_FL       GEFIIPAVRPGTYKLQVIAKGVSGKYVQNNIHVDANNNTDLGTINWQQESHGQLLWQIGVPDRSSSEFRVPHGFPSPIPNLEEYRKFGTWLSYSKVFPNDIDFTVGKSNPAKDWCYFMPMIKTPGYPA
CAS_PL4-2_FL       GEFIIPAVRPGTYKLQVIAKGVSGKYVQNNIHVDANNNTDLGTINWQQQSYGQVLWQIGVPDRSSSEFRVPPGFPSPIPNLEEYRKFGTWLSYSKVFPNDIDFTVGKSNPAKDWCYFMPMIKTPGYPA
CAS_PL4-3_FL       GKFIIPAVRPGTYKLQVIAKGVSGKYVQNKIHVHVKKNTDLGTINWHQDSHGHLLWQIGVPDRSSSEFRVPPGFQSPIPNLEEYRKFGTWLSYSKVFPNDIDFTVGKSNPAKDWCYFMPMIKTPGYPA

                               520       530       540       550       560       570       580       590       600       610       620       630
APL_XP_018331663.1 QLKMVNITQNTQPTNWKVRFDYSGSTGGTATLTLGFAANVKAGIRAVINGKEILKVTGFD--GPKADSALFRQAAHGIFRQQTVTFSASLLKAKNILEFTPPG-PVSANHFDKMVMWDFLRMEVSN--
APL_XP_018321695.1 QLKLVNVTQNAQPTIWKVRFNYSGSTKGNATLILGFTARVKAGIRALINGEEILEVVDFD--GPQGDSALFRQAAHGIFHQQSVTFGLSLLKDKNILELTPAG-VVNQNHFDRMMMWDFLRMEVSN--
CAS_PL4-1_FL       QLKLVGIEPNTTGTNWKVRFDYDGNGKGNATLTLGLAADVRAGLRVVINSHEIFKIAYLDSYGP-ADSALFRQAAHGIFRQLQVNFSASLLKVQNVLEFTPPGQLISDDHFDNNLMWDFLRMEVASRS
CAS_PL4-2_FL       QLKLVGIEPNTTATNWKVRFDYEGNGKGDATLTLGLAADVKAGLRVVINSHEIFKIAYLDSYGP-ADSALFRQAAHGIFRQLQVNFSASLLKAQNVLEFTPPGQLISDDHFDNNLMWDFLRMEVTSRS
CAS_PL4-3_FL       QLELVGIKQNTTSTSWKVRFDYHGNGKGNATLTLGLAAVVKAGLRVVINSHEIFKIANFD--GP-ADSALFRQAAHGIFRQQKVNFSASLLKAHNLLKFTPPG-AVSANHFDKMVMWDFLRMEVTS--

                               650       660       670
APL_XP_018331663.1 -------------------------------
APL_XP_018321695.1 -------------------------------
CAS_PL4-1_FL       NYMNRGVPLMALVFGLSTLFSLSRILFENNL
CAS_PL4-2_FL       NYMNRCAPLMELVFGLLTLFSLSRILFENNL
CAS_PL4-3_FL       -------------------------------
```

Figure S4. PL4_4 gene structure across insects. (a) weevil (DPO, *Dendroctonus ponderosae*; AGR, *Anthonomus grandis*; CAS, *Ceutorhynchus assimilis*; HHA, *Hypothenemus hampei*; PVI, *Perapion violaceum*), leaf beetle (SSE, *Syneta seriata*) and gall wasp (BKI, *Belonocnema kinseyi*) counterparts were compared. *Apion frumentarium* PL4-3 was used to annotate PVI PL4 gene. (b) jewel beetle (APL, *Agrilus planipennis*) and leaf beetle (CAS, *Crioceris asparagi*) counterparts were compared. Intron positions are highlighted by colored amino acids and the corresponding intron phases 0, 1 and 2 are indicated by green, red and blue respectively.

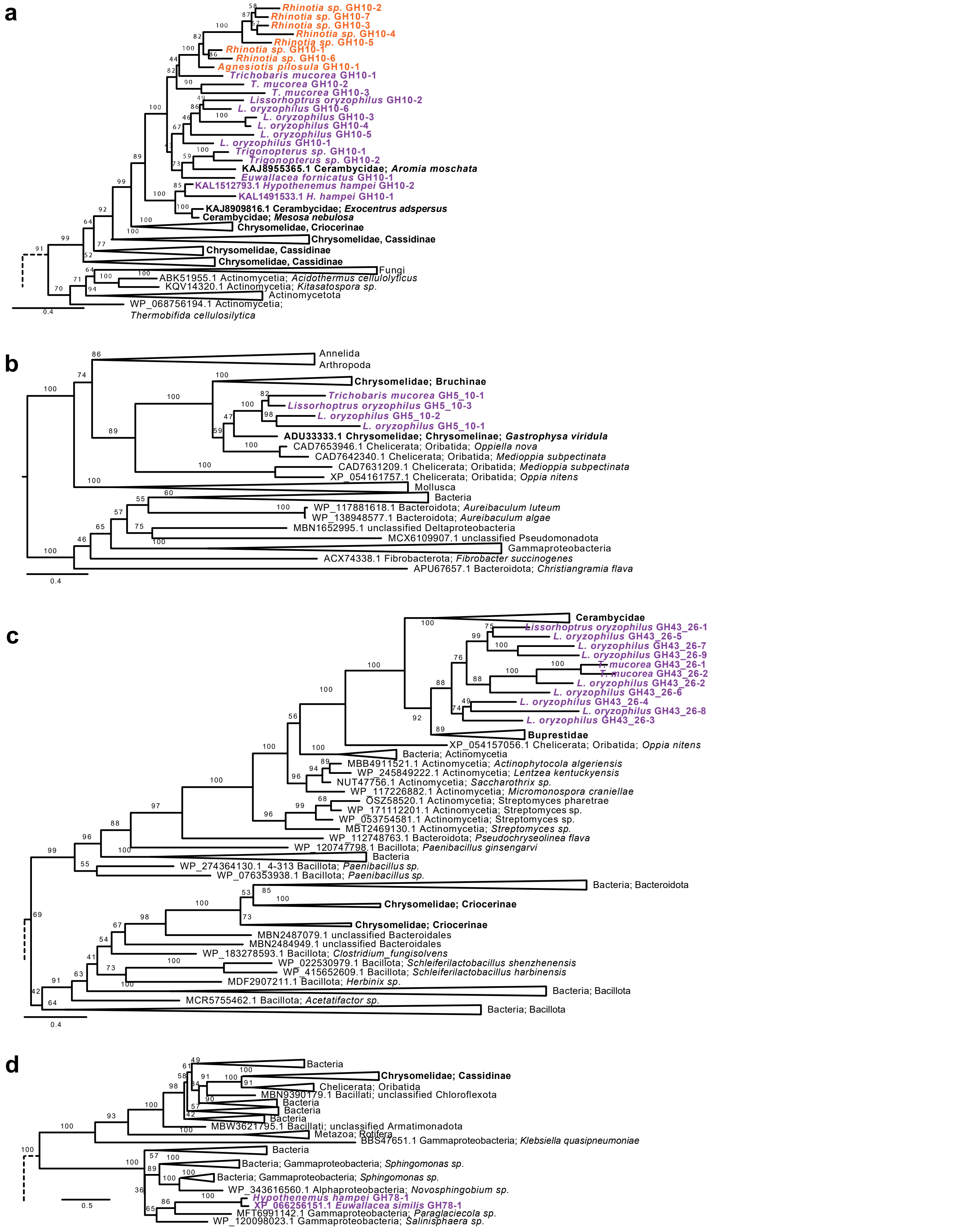

Figure S5. Phylogenetic relationships of PCWDE families that are patchy distributed in weevils and other beetles. (a) Sequences of weevil GH10 (Belidae, orange; Curculionidae, purple) are nesting within a monophyletic clade of beetle counterparts indicating common ancestry and acquisition from bacteria.
(b) GH5_10 from Curculionidae (purple) cluster together with leaf beetle counterparts as well as sequences from oribatid mites collectively forming a sister-clade with mollusks, indicating that insect GH5_10 genes have multiple independent evolutionary origins with intra-phylum HGT between mites and beetles playing a role.
(c) GH43_26 from Curculionidae (purple) group together with representatives from Cerambycidae,Buprestidae and oribatid mites but separate from Chrysomelidae. This pattern is best explained by a coleopteran acquisition from an Actinomycetia or mite donor followed by a more recent acquisition in leaf beetles from a bacterium of the Bacteroidota. (d) GH78 proteins from Curculionidae (purple) are separated from Chrysomelidae counterparts, indicating independent acquisition from different bacterial donors, again with an intra-phylum HGT between mites and beetles to be involved. Interestingly, beside H. hampei, we found a GH78 encoding gene in the draft genome of *E. similis* but not in the sister-species *E. fornicatus*.

a

```
                          10        20        30        40        50        60        70        80        90        100       110       120       130
EFO_XP_066149587 ---MKVILLLTLVGSI-SCGLRDYAKKIYIGTALSNNHLNSDATYNQLAYTQFNAMTPENQMKIDATEPRQNQFNYAPADELVSFAQNHNMKIRGHTLVWHSQVPSWMSALTGTNLQSAMVNHINNVVGHYKGK
HHA_KAL1491533.1 ---MKLIVLFALFGSI-AAHLRDHANKIYIGSALAPSHFT-DAQYSTIAAEEFNSLTPENEMKWANVEPSKGKYNYGPAEKLVEFAQQYDMIVRGHTLIWHQEVPDWVSVLSGDDLHEVMIDHITTLVTHFKGS
HHA_KAL1512793.1 ---MKLIIILFALIGSI-AAHFKDHANKIYIGSALAPDHLS-DSQYSSIAAEEFNSLTPENEMKWDAVEPSQGNYNYGPADQLVQFGQQNGMKIRGHTLVWHSQVPSWVSGLSGDTLHQTMISHITNVVTHFKGS
OGA_GH10-2       -MKATLLFLFVFIGAI-SCHMRDRARRIYIGTCLNKEFMN-DAQYKTIAAQEFNCMTPGNEMKWASVEWNRGRINYGPADQLVQFAEQHGMKVRGHTLIWHNQIPRYLNELDKNTLHQTMLNHIWDEVTHFKGK
OGA_GH10-3       -MKTILLFLFVCIGAI-SCHLRDKAHKIYIGTALMASQLS-NNQYRTIAGQEFNVMTCDNEMKWDNVEWTRGRYNYARAEQLVQFAQQNRMKIRGHNLIWHRQTPPWLNQLDKNSLQQSMHARIVNEVTHFKGR
CAL_GH10-6       MKGLRLALLLLFVGSIGAANFRDLTKRILVGSILGYSWMN-ESHVYNWTCKMFNKMTCENEMKWAVYEPERGKINHGPADLIVEYAEANKMKIRGHTLVWHQQLPPWVKTLNKTELHTVMITRIKEMMTRYKGR
EAD_KAJ8909816.1 --MLKVLILLTILGSI-AAHLKDHASQIYIGCALSPSHFS-DSQYQSIAAAEFNSLTPENEMKWDAVEASQGSVNYAQADQLVQFGKQNGMKIRGHTLVWHSQVPGWVSGLSGDTLHQTMISHITDEVTHFKGS
AMO_KAJ8955365.1 ---MKILLLLGLVGSI-SCAFRDHANKIYIGAAVSPTYFS-DATYQSLAGAQFNFLTPENEMKIDATEPSQGQFNYAQADELVQFAQTNNMKVRGHTLVWHSQVPSWMQSLTGTALEEAMKNHISNVMGRYKGG

                 140       150       160       170       180       190       200       210       220       230       240       250       260
EFO_XP_066149587 LYAWDVVNEIFNEDGS-FRNSIWTQAMGQNFVALAFKTAAAADPNAKLYINDYNIEGQNAKSNGLYALVSKLKSEGVPIHGVGFQAHFTVGQVPSTLKANLERFTALGLEVAITELDVGENS---RADDAAQAA
HHA_KAL1491533.1 IYAWDVLNEILDEDGS-FRKTIWYNNFQTQYIADAFEAAAAADPDAKLYINDYSTEYSNNKSEALYNLVKELKSQGVPIHGVGFQTHSSVGKVPSDFASTLKKFTALGVEVAITELDIRLNG----QSEDAQTE
HHA_KAL1512793.1 IYAWDVVNEIFNEDGS-YRSSLWYNNFQTSFVADAFQAAAAADPSAKLYINDYNVEYTNAKSNALYNLVKELKSQGVPIHGVGFQSHLAVGQIPSDFATNLARFTALGVDVAITELDIKQNG----QSQDAQAA
OGA_GH10-2       IYAWDVVNEVFNEDGS-LRNSIWTQHFGESFIADAFRRAHEADPNAKLYINDYNVAPKCRKSDGLYNLVKRLRAQGVPIHGVGIQAHMGTGRVPRDLADNMRRFSDLGLDVALTEVDLPINGAINQQSRHQQAM
OGA_GH10-3       IFAWDVINEPMEEDGT-LRKSIFYQHFGESYIADAFRLAHQADPNAKLYINDYSIEGRNRKSEGLYNLVKRLKAQGVPIHGVGFQGHMAAGLVPHELADTMRRFSELGLDVAITEMDLGIHGAINAQTRHQQAM
CAL_GH10-6       IYAYDVVNEVWANDGKGLKKTLWFNALKDTYVPEAFHLARKMDPAAKLYINEYGVEGNNSKTVSFYNYVKTLKSQGVPIDGVSFQCHFKAGHVPVDFQKVLEWFTTLDVDVGITELDIGIANT-SAAQLKQQAT
EAD_KAJ8909816.1 IYAWDVVNEMFNEDGT-YRQSLWYNNFQTNFVGDAFKAAAAADPGAKLYINDYNIEGINAKSTGLYNLVKELKGQGVPIHGVGFQAHLSTGQVPSDLAANLQRFTALGLDVAITELDIKQNG----QSQEAQAQ
AMO_KAJ8955365.1 IYAWDVVNEVFNEDGT-LRDSIWTQAMQSGFIATAFQAAREADPNAKLYINDYNIEGVNAKSTGLYNLVQELKTQGVPIDGVGFQAHFTAGSVPTDLQTNLQRFADLGVDVAITELDIGGAG-----QEAQQAT

                 270       280       290       300       310       320
EFO_XP_066149587 DYAKVVQTCVSVNGCVGVSIWGITDNYSWRASTTPLAFDSNGNAKAAVNAMINVLQ
HHA_KAL1491533.1 AFVEVVKDCVSVSGCVGVTFWEFIDKYSWISEETPCPWDSNYQAKPAVAAIEAVLE
HHA_KAL1512793.1 AYSEVIKDCLSVNGCVGVTIWGFTDKYSWISSDTACPWDSNYQAKPAVAAIEAALQ
OGA_GH10-2       DYANVIRACLSVPRCVGVTIWGFIDKYSWINHGEPLPWDNNYRPKEAVSAITNALQ
OGA_GH10-3       DYANVVKACLSVPRCVGVTIWEFVDKFSWIPNLAPLPWDNNYQPKEAVNALYNALQ
CAL_GH10-6       DYAFVYNSCENVKRCVSVTAWGFTDKFSWLKPTKPCMWDEEFKPKPAVAAVEAVLK
EAD_KAJ8909816.1 AYASVVNACTSTSGCVGVTIWGFNDQYSWI-NDNPCPWDSNYQAKPAVAAIEGALK
AMO_KAJ8955365.1 DYATVIQDCLAVSRCVGVTIWGIMDKYSWRASTNPLPFDDNGNAKAAATAIEGALQ
```

b

```
                              10        20        30        40        50        60        70        80        90        100       110       120       130
MIM_XP_045971499.1---MLFSTFFATALLSVSSLI---SSVAAQTPGACSGDCNVHDPALIRRTSDGKYFRFSTGNKITIATSSALAGPWTNQGSAITGG-SSIALTGNDDLWAPDIQKVGDYYYLYYSVSTFGSQASAIGVARST
TME_XP_002836910.1------MMLLKLILLLVAFVS---TAISYGNPGACSNACNVHDPAVIRRDSDGTYFRFSTGNKIQIATSSSLSGPWTIKGSALSMG-SKINLAGNQDLWAPDVTKVGDNYYLYYSVSTFGSQESAIGVATSK
DAMKAK2598229.1   ---MFTLSNLTRGLVALALFALPGTQAAYPNPGACSGDCFAHDPALIKRSSDGKYFRFNTGGKIMIYNSDSLSGPWTYQGAAIPSG-SSINLPGKDDLWAPDVRLVGDTYYMYYAVSAFGSQASAIGVATSK
ACO_GH43_6-1      -------MKILIASILVFSITSN-EVLAYANAGDCTGDCNVHDPSLIQRASDGKWFRFSTHNLLAIATADDLGGPWTNIGSVLVNK-SKIDLPGNDNPWAPDVHYYKGKYYCFYAISKWGSRHSSIGVAVSD
ACO_GH43_6-2      MFCSKNISQSLFLCLLLNFCEIS-QVFSYSNPGGCSGDCNIHDPALIQRKSDGKWFRYTTHQLIGIATADDISGPWTNVGSVLVNK-SKIDLPGNTDPWAPDIKYYKGLYWCFYSICKLGTRNAAVGVAVSN
ACO_GH43_6-3      ---MSSTGILSVACIATFLCLASYSSAAYPNPGACSGSCNIHDPSVIKRSSDGKWFRFTSHNLVGIATASSIAGPWTDVGSVLHNGKSIINHANNADPWAPDVQKYGNYYWCFYAISSFGSQDSVIGVAVSS
ACO_GH43_6-4      ---MSNTGIISVACIATFLCLASYSSASYPNPGACSGSCHIHDPSVIRRSSDGKWFRFTSHKLIGIATANSIAGPWTDVGSVLHSGKSIIKHANNADPWAPDVQKYGNYYWCFYAISSFGSQDSVIGVAVST
ACO_GH43_6-5      ---MANPGIFSVACIATVLCLVSYASAGYPNPGACSGSCNIHDPSVIRRVSDGKWFRFTSHNLIGIATADSIAGPWTDVGSVLHNGKSIINHVNNADPWAPDVQKYGSYYWCFYAISTFGSQDSVIGVAVSS
ACO_GH43_6-6      -----MFGISSLILIAIYYCNS--AFAAYPQPGPCTGYCFTHDPSLIQRESDKKWFRFTTHFLVGISTADDIAGPWTNVGSVLVNQ-SIIDLPGNQNIWAPDVKYYKGKYYCFYAVSRWGDQHSALGVAVSD

                            140       150       160       170       180       190       200       210       220       230       240       250       260
MIM_XP_045971499.1TMEVGSWTDLGSTGIASSSGSAYNAIDSNLILAGS-TYYATFGSFWGDIYQVPMASTPTKTAGT-SIQVAYNSS--GSHAVEGSFTYYRSPYYYLFFSSGTCCGF-DTSRPAQGAEYRINVCRSSSVTGPYV
TME_XP_002836910.1TMDPGTWTDYGRTGVVSHAGSAYNAIDGNVVLAGDGSYYMSFGSFWGDIYNVPMNNPPLTASGS-STRIAYNST--GSHAIEGSFIWYRSPYYYLFFSAGTCCGY-DSNRPAPGMEYRINVCRSTSVNREYV
DAMKAK2598229.1   TLEAGSWTDHGATGISSKAGSNYNAIDPNLVEKADGSFVLNFGSFWNDLYQVPMASTPTKAAGS-SYNIAFNSS--GTHAEEGPFIYYRAPYYYLFWSSGICCGY-DTTKPASGEEYRINVCRSQSVNGPFV
ACO_GH43_6-1      NMEPGTWDDFGAIGIISLNGDIYTAIDPSFIETVEGRTFISFGSYLTDIYAVELET-PYLVNGT-LTHISYNNS--GDHGQEGSYLYFNDGYYYLFFSAGLCCKFNPASLPAPGYEYHINVCRSNDVLGPYV
ACO_GH43_6-2      TMEPGSWVDKGSVGLTSKDGGIYAAIDQTFIETPEGKMYLSFGSYLQDIFLIEMEN-PEKVIGE-PIQISFNSTYPKHAAEEGSYLYYHEGYYYLFFSAGQCCNY-VGKMPKQGYEYRIVVCRSNSVTGPYV
ACO_GH43_6-3      TMEPGSWVDKGAV-FSSKSGDKYNAIDPNFISTSNGQMYMSFGSFWEDLFIINMAI-PEKVNGN-AVSISFNST--GTHAEEGSYLYYYSGYYYLFFSSGQCCNY-AASMPAKGEEYRINVCRSLSVTGPYV
ACO_GH43_6-4      TMEPGTWVDKGAV-FSSKHGDKYNAIDPNFIQTGNGQMHLSFGSFWTDLYVVSMSS-PETVHGN-PVHTSYNNT--GTHAEEGSYIYYHSGYYYLFFSSGQCCGY-IASMPKQGAEYRVNVCRSKSVTGPYV
ACO_GH43_6-5      TMEPGSWVDKGAL-ISSKTGDDYNAIDPNFIYAGNGQMYLSFGSFCLDLFVVSMVY-PERINGN-PIHTSYNTSTSGLHGEEGSYIYYYKGYYYLFFSSGQCCNY-IASMPRQGEEYSIHVCRSESVTGPYV
ACO_GH43_6-6      TMDPGTWTDLGYVGIISLPGARYNAIDSSWTETPEGKHYMNFGSYNDDIFIIEMDT-PVKVNGTDLINIAYNPA--GSHSYEGSYMYYREGYYYLFMSAGQCCKFKPANLPPAGQEYHILVCRSENIEGPFY

                          270       280       290       300       310       320       330
MIM_XP_045971499.1DATGKSCTAGGGTTVLASHDNVYGPGGQGIYPDTTQGGAVLYYHYVNTNIGYGDGQKLFGWNLLSWSTGWPVPT
TME_XP_002836910.1DKNGVSCTAKGGSTVLASHDNVYGPGGQGVFLDNVSGGEVLYYHYVDTTIGYGDGQKRFGWNLINWSTGWPVV-
DAMKAK2598229.1   DQRGVNCAQGGGTTVLASHGKVYGPGGQGVYADAAHGGAVLYYHYADTSIGYADAKYQFGWNGLSWSTGWPVAT
ACO_GH43_6-1      DREGRSCLEGGGTTVLGSHDKVYAAGGQGVYNN------ILYYHYVHTDYGYKDNDKRFGWNEITWVDGWPTI-
ACO_GH43_6-2      DQTGRECLKGGGTTVLASHGKVFGPGGQGVYND------ILYYHYVDTDYGYGDATKRFGWNKIAWVDGWPTV-
ACO_GH43_6-3      DASGKKCTEGGGTTIYTSHGKIFGPGGGGVYDN------ILYYHYVNTDNGYADDKKLFGWNELAWSNGWPSVK
ACO_GH43_6-4      DASGKSCLQGGGTTIYTSHGKIFGPGGGGVYDN------ILYYHYVNTNNGYADDKKLFGWNELAWSNGWPSVK
ACO_GH43_6-5      DASGKSCLQGGGTIIYASHDKIFGPGGGGVYDN------ILYYHYVNTDNGYADDKKLFGWNELTWSNGWPSVI
ACO_GH43_6-6      DKNGVSCLEGGGTTIYASYNTTYAPGGQGVYND------ILYYHYVDTTISYLDNDKRFGWNKLSWEDGWPVVI
```

c

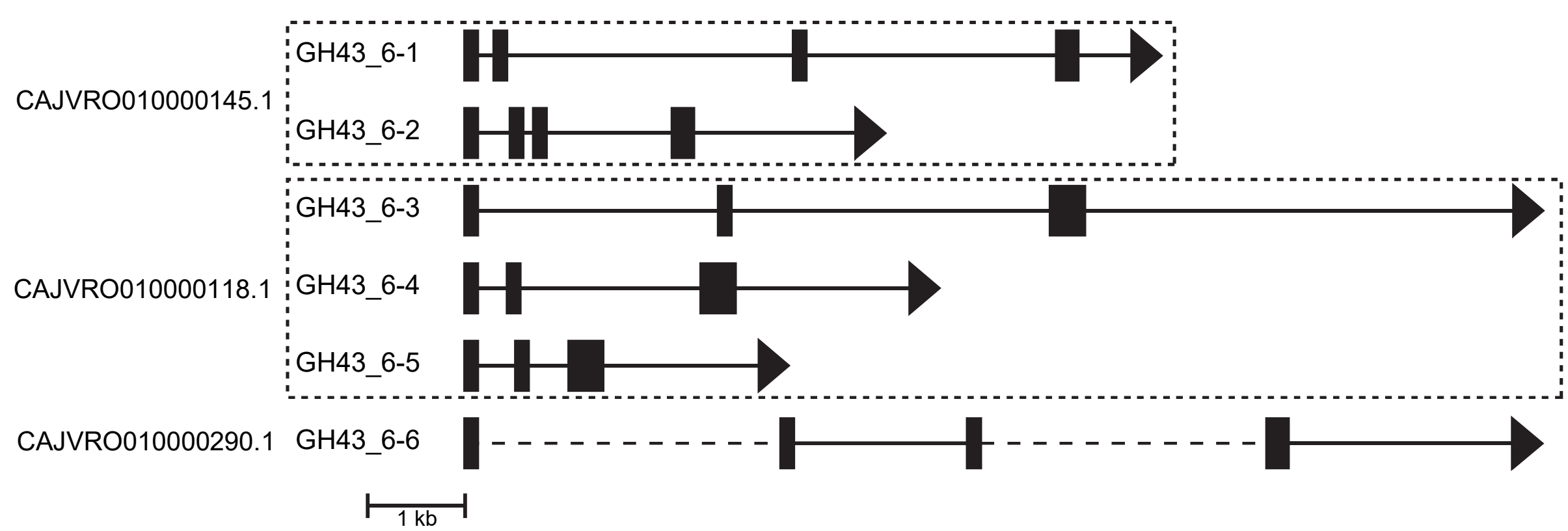

Figure S6. Gene structures across eucaryotes. (a) GH10 gene structure comparing weevil (EFO, *Euwallacea fornicatus*; HHA, *Hypothenemus hampei*), leaf beetle (OGA, *Oulema gallaeciana*; CAL, *Chelymorpha alternans*) and longhorned beetle (EAD, *Exocentrus adspersus*; AMO, *Aromia moschata*) counterparts. (b) GH43_6 gene structure comparing fungal (MIM, *Morchella importuna*; TME, *Tuber melanosporum*; DAM, *Diaporthe amygdali*) and weevil (ACO, *Apoderus coryli*) counterparts. Intron positions are highlighted by colored amino acids and the corresponding intron phases 0, 1 and 2 are indicated by green, red and blue respectively. (c) The structure of *A. coryli* GH43_6 genes is presented. Exons are depicted as blocks, and introns are represented by thin lines. Each gene structure is drawn to scale, with a scale bar indicating 1 kb.

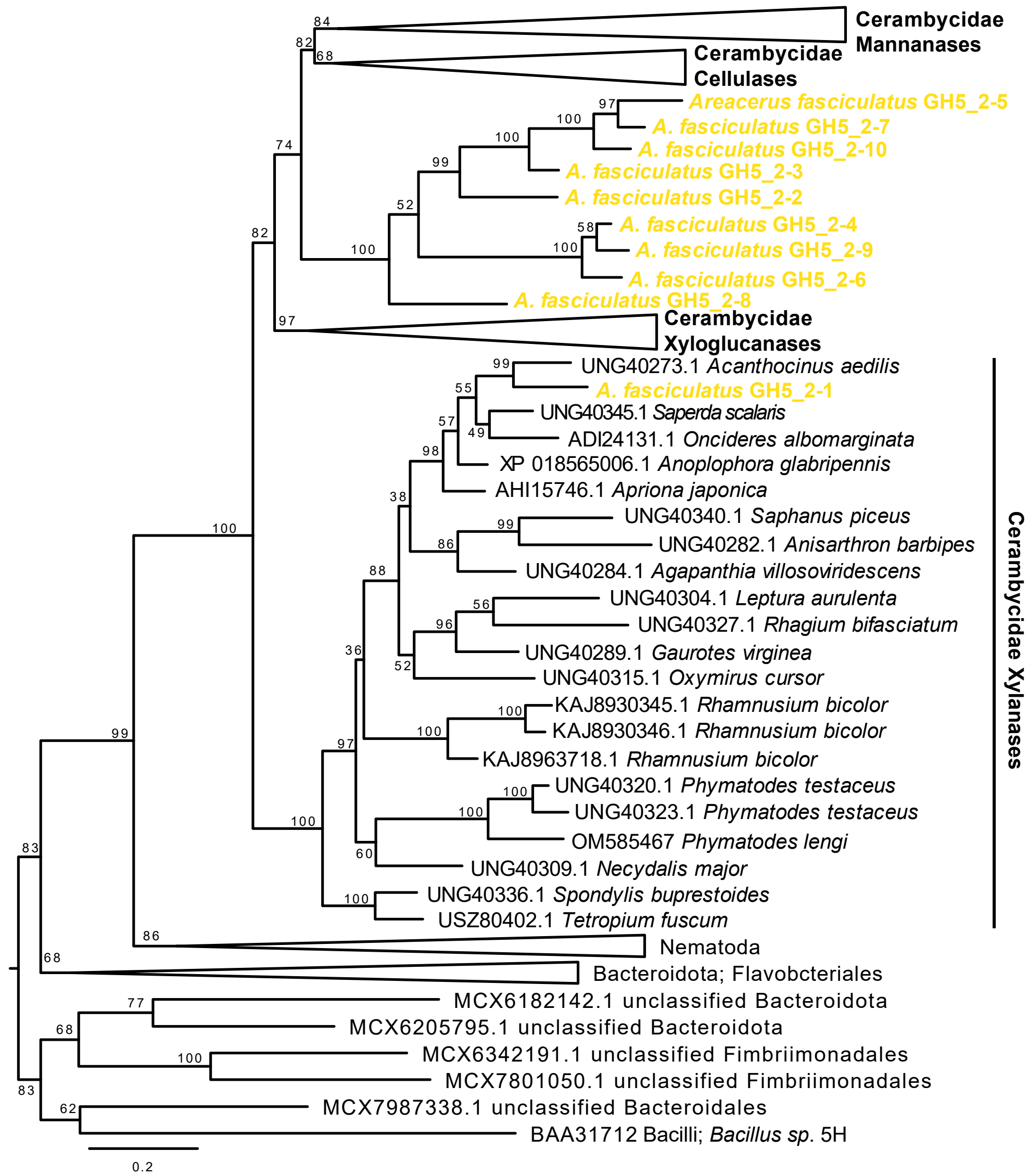

Figure S7. Phylogenetic relationships of GH5_2 proteins in *A. fasciculatus* compared to homologs in longhorned beetles, nematodes and microbes. GH5_2 sequences, retrieved from *A. fasciculatus* (highlighted in yellow), along with those from Cerambycidae, plant-parasitic nematodes, and bacteria, were subjected to maximum likelihood (ML) analysis. The LG model, incorporating a FreeRate model with nine categories (+R9), emerged as the optimal model for protein evolution. UltraFast Bootstrap values are indicated alongside branches, and the tree is rooted at midpoint. GH5_2 sequences from *Araecerus* form a monophyletic group, except for GH5_2-1, which nests within a larger clade of Cerambycidae-derived sequences. GH5_2-1 clusters with Cerambycidae-derived sequences for which the products were characterized as xylanases. Collectively, such tree topology indicates insect-to-insect HGT.

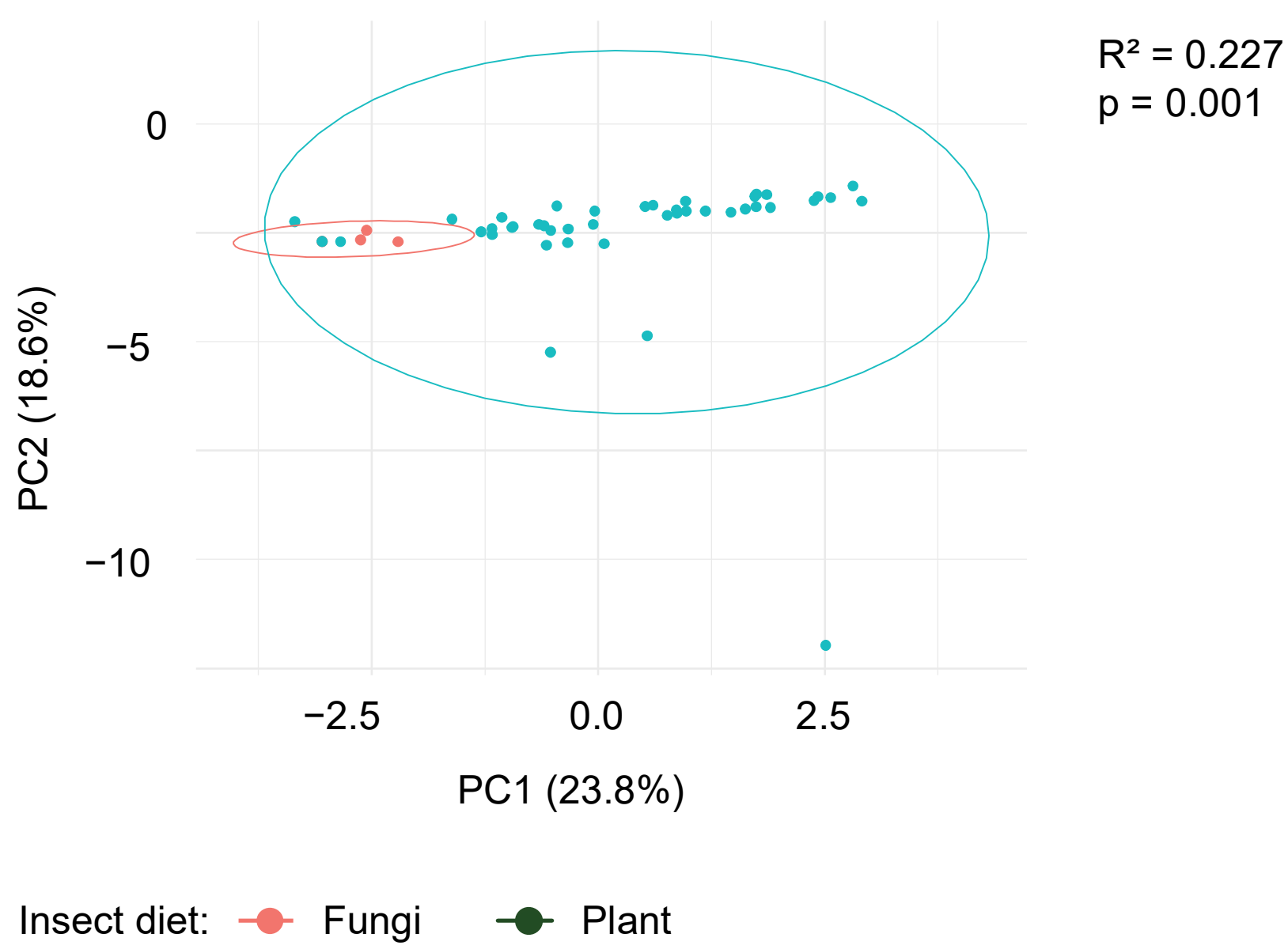

Figure S8. Separation on plant-feeding and fungal-feeding niches based on PCWDE gene family sizes. PCA was performed using the number of PCWDE gene family as shown in Figure 1 and Table S4. A total of 43 plant-feeders and 5 fungal feeders are analyzed to assess group separation based on dietary preferences. Species are color-coded to feeding niche. A permutational MANOVA confirmed significant separation between plant- and fungal-feeders.

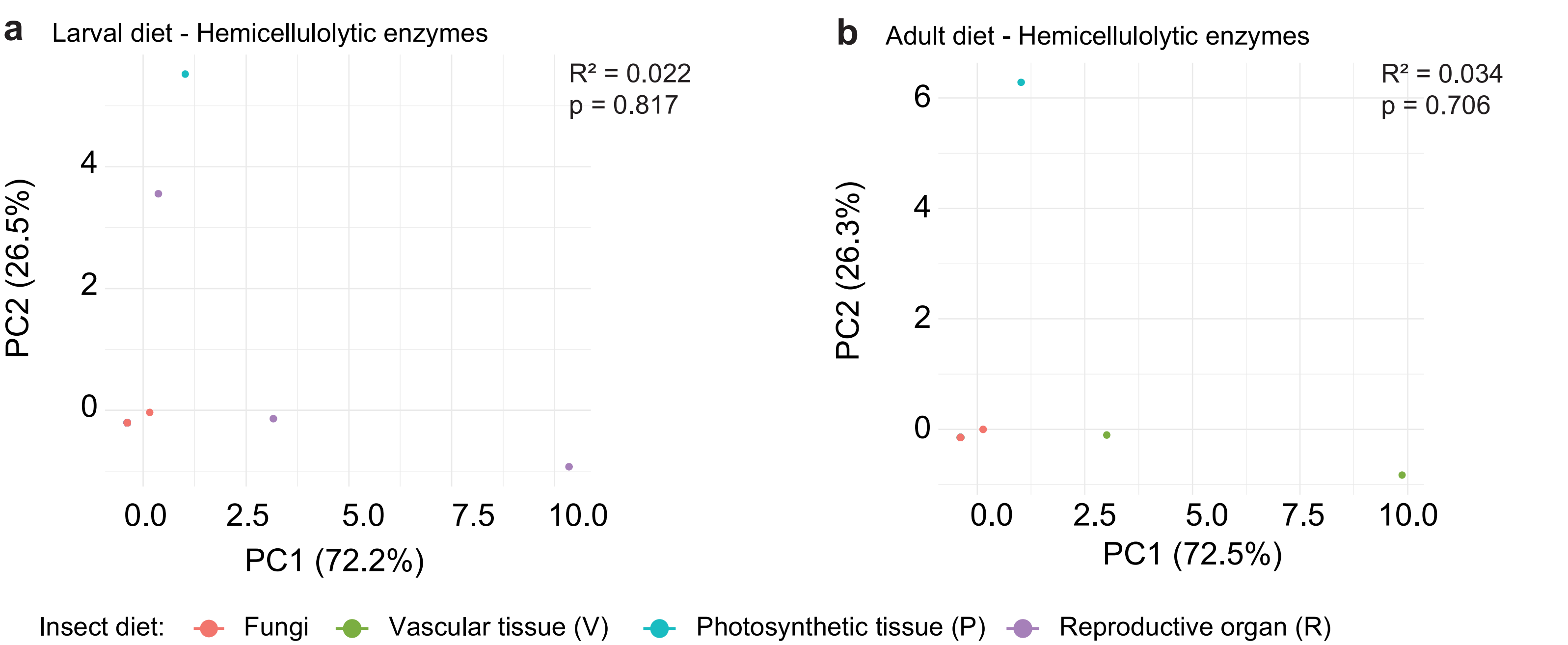

Figure S9. Separation of larvae and adult feeding niches based on hemicellulolytic enzymes. PCA was performed to assess group separation based on dietary preferences, using the number of hemicellulolytic enzymes as shown in Figure1 and Table S4. Species are colored-coded by feeding niches. For more detailed PCA analyses, plant-feeding species were grouped into R (reproductive tissue), P (photosynthetic tissue), and V (vascular tissue) groups (see Figure 1 and Table S4 for details). Because some species shift dietary preferences between life stages, the number of species in each group differs between larvae and adults (larvae: P = 4, R = 9, V = 26; adults: P = 21, R = 7, V =7). To account for these differences, clustering analyses based on this fine-scale dietary classification were conducted separately for larval and adult stages. A permutational MANOVA was used to test for significant separation among dietary groups.
