# Supplementary material for: Evolutionary dynamics of plant cell wall-degrading enzymes reflects feeding ecology in weevils
Source: Commun Biol. 2026 Sep 23;9:1243. doi: 10.1038/s42003-026-10927-0 (PMC13601563; doi:10.1038/s42003-026-10927-0)
Supplement: Supplementary file 3 [file 42003_2026_10927_MOESM3_ESM.pdf]

Description of Additional Supplementary Files

File name: Supplementary Data 1
Description: Supplementary tables (Table S1 – Table S6)

File name: Supplementary Data 2
Description: This file contains the draft genome assembly (unpolished and polished with final error correction) of *A. fasciculatus*.

File name: Supplementary Data 3
Description: This file contains the codon-based nucleotide alignment (FASTA file), the IQ-TREE log file and the resulting tree file (Newick file) used to generate **Fig. 1**.

File name: Supplementary Data 4
Description: This file contains the amino acid alignments (FASTA file) and the resulting tree files (Newick format) of all the phylogenetic analyses of PCWDE families presented in the paper.

The supplementary data 2-4 are freely accessible on EDMOND - the Open Research Data Repository of the Max Planck Society with DOI: https://doi.org/10.17617/3.5MTAYX.
